# A comparison of the nutritional quality of products offered by the top packaged food and beverage companies in Canada

**DOI:** 10.1186/s12889-020-08828-w

**Published:** 2020-05-11

**Authors:** Laura Vergeer, Lana Vanderlee, Mavra Ahmed, Beatriz Franco-Arellano, Christine Mulligan, Kacie Dickinson, Mary R. L’Abbé

**Affiliations:** 1 Department of Nutritional Sciences, Faculty of Medicine, University of Toronto, 1 King’s College Circle, Toronto, Ontario M5S 1A8 Canada; 2 School of Public Health and Health Systems, University of Waterloo, 200 University Avenue West, Waterloo, Ontario N2L 3G1 Canada; 3 Nutrition and Dietetics, College of Nursing and Health Sciences, Flinders University, G.P.O, Box 2100, Adelaide, SA 5001 Australia

**Keywords:** Food company, Nutritional quality, Food supply, Food environment, Nutrient profile

## Abstract

**Background:**

Canada’s food supply is abundant in less healthy products, increasing Canadians’ risk of obesity and non-communicable diseases. Food companies strongly influence the food supply; however, no studies have examined differences in the healthfulness of products offered by various companies in Canada. This study aimed to compare the nutritional quality of products offered by the top packaged food and beverage companies in Canada.

**Methods:**

Twenty-two top packaged food and beverage manufacturing companies were selected, representing > 50% of the Canadian market share in 2018. Nutritional information for products (*n* = 8277) was sourced from the University of Toronto Food Label Information Program 2017 database. Descriptive analyses examined the nutritional quality of products based on: 1) the Health Star Rating (HSR) system; 2) calories, sodium, saturated fat and total sugars per 100 g (or mL) and per reference amounts (RAs) defined by Health Canada; and 3) “high in” thresholds for sodium, saturated fat and total sugars proposed by Health Canada for pending front-of-package labelling regulations. Kruskal-Wallis tests compared HSRs of products between companies.

**Results:**

Mean HSRs of companies’ total product offerings ranged from 1.9 to 3.6 (out of 5.0). Differences in HSRs of products between companies were significant overall and for 19 of 22 food categories (*P* < 0.05), particularly for fats/oils and beverages. Calories, sodium, saturated fat and total sugars contents varied widely between companies for several food categories, and depending on whether they were examined per 100 g (or mL) or RA. Additionally, 66.4% of all products exceeded ≥1 of Health Canada’s “high in” thresholds for sodium (31.7%), saturated fat (28.3%) and/or sugars (28.4%). The proportion of products offered by a company that exceeded at least one of these thresholds ranged from 38.5 to 97.5%.

**Conclusions:**

The nutritional quality of products offered by leading packaged food and beverage manufacturers in Canada differs significantly overall and by food category, with many products considered less healthy according to multiple nutrient profiling methods. Variation within food categories illustrates the need and potential for companies to improve the healthfulness of their products. Identifying companies that offer less healthy products compared with others in Canada may help prompt reformulation.

## Background

A food supply abundant in energy-dense products high in saturated fat, sodium and sugars contributes to poor diet quality and an elevated risk of obesity and other non-communicable diseases (NCDs) [1]. In Canada and elsewhere, food supplies are becoming increasingly dominated by packaged, pre-prepared and highly-processed food products [2–4]. The prevalence of diet-related chronic disease is high, with unhealthy diet being among the top risk factors for illness, disability and death in Canada and globally for over two decades [5, 6].

The national food supply is determined by the quality of foods and beverages provided by the domestic and multinational manufacturers that operate in the food system [1]. Food companies have been criticized for creating less healthy food supplies in recent years and are collectively considered a major driver of the NCD epidemic [7, 8]. The replacement of unprocessed or minimally-processed, traditional foods with highly-processed, energy-dense and nutrient-poor products manufactured by major food companies is of particular concern [7, 9, 10].

The World Health Organization (WHO) and others have acknowledged that efforts from food companies will be critical in preventing and controlling NCDs [11–13]. Specifically, the WHO has recommended that food companies eliminate *trans* fat and reduce saturated fat, free sugars and sodium in their existing products, and introduce new products with better nutritional value [14]. Given the limited scope and voluntary nature of most government policies and programs concerning product reformulation [15], voluntary action from food companies will be needed to improve the healthfulness of products available in Canada and other countries. While several food companies in Canada have committed to offer healthier products [16], it is unclear whether these voluntary policies actually translate into healthier product portfolios. There is therefore a need to benchmark food companies’ progress in offering healthier packaged foods and beverages, holding individual companies directly accountable for the nutritional quality of their products [1, 17].

Several reports have examined the nutritional quality of products offered by different food companies. The Access to Nutrition Initiative has released a global report on the healthfulness of products offered by multinational companies in nine national markets (Australia, China, Hong Kong, India, Mexico, New Zealand, South Africa, UK, USA) [18], as well as reports specific to companies and products in the UK, USA and India [19–21]. Studies have also examined the healthfulness of products offered by top food companies in Australia and New Zealand [22, 23]. All of these assessments found substantial variation in the nutritional quality of products offered by different companies, and many of their products were considered less healthy according to the Health Star Rating (HSR) system, which uses a nutrient profiling (NP) algorithm to rate the overall nutritional quality of packaged foods and beverages [18–23].

Although research has evaluated amounts of specific nutrients or NP scores of products in Canada’s overall food supply [24–28], no studies to our knowledge have examined or compared the nutritional quality of Canadian products at the level of individual food companies. The Canadian packaged food and beverage manufacturing sectors are comprised of a mixture of ‘Big Food’ multinational corporations, national manufacturers and Canadian retailers with private-label products [29, 30], but it is not known how these companies compare in terms of the healthfulness of their products. The purpose of this study was to compare the nutritional quality of products offered by the top packaged food and beverage companies in Canada. It was hypothesized that significant differences would be observed between companies in terms of the healthfulness of their products, overall and within most food categories.

## Methods

### Overview of methods

The top packaged food and beverage companies in Canada were identified based on sales data. Nutritional information for products offered by these companies in Canada was sourced from a branded food database. The nutritional quality of the sampled products was evaluated according to three metrics: 1) the HSR system; 2) amounts of calories, sodium, saturated fat and total sugars per 100 g (or mL) and per reference amounts (RAs) established by Health Canada; and 3) “high in” front-of-package (FOP) labelling thresholds for sodium, saturated fat and total sugars developed by Health Canada.

### Selection of companies

Companies holding ≥1% of the Canadian market share for packaged food and/or beverage sales as of 2018 (according to the Euromonitor International database) were selected, representing a combined 50.6 and 72.9% of the market shares for packaged food and beverages, respectively [29, 30]. Where both a subsidiary and its parent company held top market shares (e.g., Cadbury and Mondelēz), analyses were conducted at the parent company level (e.g., Mondelēz). Companies offering only bottled water were excluded (*n* = 2). Of the 22 sampled companies, 12 were multinational companies headquartered outside of Canada, 8 were Canadian companies or subsidiaries, and 2 were national retailers with private-label brands (Supplementary Table 1).

### Food composition data

Nutritional information for products was sourced from the University of Toronto Food Label Information Program (FLIP) 2017, described in detail elsewhere [27, 31]. Briefly, FLIP 2017 is a database of packaged food and beverage product labels collected from an outlet of each of three major Canadian grocery chains (Loblaws, Metro and Sobeys) in the Greater Toronto Area from June–September 2017. FLIP includes a product’s name, brand, company, Nutrition Facts table (NFt) and ingredients list, among other information. Products were classified according to the major (*n* = 24) and minor (*n* = 153) food categories in Health Canada’s Table of Reference Amounts for Food (TRA) [32]. NFt values were calculated per either 100 g or 100 mL for consistency within a TRA food category. Nutritional information for products was also calculated “as prepared” (i.e., accounting for added water and/or other ingredients), where applicable, based on instructions on the product’s packaging. Companies listed on product packages were searched online to verify whether they operated under any of the sampled companies as of 2017.

### Assessment of the nutritional quality of products

#### Health Star Rating system

The nutritional quality of products offered by companies was primarily assessed and ranked based on the HSR system, an interpretive FOP labelling system designed to help consumers select heathier foods [33, 34]. The underlying NP model is based on the Nutrient Profiling Scoring Criterion (NPSC), developed by Food Standards Australia New Zealand for regulating food health claims in Australia and New Zealand. The NPSC has been externally validated and applied globally [35]. The HSR system has adapted the NPSC to include three additional categories to better differentiate between dairy and non-dairy foods and beverages. Minor adjustments were also made to the NPSC thresholds for evaluating the energy, nutrient and fruit, vegetables, nuts and legumes (FVNL) content of non-dairy products in the HSR system. The HSR system has been applied in previous foreign assessments of the healthfulness of products offered by individual companies [18–23], facilitating comparisons of multinational companies’ products between countries.

All FLIP 2017 products were assigned to one of six HSR categories: 1) Non-dairy beverages; 1D) Dairy beverages; 2) Non-dairy foods; 2D) Dairy foods other than those in Category 1D or 3D (includes yogurts and cheeses with calcium content ≤320 mg/100 g and mixed foods with ≤25% other non-dairy ingredients); 3) Oils and spreads (includes margarine or butter); and 3D) Cheese and processed cheese (with calcium content > 320 mg/100 g). HSRs were determined based on nutritional composition per 100 g or mL, depending on the unit for which the NFt was displayed (in accordance with HSR calculation guidelines [36]). “Baseline points” were assigned for a product’s energy, saturated fat, sodium and total sugars content. “Modifying points” were awarded by estimating the proportion of the food consisting of FVNL ingredients. In the absence of quantitative ingredient declarations on Canadian products, a method to estimate the FVNL content of products using the ingredients list was developed and applied by our group, as described by Bernstein et al. [37]. Product categorization and FVNL point estimations were completed independently by three researchers, with discrepancies resolved through discussion with a fourth researcher. Some products were eligible to receive additional modifying points for dietary fibre or protein content. A final score was calculated by subtracting modifying points from baseline points, and an HSR (ranging from 0.5 to 5 stars in 0.5-star increments) was assigned based on predetermined thresholds, which vary by HSR category. Products with an HSR ≥3.5 were considered ‘healthy’, based on work by the New South Wales Ministry of Health in Australia which found that most foods with an HSR ≥3.5 aligned with Australian dietary guidelines [38].

#### Individual nutrients or components of public health concern

The calorie, sodium, saturated fat and total sugars contents of products were examined per 100 g or mL, and the RA, or the quantity of a type of food typically consumed by an individual in one sitting for the relevant TRA minor food category, as defined by Health Canada [32]. These particular nutrients and components have been associated with poor health outcomes and prioritized in public health initiatives of the WHO, Health Canada and other organizations [39, 40]. Nutrient amounts were evaluated independently of HSR scores, as the HSR system has been criticized for overestimating the nutritional quality of certain types of products (particularly sauces/dressings, spreads/dips, savoury snacks, meats, convenience foods, fruits and sweetened yogurts) [33, 41]. Nutrients were examined per both 100 g (or mL) and RA as the nutritional quality of certain types of foods may vary depending on whether it is examined per 100 g (or mL) or serving size (e.g., foods typically consumed in small amounts).

#### Health Canada’s FOP nutrition labelling thresholds

Products were also assessed against thresholds for sodium, saturated fat and total sugars that were proposed as part of pending federal FOP nutrition labelling regulations in Canada. In 2018, Health Canada published proposed regulations (in Canada Gazette, Part I) that would require foods containing ≥15% of the Daily Value (DV) for saturated fat, sodium and/or total sugars (≥30% DV for prepackaged meals and main dishes) to display “high in” FOP nutrition symbols [40]. Certain foods would be exempt from displaying a FOP nutrition symbol irrespective of their nutrient profile (e.g., fresh/frozen/canned fruits and vegetables without added ingredients except water, unsweetened/unflavoured animal milks, eggs, salt, sugar). In December 2019, the Prime Minister reiterated the federal government’s intention to introduce FOP nutrition labelling in Canada in a mandate letter to the Minister of Health [34], although as of February 2020, these regulations have not been finalized. To ensure the policy relevance of this study, the %DV thresholds for sodium, saturated fat and/or sugars were applied to all eligible products (i.e., all products that did not qualify for an exemption), as described in Canada Gazette, Part I [40].

### Products included in the sample

Products in FLIP 2017 offered by companies other than those in the study sample were excluded (*n* = 9200). Infant and toddler foods (*n* = 134), meal replacements and nutritional supplements (*n* = 36), non-alcoholic drink mixers (*n* = 14) and sugar substitutes (*n* = 7) offered by the sampled companies were excluded as these products are either not required to display the nutrition information required to calculate an HSR, or lack a specific RA. Three products without fibre information in their NFt were also excluded. Products available in multiple package sizes with identical nutritional composition (based on the NFt and ingredients list) were analyzed only once; however, all flavours or varieties of a product were included. The final sample included 8277 unique products.

### Statistical analyses

HSRs and calorie, sodium, saturated fat and total sugars contents of each company’s products were examined overall (i.e., their total products in FLIP 2017) and by TRA food category. For TRA major food categories encompassing a larger and broader range of minor categories, similar minor categories were combined. For example, the “bakery products” major food category consists of 25 minor categories, which were condensed into 4 categories: 1) “bread”; 2) “baked goods” (e.g., muffins, cakes, doughnuts); 3) “crackers, croutons and rice cakes”; 4) “grain-based, protein and energy bars”. TRA food categories with a narrower range of product types were presented at the major category level (e.g., desserts). Condensed soups, combination dishes requiring preparation (e.g., boxed macaroni and cheese) and beverage, baking, dessert, sauce or gravy powders and mixes were evaluated based on their nutritional value “as prepared” to facilitate comparisons with prepared products within that same food category; all other products were assessed on their nutritional composition “as sold”. Descriptive statistics examined the distribution of HSRs and nutrient amounts (per 100 g or mL and RA) of products offered by each company. Kruskal-Wallis tests examined differences in the mean ranks of HSRs for products offered by different companies, overall and by TRA major food category (statistically significant if *P* < 0.05). Companies with < 5 products in a food category were excluded from the Kruskal-Wallis test for that category. The proportion of products offered by each company with an HSR ≥3.5 and the number and proportion of companies’ products exceeding the “high in” threshold(s) for sodium, saturated fat and/or total sugars was calculated overall and by TRA major food category. Exempt products were included in (the denominators of) this analysis to avoid overestimating the proportion of products that would bear FOP nutrition symbols. Analyses were conducted in RStudio (version 1.2.1335; RStudio, Inc.).

## Results

### HSRs of companies’ products

**Fig. 1:**
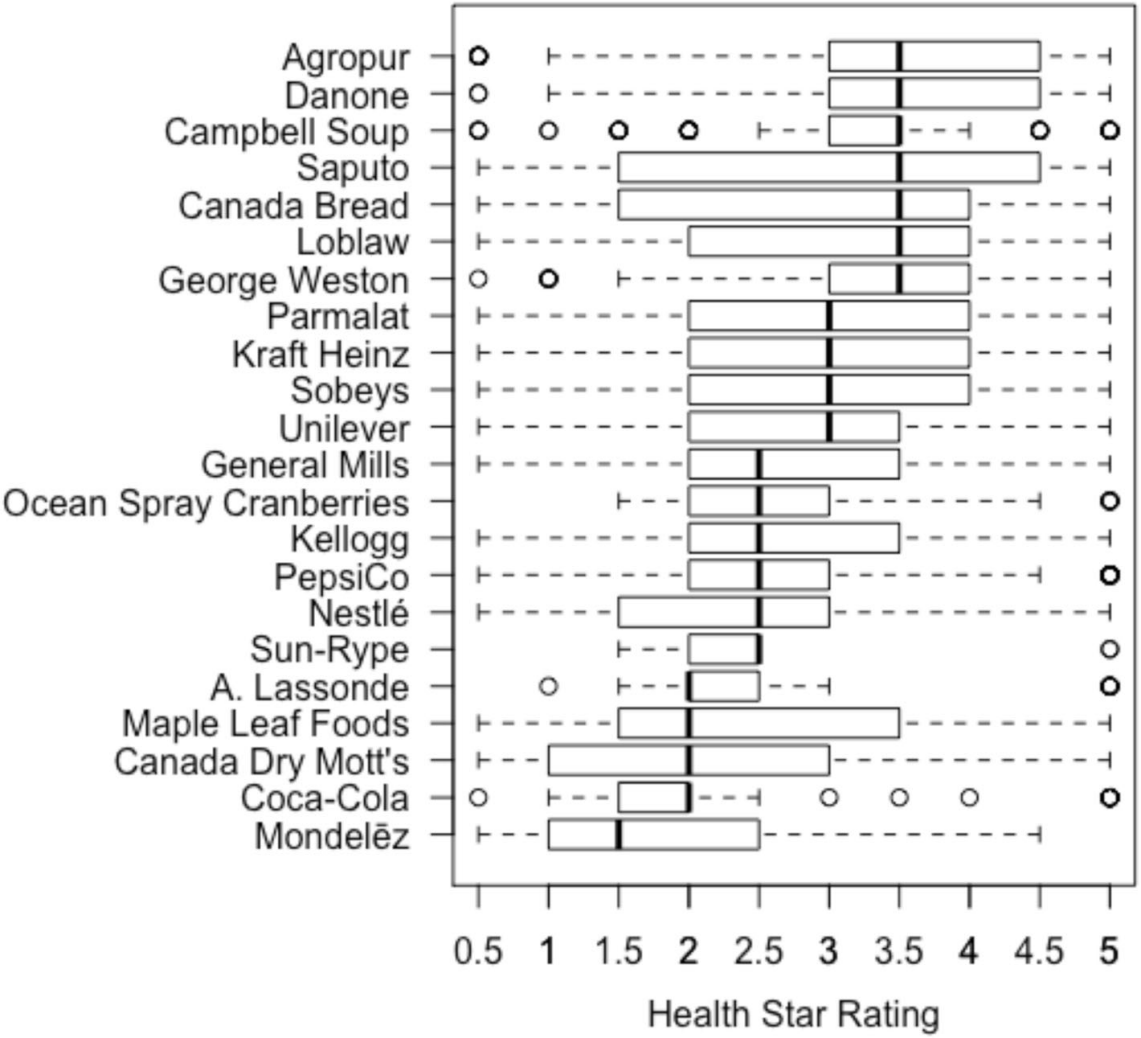
The distribution of Health Star Ratings of products offered by the major packaged food and beverage companies in Canada (*n* = 22). Bars represent the interquartile range, thick dark lines show the median, dotted lines show the non-outlier range, and circles indicate outliers

Mean HSRs of companies’ overall product offerings ranged from 1.9 to 3.6 (Tables 1 and 2). The distribution of each company’s HSR scores is presented in Fig. 1. Overall, 20 of the 22 companies had mean HSRs below 3.5 (Table 1; Fig. 1). On average, products offered by Agropur (x̅=3.6) were healthiest, while Mondelēz products were least healthy (x̅=1.9). Agropur also had the highest proportion of products with an HSR ≥3.5 (67.5%), while Sun-Rype had the fewest (5.6%, Table 1). HSRs of products differed significantly between companies overall and for all food categories (*P* < 0.05), except legumes (*P* = 0.57) and marine and freshwater animals (*P* = 0.076; Table 2). The greatest variation between companies was observed for fats and oils (mean HSR range of 0.7–5.0) and beverages (mean HSR range of 1.3–5.0; Table 2). Mean (± standard deviation) HSRs of companies’ products are presented by food category in Supplementary Table 2.

**Table 1:** Mean Health Star Rating (HSR) of the total products offered by each company in Canada, and proportion of each company’s product offerings with an HSR ≥3.5. A summary of the types of products offered by each company is also presented. Companies are arranged in descending order of mean HSR

Company	Products (n)	Mean HSR (SD)	HSR ≥ 3.5 (%)	Types of products offered
Agropur	123	3.6 (1.3)	67.5	Dairy
Danone	132	3.5 (1.0)	53.8	Beverages; dairy; frozen desserts
Campbell Soup	222	3.3 (0.8)	63.5	Beverages; condiments; snacks; soups
George Weston	169	3.2 (0.9)	62.7	Bread and bakery products
Saputo	95	3.1 (1.6)	54.7	Dairy
Loblaw	3099	3.1 (1.3)	51.4	Variety
Parmalat	120	3.0 (1.4)	44.2	Dairy
Canada Bread	96	3.0 (1.3)	65.6	Bread and bakery products
Sobeys	1633	2.9 (1.2)	46.2	Variety
Ocean Spray Cranberries	38	2.9 (1.2)	21.1	Beverages; dried cranberries; cranberry sauce
Kraft Heinz	460	2.9 (1.3)	45.7	Beverages; dairy; ready meals; snacks
General Mills	382	2.8 (1.3)	38.0	Breakfast cereals; ready meals; snacks; yogurt
Kellogg	138	2.8 (1.1)	33.3	Breakfast cereals; cereal bars; snacks
Unilever	217	2.8 (0.8)	29.5	Beverages; condiments; frozen desserts
PepsiCo	340	2.6 (1.1)	20.6	Beverages; breakfast cereals and bars; snacks
Nestlé	313	2.4 (1.1)	21.7	Beverages; confectionary; ready meals
Sun-Rype	36	2.4 (0.7)	5.6	Beverages; snacks
Maple Leaf Foods	158	2.3 (1.1)	31.0	Meat and meat products
A. Lassonde	77	2.3 (0.9)	9.1	Beverages
Coca-Cola	138	2.1 (1.2)	15.2	Beverages
Canada Dry Mott’s	64	2.1 (1.2)	23.4	Beverages
Mondelēz	227	1.9 (1.1)	15.0	Confectionary; snack foods

**Table 2:** The range in mean Health Star Ratings (HSRs) of companies’ products and the results of Kruskal-Wallis tests to compare the HSRs of products offered by different companies, overall and by food category^*a*^

Food category	Products (***n***)	Companies (***n***)	Minimum^**b**^	Maximum^**c**^	***P***-value^**d**^
OVERALL	8277	22	1.9	3.6	< 0.001
Bakery products	1459	12	1.0	3.3	< 0.001
Beverages	469	13	1.3	5.0	< 0.001
Cereals and other grain products	454	9	2.0	4.5	< 0.001
Combination dishes	619	9	2.0	4.1	< 0.001
Dairy products and substitutes	989	11	1.8	4.5	< 0.001
Dessert toppings and fillings	26	4	0.6	2.2	0.004^*d*^
Desserts	404	7	2.0	3.2	< 0.001
Eggs and egg substitutes	16	2	3.9	4.0	N/A^*d*^
Fats and oils	331	9	0.7	5.0	< 0.001
Fruit and fruit juices	582	10	1.4	3.5	< 0.001
Legumes	79	3	4.5	4.9	0.57^*d*^
Marine and fresh water animals	135	2	3.8	3.9	0.076
Meat, poultry, their products and substitutes	484	3	2.3	2.9	< 0.001
Miscellaneous category	215	12	0.5	2.9	< 0.001
Nuts and seeds	83	3	3.1	4.5	0.002
Potatoes, sweet potatoes and yams	53	3	3.5	4.1	0.016
Salads	61	2	3.7	4.1	< 0.001
Sauces, dips, gravies and condiments	497	11	1.0	3.4	< 0.001
Snacks	376	10	1.1	3.0	< 0.001
Soups	293	4	3.2	3.4	< 0.001
Sugars and sweets	315	9	0.7	2.5	< 0.001
Vegetables	337	8	1.5	4.4	< 0.001

^a^Food categories are defined in Health Canada’s Table of Reference Amounts for Food [32]

^b^The minimum mean Health Star Rating of a company’s products, overall or within a food category

^c^The maximum mean Health Star Rating of a company’s products, overall or within a food category

^d^P-value based on Kruskal-Wallis test that excluded companies with < 5 products in that food category

### Individual nutrients and components in companies’ products

Median (± interquartile range) amounts of calories, sodium, saturated fat and total sugars in companies’ products are presented in Tables 3 and 4, and Supplementary Table 3. Average nutrient/component amounts varied greatly by company and depending on whether they were evaluated per 100 g (or mL) or per RA. Results are reported separately for companies with packaged food portfolios (only or in addition to beverages; *n* = 18) versus beverage portfolios (*n* = 4) to help account for inter-company differences reflecting the nature of their product offerings. Companies with beverage portfolios included: A. Lassonde; Canada Dry Mott’s; Coca-Cola; and Ocean Spray Cranberries.

**Table 3:** Median amounts of calories, sodium, saturated fat and total sugars in companies’ Canadian product portfolios, presented per 100 g (or 100 mL) and per reference amount (RA)^*a*^

	Nutrient composition (median (interquartile range))								
		Calories (kcal)		Sodium (mg)		Saturated fat (g)		Total sugars (g)	
	Products (n)	100 g/mL	RA^**a**^	100 g/mL	RA^**a**^	100 g/mL	RA^**a**^	100 g/mL	RA^**a**^
**Packaged food**^***b***^									
Agropur	123	88 (269)	110 (50)	49 (589)	95 (111)	2 (16)	3 (4)	4 (10)	8 (18)
Campbell Soup	222	52 (47)	120 (90)	280 (148)	551 (526)	0 (1)	1 (2)	2 (5)	4 (7)
Canada Bread	96	267 (147)	197 (80)	380 (107)	279 (71)	1 (4)	1 (3)	3 (3)	2 (3)
Danone	132	100 (23)	162 (68)	54 (26)	95 (50)	1 (1)	2 (1)	11 (5)	18 (9)
General Mills	382	286 (290)	144 (85)	276 (406)	104 (131)	2 (3)	2 (3)	11 (22)	10 (12)
George Weston	169	280 (53)	183 (67)	455 (200)	293 (123)	1 (1)	1 (0)	4 (5)	2 (4)
Kellogg	138	397 (71)	181 (97)	400 (298)	150 (134)	2 (4)	1 (2)	19 (18)	9 (8)
Kraft Heinz	460	183 (255)	80 (75)	533 (614)	219 (248)	1 (7)	1 (3)	6 (11)	2 (7)
Loblaw	3099	233 (277)	128 (147)	260 (436)	135 (315)	1 (5)	1 (3)	3 (12)	3 (10)
Maple Leaf Foods	158	230 (179)	147 (165)	807 (326)	471 (226)	4 (8)	3 (5)	1 (3)	1 (2)
Mondelēz	227	467 (71)	140 (55)	310 (324)	93 (64)	4 (9)	1 (3)	25 (36)	8 (11)
Nestlé	313	150 (153)	212 (236)	69 (194)	88 (508)	3 (5)	4 (5)	11 (16)	16 (18)
Parmalat	120	257 (300)	120 (85)	338 (631)	105 (124)	10 (19)	4 (4)	3 (11)	2 (20)
PepsiCo	340	382 (448)	153 (171)	379 (584)	145 (203)	2 (3)	0 (2)	5 (13)	4 (13)
Saputo	95	233 (269)	110 (69)	200 (650)	169 (100)	8 (15)	3 (4)	5 (7)	1 (12)
Sobeys	1633	233 (287)	140 (163)	284 (460)	180 (319)	1 (5)	1 (4)	4 (14)	3 (12)
Sun-Rype	36	51 (280)	71 (55)	27 (28)	20 (18)	0 (0)	0 (0)	12 (68)	16 (13)
Unilever	217	103 (221)	80 (176)	237 (368)	128 (592)	0 (5)	1 (3)	2 (13)	2 (15)
**Beverages only**^***c***^									
A. Lassonde	77	44 (16)	110 (38)	6 (4)	15 (10)	0 (0)	0 (0)	10 (4)	25 (10)
Canada Dry Mott’s	64	43 (27)	60 (88)	15 (29)	50 (80)	0 (0)	0 (0)	10 (7)	12 (24)
Coca-Cola	138	40 (27)	115 (44)	8 (9)	20 (37)	0 (0)	0 (0)	10 (6)	25 (11)
Ocean Spray Cranberries	38	44 (40)	110 (88)	10 (10)	20 (29)	0 (0)	0 (0)	11 (9)	27 (18)

^a^RA: reference amount (defined in Health Canada’s Table of Reference Amounts for Food [32])

^b^Company offers packaged foods only or in addition to beverages

^c^Company offers beverages only

**Table 4:** The range in median amounts of calories, sodium, saturated fat and total sugars in companies’ products per 100 g (or mL) and reference amount (RA)^*a*^, overall and by food category^*a*^

Food category	Median calories (kcal)				Median sodium (mg)				Median saturated fat (g)				Median total sugars (g)			
	Per 100 g/mL		Per RA^***a***^		Per 100 g/mL		Per RA^***a***^		Per 100 g/mL		Per RA^***a***^		Per 100 g/mL		Per RA^***a***^	
	Min^***b***^	Max^***c***^	Min^***b***^	Max^***c***^	Min^***b***^	Max^***c***^	Min^***b***^	Max^***c***^	Min^***b***^	Max^***c***^	Min^***b***^	Max^***c***^	Min^***b***^	Max^***c***^	Min^***b***^	Max^***c***^
OVERALL	40	467	60	212	6	807	15	551	0	10	0	4	1	25	1	27
Bakery products	267	500	35	205	232	643	45	293	1	9	1	2	0	23	0	10
Beverages	0	37	0	137	0	22	0	83	0	0	0	0	0	10	0	36
Cereals and other grain products	356	436	110	367	12	1117	6	1117	0	3	0	2	0	28	0	12
Combination dishes	102	299	307	584	239	994	718	1987	0	7	1	13	2	5	4	13
Dairy products and substitutes	48	333	35	161	44	700	5	210	0	17	0	5	0	14	0	19
Dessert toppings and fillings	223	424	104	149	77	212	23	74	0	13	0	4	32	62	16	20
Desserts	55	229	71	297	38	109	50	142	0	11	0	14	13	18	17	23
Eggs and egg substitutes	132	132	132	132	129	129	129	129	3	3	3	3	0	0	0	0
Fats and oils	167	900	50	90	0	833	0	240	0	50	0	5	0	7	0	2
Fruit and fruit juices	39	52	59	130	0	10	0	25	0	0	0	0	9	12	12	29
Legumes	84	175	103	149	8	434	4	369	0	1	0	1	0	1	1	1
Marine and fresh water animals	106	108	100	102	347	358	323	340	0	1	0	1	0	0	0	0
Meat, poultry, their products and substitutes	200	214	127	165	471	829	413	463	3	4	2	3	0	0	0	0
Miscellaneous category	100	438	1	111	338	27,500	48	577	0	13	0	1	0	32	0	7
Nuts and seeds	600	667	90	198	0	400	0	60	6	7	1	2	4	7	1	1
Potatoes, sweet potatoes and yams	97	101	110	136	88	316	148	442	0	1	0	1	1	1	1	2
Salads	90	128	90	130	128	255	128	289	2	2	2	2	2	3	2	3
Sauces, dips, gravies and condiments	40	300	15	120	17	1733	10	372	0	9	0	3	0	40	0	24
Snacks	300	571	60	286	167	1217	83	609	2	10	1	5	1	33	0	17
Soups	8	48	20	120	244	293	610	733	0	0	0	1	0	1	1	3
Sugars and sweets	300	524	50	213	7	196	1	78	0	16	0	6	43	78	11	32
Vegetables	20	35	10	60	72	733	100	620	0	0	0	0	2	7	2	11

^a^Reference amounts (RA) and food categories are defined in Health Canada’s Table of Reference Amounts for Food [32]

^a^The minimum median amount of calories, sodium, saturated fat or total sugars in a company’s products, overall or within a food category

^b^The maximum median amount of calories, sodium, saturated fat or total sugars in a company’s products, overall or within a food category

#### Calories

Among packaged food companies, calories per 100 g (or mL) were highest for Mondelēz (median = 467 kcal) and lowest for Sun-Rype (median = 51 kcal; Table 3). For beverage companies, calories per 100 g (or mL) were highest in A. Lassonde and Ocean Spray Cranberries products (median = 44 kcal for both) and lowest in those of Coca-Cola (median = 40 kcal). When examined per RA, calories in products offered by packaged food companies were highest for Nestlé (median = 212 kcal) and lowest for Sun-Rype (median = 71 kcal). Among beverage companies, calories per RA were highest for Coca-Cola (median = 115 kcal) and lowest for Canada Dry Mott’s (median = 60 kcal). The greatest variation in the median calorie content of products offered by different packaged food and beverage companies per 100 g (or mL) was observed for fats and oils (range = 733 kcal), while combination dishes had the greatest differences between companies in median calorie content when examined per RA (range = 277 kcal; Table 4; Supplementary Table 3).

#### Sodium

For packaged food companies, sodium amounts per 100 g (or mL) were greatest in products offered by Maple Leaf Foods (median = 807 mg) and lowest in those of Sun-Rype (median = 27 mg) products. Among beverage companies, sodium per 100 g (or mL) was highest for Canada Dry Mott’s (median = 15 mg) and lowest for A. Lassonde (median = 6 mg). Sodium per RA in packaged food companies’ products was highest for Campbell Soup (median = 551 mg) and lowest for Sun-Rype (median = 20 mg). For beverage companies, sodium per RA was highest for Canada Dry Mott’s (median = 50 mg) and lowest for A. Lassonde (median = 15 mg). Differences in median amounts between companies were greatest for miscellaneous products (e.g., baking powder, batter mixes, spices and herbs) when assessed per 100 g or mL (range = 27,162 mg) and for combination dishes per RA (range = 1269 mg).

#### Saturated fat

Among packaged food companies, saturated fat per 100 g (or mL) was highest in products offered by Parmalat (median = 10 g), and lowest for the products of Campbell Soup, Sun-Rype and Unilever (median = 0 g for all). Similarly, saturated fat per RA was highest for Nestlé and Parmalat products (median = 4 g for both), and lowest for products offered by PepsiCo and Sun-Rype (median = 0 g for both). All 4 beverage companies (A. Lassonde, Canada Dry Mott’s, Coca-Cola, Ocean Spray Cranberries) had median saturated fat contents of 0 g per 100 g (or mL) and RA. Differences in the median saturated fat content of products offered by different companies were largest for fats and oils per 100 g or mL (range = 50 g), and for desserts when evaluated per RA (range = 14 g).

#### Total sugars

Total sugars per 100 g (or mL) among packaged food companies were greatest in products manufactured by Mondelēz (median = 25 g) and lowest for those made by Maple Leaf Foods (median = 1 g). Among the companies with beverages portfolios, total sugars per 100 g (or mL) were highest for Ocean Spray Cranberries (median = 11 g) and the same for the other companies (median = 10 g for A. Lassonde, Canada Dry Mott’s, Coca-Cola). When examined per RA, total sugars were highest for products offered by Danone (median = 18 g) and lowest for Maple Leaf Foods and Saputo (median = 1 g for both). For beverage companies, total sugars per RA were highest for Ocean Spray Cranberries (median = 27 g) and lowest for Canada Dry Mott’s (median = 12 g). Median total sugars contents of products differed the most between companies per 100 g (or mL) for sauces, dips, gravies and condiments (range = 40 g), and for beverages when examined per RA (range = 36 g).

### Health Canada’s proposed FOP labelling thresholds

The number and proportion of products offered by each company that exceeded Health Canada’s “high in” nutrient thresholds (and would therefore be required bear a FOP nutrition symbol under the proposed regulations) for sodium, saturated fat and/or total sugars are presented for companies’ overall product portfolios in Table 5, with a breakdown by food category in Supplementary Table 4. Overall, 66.4% of products would be required to bear ≥1 FOP nutrition symbol for sodium (31.7%), saturated fat (28.3%) and/or total sugars (28.4%, Table 5). Maple Leaf Foods had the greatest proportion of products exceeding one or more thresholds (97.5%), while Canada Bread offered the fewest (38.5%). Maple Leaf Foods had the largest proportion of products exceeding the %DV threshold for sodium (90.5%), Parmalat had the largest proportion exceeding the saturated fat threshold (68.3%) and Sun-Rype had the most products exceeding the total sugars threshold (86.1%). Within most food categories, there was considerable variation between companies in the proportion of their products that exceeded the %DV thresholds (Supplementary Table 4).

**Table 5:** The number and proportion of products exceeding Health Canada’s “high in” nutrient thresholds (≥15% Daily Value) for proposed front-of-pack (FOP) labelling regulations and would therefore be required to bear ≥1 FOP symbols for sodium, saturated fat and/or total sugars

Company	Total products ***n***^***a***^	Eligible products ***n***^***b***^	≥1 FOP symbol		Sodium		Saturated fat		Total sugars	
			***n***	%^***c***^	***n***	%^***c***^	***n***	%^***c***^	***n***	%^***c***^
A. Lassonde	77	77	61	79.2	1	1.3	0	0.0	60	77.9
Agropur	123	115	90	73.2	19	15.4	48	39.0	42	34.1
Campbell Soup	222	221	191	86.0	162	73.0	32	14.4	33	14.9
Canada Bread	96	96	37	38.5	13	13.5	25	26.0	17	17.7
Canada Dry Mott’s	64	62	39	60.9	9	14.1	0	0.0	30	46.9
Coca-Cola	138	138	113	81.9	9	6.5	1	0.7	108	78.3
Danone	132	132	103	78.0	0	0.0	35	26.5	86	65.2
General Mills	382	378	295	77.2	97	25.4	105	27.5	175	45.8
George Weston	169	169	95	56.2	79	46.7	12	7.1	13	7.7
Kellogg	138	138	84	60.9	26	18.8	32	23.2	37	26.8
Kraft Heinz	460	460	345	75.0	260	56.5	117	25.4	66	14.3
Loblaw	3099	2935	1867	60.2	941	30.4	866	27.9	694	22.4
Maple Leaf Foods	158	158	154	97.5	143	90.5	84	53.2	13	8.2
Mondelēz	227	227	142	62.6	31	13.7	83	36.6	105	46.3
Nestlé	313	309	249	79.6	78	24.9	168	53.7	163	52.1
Ocean Spray Cranberries	38	38	26	68.4	0	0.0	1	2.6	26	68.4
Parmalat	120	120	106	88.3	28	23.3	82	68.3	39	32.5
PepsiCo	340	337	166	48.8	67	19.7	33	9.7	94	27.6
Saputo	95	87	73	76.8	25	26.3	60	63.2	23	24.2
Sobeys	1633	1545	1073	65.7	543	33.3	508	31.1	435	26.6
Sun-Rype	36	36	31	86.1	0	0.0	0	0.0	31	86.1
Unilever	217	217	158	72.8	91	41.9	53	24.4	64	29.5
TOTAL	8277	7995	5498	66.4	2622	31.7	2345	28.3	2354	28.4

^a^Total number of products offered by the company - overall or within a given food category – irrespective of whether a product was exempt from Health Canada’s proposed requirement to display a FOP nutrition symbol

^b^Number of products offered by the company – overall or within a given food category – that would be eligible for evaluation against the proposed thresholds for displaying a FOP nutrition symbol (i.e., products that did not qualify for exemption)

^c^Denominator consists of all products offered by the company (including those that would be exempt from the requirement to display a FOP nutrition symbol)

## Discussion

This study provides the first comprehensive comparison of the nutritional quality of products offered by the top packaged food and beverage companies in Canada. Consistent with evaluations of the healthfulness of products offered by companies in other national markets [18–20, 22], this work found significant variation in the nutritional quality of products between major packaged food and beverage companies in Canada. This is perhaps unsurprising, given that companies manufacture a range of types of food and beverage products, but illustrates how different companies are contributing to a less healthy food environment in Canada.

These results demonstrate that the average nutritional quality of products offered by a food company is likely closely related to the nature of their product portfolio. Companies solely or primarily manufacturing dairy products (e.g., Agropur, Danone) typically had higher average HSRs, while companies with confectionary or soft drinks making up large proportions of their product portfolios (e.g., Mondelēz, Coca-Cola) scored lower, on average. These findings are consistent with a recent report on the nutritional quality of the Australian food supply [22], which found that dairy companies’ products often had higher HSRs than products from soft drink manufacturers or those with a large number of confectionary foods, which are often considerably energy-dense and offer little nutritional value. Similarly, amounts of individual nutrients in companies’ products also reflected differences according to companies’ product portfolios. For example, companies primarily offering packaged meats, soups and/or sauces had more sodium in their product portfolios, on average (e.g., Maple Leaf Foods, Campbell Soup). While the present study was intended to provide an overall summary and comparison of the healthfulness of products offered by the leading food companies in Canada, these results demonstrate the value of comparing the nutritional quality of similar products offered by the top companies within individual food categories in order to help inform and support product reformulation.

Overall, the majority of companies’ products were of poor nutritional quality, with mean HSRs of products offered by nearly all companies scoring below 3.5/5 and 66% of all products exceeding at least one of Health Canada’s “high in” thresholds for nutrients of public health concern. No company fared consistently better than others across all of the metrics assessed (i.e., in terms of the HSR, calories and negative nutrients per 100 g (or mL) and RA, or the %DV thresholds). These findings suggest that while some companies are offering healthier products in Canada, less healthy foods and beverages still constitute the majority of the portfolios of most major companies in this sample. Consequently, Canadian consumers must make food choices in an environment dominated by nutrient-poor foods and beverages. Given that these products are often heavily marketed using nutrition claims, child-appealing packaging and/or pricing strategies that favour less healthy products, the current food environment limits consumers’ ability to make healthier food choices [28, 42–44].

Many of the sampled companies have reported voluntary commitments or actions to reduce saturated fat, trans fat, sodium, added sugars and/or calories in their products [16]. The strength of companies’ commitments was evaluated using a globally-developed and -applied framework (the Business Impact Assessment – Obesity and Population-level Nutrition, BIA-Obesity) that provides an overall score based on the transparency, comprehensiveness, specificity and national-level applicability of a company’s commitments concerning product formulation (and other areas of the food environment) [45]. In Canada, the median score for the product formulation policy domain among all companies was 27/100, with Nestlé scoring the highest (89/100), followed by Unilever (71/100) and Mondelēz (58/71) [16]. Higher scores were awarded to companies with SMART (specific, measurable, attainable, realistic, time-sensitive) targets and actions concerning the reduction of multiple nutrients of concern (sodium, added or free sugars, saturated fat, trans fat, and portion sizes or energy content) where relevant across the company’s product portfolio, and that publicly report their progress on a regular basis. Although these companies reported specific commitments to reduce negative nutrients across their product portfolios, their products remained high in saturated fat, sodium and/or sugars as of 2017, and had low HSR scores. The juxtaposition of these findings suggests that stronger voluntary commitments do not necessarily result in company product portfolios of better nutritional quality. However, further analysis is warranted to assess how companies’ commitments compare to their actions regarding the healthfulness of their products.

Our results demonstrate considerable opportunity for the largest packaged food and beverage companies in Canada to improve the nutritional quality of their products, both overall and within food categories. The wide ranges in HSRs and amounts of calories, saturated fat, sodium and sugars of products within food categories indicate that offering healthier versions of most products is possible and may be feasible for manufacturers. There was substantial variation in product healthfulness between companies for nearly all food categories, except categories with products primarily consisting of whole foods containing few ingredients, with little variability in nutritional composition between products (e.g., eggs, legumes). Reformulating existing products to reduce their caloric density and saturated fat, sodium and sugar content, introducing healthier products within existing product lines, or acquiring healthier brands or product lines from other companies in exchange for current less healthy alternatives may provide opportunities for improvement.

This study was unique in its inclusion of two major Canadian retailers with private-label brands. In recent years, private-label brands in Canada have increasingly aligned with consumer trends and values, offering high-quality products at lower prices than national brand alternatives [46, 47]. As a result, grocery retailers with private-label products have become dominant in the Canadian market, with retailers constituting approximately 13% of packaged food sales and 20% of that for beverages as of 2018 [29, 30], following 5 years of consistent share growth of private-label products in Canada [47]. Compared with many multinational or domestic manufacturers, retailers typically manufacture a greater number and variety of products within their national markets [48], as evidenced in this study where Loblaw and Sobeys offered the most products overall and were the only companies with products in every major food category examined. Products offered by these two retailers were neither among the healthiest nor the least healthy overall. This finding is somewhat surprising given that, unlike most other sampled companies, Loblaw and Sobeys offer many unprocessed or minimally-processed packaged foods that align with Canada’s Dietary Guidelines [49] (and were assessed in this study), such as frozen plain fruits and vegetables, minimally-processed meats and seafood, and dry or canned legumes and grains. Our findings may suggest that while Canada’s top grocery retailers are making healthier private-label options available to consumers, a large proportion of their product portfolios are still of relatively poor nutritional quality.

While voluntary efforts from food companies to offer healthier products will be critical in improving the Canadian food supply, governments play a key role by establishing the regulatory environment in which companies operate [50]. In Canada, the federal government has recently introduced several initiatives with direct or indirect effects on the nutritional quality of the food supply, including voluntary targets for sodium reduction in processed foods established in 2012 [51], a ban on industrially-produced *trans* fat in foods as of 2018 [52], and proposed restrictions on unhealthy food marketing to children [53] and mandatory FOP nutrition symbols on foods high in saturated fat, sodium and/or total sugars [40]. This study found 20 of 22 major food companies in Canada would be required to label more than half of their products as high in one or more nutrients of concern. Additionally, 32% of products offered by the sampled companies were found to be high in sodium, and 10 of the 22 companies would be required to carry a FOP nutrition symbol for sodium on at least one-quarter of their products. These results provide further evidence that Health Canada’s voluntary sodium reduction targets have had limited uptake from industry [51], with considerable room for improvement remaining. Given the large proportion of products that exceed the thresholds established by Health Canada, the proposed FOP regulations, which include a transition period of approximately 4 years from their implementation [40], would allow time for manufacturers to either add the appropriate FOP nutrition symbols to their products or reformulate products such that they would no longer exceed the “high in” thresholds for sodium, saturated fat and/or sugars (and would not be required to bear a FOP nutrition symbol).

In the absence of a consensus on the superiority of a single approach to assessing the nutritional quality of a food, products in this study were evaluated according to both an NP model and in terms of individual nutrients of public health concern per 100 g (or mL) and RA. While the HSR system was valuable for assessing the nutritional composition of products based on both positive and negative nutrients and components, and for ranking companies by the average healthfulness of their products, this NP model has several limitations [41, 54, 55]. For example, the current HSR scoring algorithm enables some products that are rich in protein, fibre and/or FVNL – but also high in calories, saturated fat, sodium and/or sugars – to achieve relatively high HSRs. Examining amounts of individual negative nutrients/components helped account for this limitation, the importance of which was apparent in our observation that no company’s product portfolio was consistently considered healthy by all of the assessments conducted in this study. For example, although Agropur had the highest overall mean HSR, their products were found to be relatively high in saturated fat and sodium, and 73% exceeded at least one %DV threshold. Additionally, because the HSR only evaluates the nutritional composition of foods per 100 g or mL, foods with large RAs (or stated serving sizes) may receive high HSRs despite being rich in calories or negative nutrients. This was reflected in our results where ranking of companies based on average levels of calories, sodium, saturated fat and total sugars in their products differed depending on whether they were assessed per 100 g (or mL) or RA. The HSR system is currently undergoing a 5-year review following its voluntary implementation as a FOP labelling system in Australia and New Zealand in 2014, which may address some of the weaknesses identified in the system [56].

This study is strengthened by its analysis of a large, highly representative sample of packaged food and beverage companies whose products constitute a significant proportion of the Canadian food supply. Given that about two-thirds of these companies are multinational, our results may be somewhat generalizable to other food supplies, although there is evidence that nutrients levels of the same products from multinational food companies often vary by market [18]. Additionally, the study was conducted independently of interested stakeholders (i.e., food industry or government representatives, policymakers), a considerable strength. Limitations include that data collection was restricted to three retail outlets in one major city at a single time point and, as such, may not capture absolutely all products offered by the sampled companies in the Canadian food supply. This limitation could potentially be mitigated in future research if companies shared data on the nutritional composition of their products with researchers or publicly accessible websites to ensure that all products in Canada (or other markets of interest) were being assessed. In addition, some products in this sample may have been discontinued, reformulated or acquired by a different company since data were collected in 2017.

## Conclusions

There is considerable variation in the nutritional quality of products offered by the top packaged food and beverage companies in Canada, and many of their products are energy-dense and high in sodium, saturated fat and/or sugars. Findings from this study suggest a need and an opportunity for many companies to improve the nutritional quality of their products, such as through reformulation of existing products, the development of healthier alternatives or by acquiring healthier brands or product lines. By holding individual companies directly accountable for the healthfulness of their products, results from this work may prompt efforts from food companies and/or Health Canada to improve the nutritional quality of the Canadian food supply.

## Supplementary information

**Additional file 1 : Supplementary Table 1** A summary of the top 22 packaged food and beverage companies in Canada in terms of their market share, type (i.e., multinational or domestic manufacturer, retailer), area served and location of head office. **Supplementary Table 2**. Mean (± standard deviation) Health Star Ratings of products offered by each company, presented by food category. **Supplementary Table 3**. Median (± interquartile range) amounts of calories, sodium, saturated fat and total sugars per 100 g (or mL) and per reference amount in products offered by each company, presented by food category. **Supplementary Table 4**. The number and percentage of products offered by each company that exceeded Health Canada’s proposed “high in” front-of-package labelling thresholds for saturated fat, sodium and/or total sugars, presented by food category.

## Data Availability

The datasets generated and/or analysed during the current study are not publicly available but are available from the corresponding author on reasonable request.

## Abbreviations

ATNI: Access to Nutrition Initiative
BIA-Obesity: Business Impact Assessment – Obesity and population-level nutrition
DV: Daily Value
FLIP: Food Label Information Program
FOP: Front-of-package
FVNL: Fruits, vegetables, nuts and legumes
HSR: Health Star Rating
NCD: Non-communicable disease
NFt: Nutrition Facts table
NP: Nutrient profiling
NPSC: Nutrient Profiling Scoring Criterion
RA: Reference Amount
TRA: Table of Reference Amounts for Food
WHO: World Health Organization

## References

[1] Neal B, Sacks G, Swinburn B, Vandevijvere S, Dunford E, Snowdon W (2013). Monitoring the levels of important nutrients in the food supply. Obes Rev.

[2] Monteiro CA, Moubarac JC, Cannon G, Ng SW, Popkin B (2013). Ultra-processed products are becoming dominant in the global food system. Obes Rev.

[3] Moubarac JC, Batal M, Martins AP, Claro R, Levy RB, Cannon G (2014). Processed and ultra-processed food products: consumption trends in Canada from 1938 to 2011. Can J Diet Pract Res.

[4] Nardocci M, Polsky J, Moubarac J. How ultra-processed foods affect health in Canada. Report prepared for Heart and Stroke. Montréal: TRANSNUT, Department of Nutrition, University of Montreal; 2019.

[5] Statistics Canada (2017). Leading causes of death, by sex (both sexes).

[6] GBD 2017 Diet Collaborators (2019). Health effects of dietary risks in 195 countries, 1990–2017: a systematic analysis for the Global Burden of Disease Study 2017. Lancet.

[7] Stuckler D, Nestle M (2012). Big food, food systems, and global health. PLoS Med.

[8] Moodie R, Stuckler D, Monteiro C, Sheron N, Neal B, Thamarangsi T (2013). Profits and pandemics: prevention of harmful effects of tobacco, alcohol, and ultra-processed food and drink industries. Lancet..

[9] Monteiro CA, Cannon G, Moubarac JC, Levy RB, Louzada MLC, Jaime PC (2018). The UN decade of nutrition, the NOVA food classification and the trouble with ultra-processing. Public Health Nutr.

[10] Stuckler D, McKee M, Ebrahim S, Basu S (2012). Manufacturing epidemics: the role of global producers in increased consumption of unhealthy commodities including processed foods, alcohol, and tobacco. PLoS Med.

[11] World Health Organization (2013). Global Action Plan for the Prevention and Control of Non-Communicable Diseases 2013-2020.

[12] Swinburn BA, Sacks G, Hall KD, McPherson K, Finegood DT, Moodie ML (2011). The global obesity pandemic: shaped by global drivers and local environments. Lancet..

[13] Dr. Margaret Chan. Opening address at the 8th global conference on health promotion. Helsinki, Finland: World Health Organization; 2010. http://www.who.int/dg/speeches/2013/health_promotion_20130610/en/. Accessed 30 Oct 2019.

[14] World Health Organization. Global Strategy on Diet, Physical Activity and Health. Geneva; 2004. https://www.who.int/dietphysicalactivity/strategy/eb11344/strategy_english_web.pdf. Accessed 30 Oct 2019.

[15] World Cancer Research Fund International. NOURISHING database: Improve nutritional quality of the whole food supply; 2019. https://www.wcrf.org/int/policy/nourishing-database. Accessed 6 May 2020.

[16] Vanderlee L, Vergeer L, Sacks G, Robinson E, L'Abbé M. Food and beverage manufacturers in Canada: policies and commitments to improve the food environment: University of Toronto; 2019. http://labbelab.utoronto.ca/bia-obesity-canada-2019/. Accessed 30 Oct 2019.

[17] Sacks G, Swinburn B, Kraak V, Downs S, Walker C, Barquera S (2013). A proposed approach to monitor private-sector policies and practices related to food environments, obesity and non-communicable disease prevention. Obes Rev.

[18] Access to Nutrition Initiative. Global Index 2018. https://accesstonutrition.org/index/global-index-2018/. Accessed 6 May 2020.

[19] Access to Nutrition Initiative. U.S. Spotlight Index 2018. https://accesstonutrition.org/countries/us-spotlight-index/. Accessed 6 May 2020.

[20] Access to Nutrition Initiative. India Spotlight Index 2020. https://accesstonutrition.org/index/india-spotlight-2020/. Accessed 6 May 2020.

[21] Access to Nutrition Initiative. U.K. Product Profile 2019. https://accesstonutrition.org/countries/uk-product-profile-2019/. Accessed 6 May 2020.

[22] Neal B, Sacks G, Shahid M, Taylor F, Huffman M. FoodSwitch: State of the Food Supply (April 2019) 2019. https://www.georgeinstitute.org/sites/default/files/food_supply_report.pdf. Accessed 30 Oct 2019.

[23] Mackay S, Ni Mhurchu C, Swinburn B, Eyles H, Young L, Gontijo de Castro T (2019). State of the Food Supply: New Zealand 2019.

[24] Arcand J, Scourboutakos MJ, Au JT, L'Abbe MR (2014). Trans fatty acids in the Canadian food supply: an updated analysis. Am J Clin Nutr.

[25] Arcand J, Jefferson K, Schermel A, Shah F, Trang S, Kutlesa D (2016). Examination of food industry progress in reducing the sodium content of packaged foods in Canada: 2010 to 2013. Appl Physiol Nutr Metab.

[26] Scourboutakos MJ, L'Abbé MR (2014). Changes in sodium levels in chain restaurant foods in Canada (2010-2013): a longitudinal study. CMAJ Open.

[27] Bernstein JT, Schermel A, Mills CM, L'Abbé MR. Total and Free Sugar Content of Canadian Prepackaged Foods and Beverages. Nutrients. 2016;8(9):E582. 10.3390/nu8090582 PMC5037566 27657125

[28] Labonté M, Poon T, Mulligan C, Bernstein JT, Franco-Arellano B, L'Abbé MR. Comparison of global nutrient profiling systems for restricting the commercial marketing of foods and beverages of low nutritional quality to children in Canada. Am J Clin Nutr. 2017;106(6):1471–81. 10.3945/ajcn.117.161356 29070562

[29] Packaged food in Canada. Company Shares | National - Latest Owner | Historical | % breakdown. Euromonitor International. 2018. https://go.euromonitor.com/passport.html. Accessed 6 May 2020.

[30] Soft Drinks in Canada. Company Shares | National - Latest Owner | Historical | % breakdown. Euromonitor International. 2018. https://go.euromonitor.com/passport.html. Accessed 6 May 2020.

[31] Franco-Arellano B, Arcand J, Kim MA, Schermel A, L'Abbé M. Progress towards reducing industrially-produced trans-fatty acids in the Canadian marketplace, 2013–2017. Public Health Nutrition (In press; accepted 11 Nov 2019). 10.1017/S1368980019004816 PMC11377961 32482203

[32] Health Canada. Nutrition Labelling Table of Reference Amounts for Food. 2016. https://www.canada.ca/en/health-canada/services/technical-documents-labelling-requirements/table-reference-amounts-food.html. Accessed 30 Oct 2019.

[33] Jones A, Rådholm K, Neal B. Defining ‘Unhealthy’: A Systematic Analysis of Alignment between the Australian Dietary Guidelines and the Health Star Rating System. Nutrients. 2018;10(4):E501. 10.3390/nu10040501 PMC5946286 29670024

[34] Trudeau J. Minister of Health Mandate Letter: Government of Canada; 2019. https://pm.gc.ca/en/mandate-letters/2019/12/13/minister-health-mandate-letter. Accessed 14 Feb 2020.

[35] Poon T, Labonté M, Mulligan C, Ahmed M, Dickinson KM, L'Abbé MR (2018). Comparison of nutrient profiling models for assessing the nutritional quality of foods: a validation study. Br J Nutr.

[36] Guide for industry to the Health Star Rating Calculator (HSRC) 2018. http://healthstarrating.gov.au/internet/healthstarrating/publishing.nsf/Content/guide-for-industry-document. Accessed 6 May 2020.

[37] Bernstein JT, Franco-Arellano B, Schermel A, Labonté M, L'Abbé MR (2017). Healthfulness and nutritional composition of Canadian prepackaged foods with and without sugar claims. Appl Physiol Nutr Metab..

[38] Dunford E, Cobcroft M, Thomas M, Wu J. Technical Report: Alignment of the NSW Healthy Food Provision Policy with the Health Star Rating System Sydney, NSW: NSW Ministry of Health; 2015. http://www.health.nsw.gov.au/heal/Publications/health-star-rating-system.pdf. Accesesed 30 Oct 2019.

[39] World Health Organization. Healthy diet. 2018. https://www.who.int/news-room/fact-sheets/detail/healthy-diet. Accessed 30 Oct 2019.

[40] Government of Canada. Regulations Amending Certain Regulations Made Under the Food and Drugs Act (Nutrition Symbols, Other Labelling Provisions, Partially Hydrogenated Oils and Vitamin D). 2018. http://gazette.gc.ca/rp-pr/p1/2018/2018-02-10/html/reg2-eng.html. Accessed 30 Oct 2019.

[41] Lawrence M, Dickie S, Woods JL. Do Nutrient-Based Front-of-Pack Labelling Schemes Support or Undermine Food-Based Dietary Guideline Recommendations? Lessons from the Australian Health Star Rating System. Nutrients. 2018;10(1):E32. 10.3390/nu10010032 PMC5793260 29303956

[42] Franco-Arellano B, Labonté M, Bernstein JT, L'Abbé MR. Examining the Nutritional Quality of Canadian Packaged Foods and Beverages with and without Nutrition Claims. Nutrients. 2018;10(7):E832. 10.3390/nu10070832 PMC6073495 29954102

[43] Ricciuto L, Ip H, Tarasuk V (2005). The relationship between price, amounts of saturated and trans fats, and nutrient content claims on margarines and oils. Can J Diet Pract Res.

[44] Sumanac D, Mendelson R, Tarasuk V (2013). Marketing whole grain breads in Canada via food labels. Appetite..

[45] Sacks G, Vanderlee L, Robinson E, Vandevijvere S, Cameron AJ, Ni Mhurchu C, et al. BIA-Obesity (Business Impact Assessment — Obesity and population-level nutrition): A tool and process to assess food company policies and commitments related to obesity prevention and population nutrition at the national level. Obes Rev. 2019;20(Suppl 2):78–89. 10.1111/obr.12878 31317645

[46] The Nielsen Company. The Rise and Rise Again of Private Label. 2018. https://www.nielsen.com/wp-content/uploads/sites/3/2019/04/global-private-label-report.pdf. Accessed 30 Oct 2019.

[47] Harris R. The rising power of private label: Canadian Grocer. 2018. http://www.canadiangrocer.com/worth-reading/the-rising-power-of-private-label-81826. Accessed 30 Oct 2019.

[48] Euromonitor International. Grocery retailers in Canada. 2017. https://www.euromonitor.com/store. Accessed 6 May 2020.

[49] Health Canada. Canada's Dietary Guidelines. 2019. https://food-guide.canada.ca/en/guidelines/. Accessed 6 May 2020.

[50] Swinburn B, Sacks G, Vandevijvere S, Kumanyika S, Lobstein T, Neal B, et al. INFORMAS (International Network for Food and Obesity/non-communicable diseases Research, Monitoring and Action Support): overview and key principles. Obes Rev. 2013;14(Suppl 1):1–12. 10.1111/obr.12087 24074206

[51] Health Canada. Sodium Reduction in Processed Foods in Canada: An Evaluation of Progress toward Voluntary Targets from 2012 to 2016. 2018. https://www.canada.ca/en/health-canada/services/food-nutrition/legislation-guidelines/guidance-documents/guidance-food-industry-reducing-sodium-processed-foods-progress-report-2017.html. Accessed 30 Oct 2019.

[52] Canadian Food Inspection Agency (2019). Notice of modification: Prohibiting the use of partially hydrogenated oils (PHOs) in foods.

[53] Parliament of Canada. Bill S-228 An Act to amend the Food and Drugs Act (prohibiting food and beverage marketing directed at children). https://www.parl.ca/LegisInfo/BillDetails.aspx?billId=8439397&Language=E. Accessed 30 Oct 2019.

[54] Jones A, Thow AM, Ni Mhurchu C, Sacks G, Neal B. The performance and potential of the Australasian Health Star Rating system: a four-year review using the RE-AIM framework. Aust N Z J Public Health. 2019;43(4):355–65. 10.1111/1753-6405.12908 31141289

[55] Lawrence MA, Pollard CM, Vidgen HA, Woods JL. The Health Star Rating system - is its reductionist (nutrient) approach a benefit or risk for tackling dietary risk factors? Public Health Res Pract. 2019;29(1):2911906. 10.17061/phrp2911906 30972406

[56] Australian Government (2019). Formal review of the system after five years of implementation (June 2014 to June 2019).

[57] Vergeer L, Ahmed M, Franco-Arellano B, Dickinson K, Mulligan C, Vanderlee L, L'Abbé MR. An evaluation and comparison of the nutritional quality of packaged food and beverage products offered by major food companies in Canada according to the Health Star Rating system. Abstract Book for the ISBNPA 2019 Annual Meeting in Prague, p. 751. Published by: International Society of Behavioral Nutrition and Physical Activity on June 17, 2019. ISBN: 978-7324011-1-2. https://www.isbnpa.org/index.php?r=article/view&id=122. Accessed 16 Mar 2020.

[58] Vergeer L, Ahmed M, Vanderlee L, L'Abbé M (2019). Examining the nutritional quality of the product portfolios of major packaged food and beverage companies in Canada. Abstract published by the 13^th^ European Nutrition Conference (FENS 2019).

