# Supplementary material for: A comparison of the nutritional quality of products offered by the top packaged food and beverage companies in Canada
Source: BMC Public Health. 2020 May 11;20:650. doi: 10.1186/s12889-020-08828-w (PMC7216504; doi:10.1186/s12889-020-08828-w)
Supplement: Supplementary file 1 — Additional file 1 : Supplementary Table 1 A summary of the top 22 packaged food and beverage companies in Canada in terms of their market share, type (i.e., multinational or domestic manufacturer, retailer), area served and location of head office. Supplementary Table 2. Mean (± standard deviation) Health Star Ratings of products offered by each company, presented by food category. Supplementary Table 3. Median (± interquartile range) amounts of calories, sodium, saturated fat and total sugars per 100 g (or mL) and per reference amount in products offered by each company, presented by food category. Supplementary Table 4. The number and percentage of products offered by each company that exceeded Health Canada’s proposed “high in” front-of-package labelling thresholds for saturated fat, sodium and/or total sugars, presented by food category. [file 12889_2020_8828_MOESM1_ESM.pdf]

SUPPLEMENTARY MATERIAL

A comparison of the nutritional quality of products offered by the top packaged food and beverage companies in Canada

Laura Vergeer[1], Lana Vanderlee[1,2], Mavra Ahmed[1], Beatriz Franco-Arellano[1], Christine Mulligan[1], Kacie Dickinson[1,3], Mary R. L'Abbé[1]

[1]Department of Nutritional Sciences, Faculty of Medicine, University of Toronto

1 King's College Circle, Toronto, Ontario, Canada M5S 1A8

[2]School of Public Health and Health Systems, University of Waterloo

200 University Avenue West, Waterloo, Ontario, Canada N2L 3G1

[3]Nutrition and Dietetics, College of Nursing and Health Sciences, Flinders University

G.P.O. Box 2100, Adelaide, SA 5001, Australia

Table of Contents

Supplementary Table 1. A summary of the top 22 packaged food and beverage companies in Canada in terms of their market share, type (i.e., multinational or domestic manufacturer, retailer), area served and location of head office ……………………………………………...... 1

Supplementary Table 2. Mean (± standard deviation) Health Star Ratings of products offered by each company, presented by food category …..………………...……………………………. 3

Supplementary Table 3. Median (± interquartile range) amounts of calories, sodium, saturated fat and total sugars per 100 g (or mL) and per reference amount in products offered by each company, presented by food category ……..………………..…………………………………..…. 6

Supplementary Table 4. The number and percentage of products offered by each company that exceeded Health Canada's proposed "high in" front-of-package labelling thresholds for saturated fat, sodium and/or total sugars, presented by food category ……………………………..……… 19

Supplementary Table 1. A summary of the top 22 packaged food and beverage companies in Canada in terms of their market share, type (i.e., multinational or domestic manufacturer, retailer), area served and location of head office.

Sector	Company[1]	Market share (%)[1]	Type (area served)	Location of head office
Packaged food	Loblaw Companies Ltd.	6.3	Retailer (Canada)	Canada
	Saputo Inc.	5.1	Multinational (Global)	Canada
	Kraft Heinz Canada ULC	4.5	Multinational (Global)	USA
	Nestlé Canada Inc.	4.0	Multinational (Global)	Switzerland
	Agropur Cooperative Ltd.	3.9	Multinational (North America)	Canada
	Frito-Lay Canada (PepsiCo Canada ULC)	3.8	Multinational (Global)	USA
	Parmalat Canada Ltd.	3.3	Multinational (Global)	Italy
	Schneider Corp./Maple Leaf Foods Inc.[2]	3.1	Domestic (Canada)	Canada
	Cadbury Adams Canada Inc./Mondelēz Canada Inc.[2]	2.6	Multinational (Global)	USA
	General Mills Canada Corp.	2.5	Multinational (Global)	USA
	Sobeys Inc.	2.3	Retailer (Canada)	Canada
	Unilever Canada Inc.	1.9	Multinational (Global)	Netherlands<br>United Kingdom
	Danone Canada Inc.	1.6	Multinational (Global)	France
	George Weston Ltd.	1.6	Multinational (North America)	Canada
	Canada Bread Co.	1.6	Subsidiary (Canada)	Canada
	Kellogg Canada Inc.	1.5	Multinational (Global)	USA

Sector	Company[1]	Market share (%)[1]	Type (area served)	Location of head office
	Campbell Soup Co.	1.0	Multinational (Global)	USA
Beverage	Coca-Cola Ltd./Minute Maid Co. of Canada Inc.[2]	20.2	Multinational (Global)	USA
	PepsiCo Beverages Canada (PepsiCo Canada ULC)[3]	15.6	Multinational (Global)	USA
	Nestlé Waters (Nestlé Canada Inc.)[3]	10.8	Multinational (Global)	France
	Loblaw Companies Ltd.[3]	7.9	Retailer (Canada)	Canada
	Canada Dry Mott's Inc.	6.1	Subsidiary (Global)	Canada
	A. Lassonde Inc.	4.6	Multinational (North America)	Canada
	Sobeys Inc.[3]	2.8	Retailer (Canada)	Canada
	Sun-Rype Products Ltd.[3]	1.4	Multinational (North America)	Canada
	Ocean Spray Cranberries Inc.[3]	1.0	Multinational (Global)	USA

[1]Company names and 2018 market share percentages for Canadian packaged foods and beverages as listed in the Euromonitor International database (with sampled companies each holding ≥1% of the market share).

[2]For companies where both the brand and national brand owner (i.e., part of the same parent company) were among the top packaged food or beverage manufacturers, the higher-ranking company was listed first, followed by the affiliated company. Market shares shown reflect the combined percentages for the two companies.

[3]Company manufactures packaged foods in addition to beverages.

Supplementary Table 2. Mean (± standard deviation) Health Star Ratings of products offered by each company, presented by food category.

Food category[1]		A. Lassonde	Agropur	Campbell	Canada Bread	Canada Dry Mott's	Coca-Cola	Danone	General Mills	George Weston	Kellogg	Kraft Heinz	Loblaw	Maple Leaf	Mondelēz	Nestlé	Ocean Spray	Parmalat	PepsiCo	Saputo	Sobeys	Sun-Rype	Unilever
**Bakery products (A)**	**n[2]**	**0**	**1**	**27**	**90**	**0**	**0**	**4**	**97**	**164**	**72**	**0**	**456**	**0**	**164**	**1**	**0**	**0**	**50**	**0**	**333**	**0**	**0**
	**Mean**	**-**	**2.5**	**1.8**	**3.1**	**-**	**-**	**2.3**	**2.3**	**3.3**	**2.5**	**-**	**2.4**	**-**	**2.1**	**1.0**	**-**	**-**	**2.5**	**-**	**2.2**	**-**	**-**
	**SD[3]**	**-**	**N/A**	**0.8**	**1.3**	**-**	**-**	**0.5**	**0.9**	**0.9**	**0.9**	**-**	**1.1**	**-**	**1.1**	**N/A**	**-**	**-**	**0.8**	**-**	**1.1**	**-**	**-**
Bread (A1-A3, A24)	n	0	0	0	68	0	0	0	13	137	0	0	112	0	0	0	0	0	0	0	98	0	0
	Mean	-	-	-	3.8	-	-	-	2.1	3.6	-	-	3.5	-	-	-	-	-	-	-	3.2	-	-
	SD	-	-	-	0.5	-	-	-	0.6	0.6	-	-	0.6	-	-	-	-	-	-	-	0.9	-	-
Baked goods (A4-A10, A14, A15, A17, A22, A23)	n	0	0	13	22	0	0	0	27	11	28	0	227	0	83	0	0	0	0	0	189	0	0
	Mean	-	-	1.5	1.0	-	-	-	1.7	1.7	2.1	-	1.7	-	1.6	-	-	-	-	-	1.6	-	-
	SD	-	-	0.9	0.5	-	-	-	0.6	0.7	0.7	-	0.8	-	0.9	-	-	-	-	-	0.8	-	-
Crackers, croutons and rice cakes (A11-A13, A16, A21, A25)	n	0	1	14	0	0	0	0	7	13	10	0	89	0	73	1	0	0	21	0	26	0	0
	Mean	-	2.5	2.1	-	-	-	-	1.9	1.6	3.0	-	2.6	-	2.7	1.0	-	-	2.3	-	2.2	-	-
	SD	-	N/A	0.4	-	-	-	-	0.3	0.3	1.0	-	0.9	-	1.1	N/A	-	-	0.6	-	1.0	-	-
Grain-based, protein and energy bars (A18-A20)	n	0	0	0	0	0	0	4	50	3	34	0	28	0	8	0	0	0	29	0	20	0	0
	Mean	-	-	-	-	-	-	2.3	2.7	2.3	2.7	-	2.7	-	2.4	-	-	-	2.6	-	2.3	-	-
	SD	-	-	-	-	-	-	0.5	0.9	0.8	1.0	-	0.9	-	0.3	-	-	-	1.0	-	0.6	-	-
**Beverages (B)**	**n**	**5**	**0**	**3**	**0**	**25**	**73**	**1**	**0**	**0**	**0**	**47**	**104**	**0**	**0**	**53**	**3**	**0**	**72**	**0**	**56**	**3**	**24**
	**Mean**	**2.3**	**-**	**2.2**	**-**	**1.3**	**1.7**	**5.0**	**-**	**-**	**-**	**2.0**	**1.7**	**-**	**-**	**1.9**	**2.0**	**-**	**1.6**	**-**	**1.8**	**1.7**	**1.9**
	**SD**	**0.4**	**-**	**0.6**	**-**	**0.5**	**0.8**	**N/A**	**-**	**-**	**-**	**0.2**	**0.9**	**-**	**-**	**0.9**	**0.0**	**-**	**0.6**	**-**	**0.9**	**0.3**	**0.2**
Carbonated and non-carbonated beverages (B1)	n	5	0	3	0	25	73	1	0	0	0	43	92	0	-	31	3	0	72	0	54	3	8
	Mean	2.3	-	2.2	-	1.3	1.7	5.0	-	-	-	2.0	1.7	-	-	2.3	2.0	-	1.6	-	1.8	1.7	1.8
	SD	0.4	-	0.6	-	0.5	0.8	N/A	-	-	-	0.2	0.8	-	-	1.0	0.0	-	0.6	-	0.9	0.3	0.3
Coffee, tea and hot chocolate (B3-B5)	n	0	0	0	0	0	0	0	0	0	0	4	12	0	0	22	0	0	0	0	2	0	16
	Mean	-	-	-	-	-	-	-	-	-	-	1.9	1.6	-	-	1.5	-	-	-	-	1.0	-	2.0
	SD	-	-	-	-	-	-	-	-	-	-	0.3	0.6	-	-	0.7	-	-	-	-	0.0	-	0.0
**Cereals and other grain products (C)**	**n**	**0**	**0**	**0**	**0**	**0**	**0**	**0**	**33**	**4**	**45**	**4**	**230**	**0**	**1**	**0**	**0**	**0**	**46**	**0**	**78**	**0**	**13**
	**Mean**	**-**	**-**	**-**	**-**	**-**	**-**	**-**	**2.8**	**2.3**	**3.6**	**2.4**	**4.0**	**-**	**4.5**	**-**	**-**	**-**	**3.4**	**-**	**3.8**	**-**	**2.0**
	**SD**	**-**	**-**	**-**	**-**	**-**	**-**	**-**	**0.8**	**0.9**	**1.0**	**0.5**	**0.7**	**-**	**N/A**	**-**	**-**	**-**	**1.2**	**-**	**0.7**	**-**	**0.2**
Breakfast cereals, hot and ready-to-eat (C1-C4)	n	0	0	0	0	0	0	0	32	3	45	0	51	0	0	0	0	0	43	0	22	0	0
	Mean	-	-	-	-	-	-	-	2.9	2.5	3.6	-	3.9	-	-	-	-	-	3.5	-	3.5	-	-
	SD	-	-	-	-	-	-	-	0.8	0.9	1.0	-	0.9	-	-	-	-	-	1.1	-	0.9	-	-
Grains (e.g., rice, barley), including flavoured (C7)	n	0	0	0	0	0	0	0	1	0	0	0	59	0	0	0	0	0	2	0	12	0	12
	Mean	-	-	-	-	-	-	-	2.0	-	-	-	4.0	-	-	-	-	-	1.8	-	3.7	-	2.0
	SD	-	-	-	-	-	-	-	N/A	-	-	-	0.5	-	-	-	-	-	0.4	-	0.5	-	0.2
Pastas, including filled pastas, without sauce (C8)	n	0	0	0	0	0	0	0	0	0	0	0	106	0	0	0	0	0	0	0	37	0	0
	Mean	-	-	-	-	-	-	-	-	-	-	-	4.1	-	-	-	-	-	-	-	4.0	-	-
	SD	-	-	-	-	-	-	-	-	-	-	-	0.7	-	-	-	-	-	-	-	0.4	-	-
Other grain products (C5, C6, C10, C11)	n	0	0	0	0	0	0	0	0	1	0	4	14	0	1	0	0	0	1	0	7	0	1
	Mean	-	-	-	-	-	-	-	-	1.5	-	2.4	4.3	-	4.5	-	-	-	5.0	-	3.6	-	2.5
	SD	-	-	-	-	-	-	-	-	N/A	-	0.5	0.6	-	N/A	-	-	-	N/A	-	1.0	-	N/A
**Dairy products and substitutes (D)**	**n**	**0**	**119**	**6**	**0**	**0**	**0**	**110**	**110**	**0**	**3**	**113**	**226**	**0**	**0**	**19**	**0**	**107**	**0**	**89**	**87**	**0**	**0**
	**Mean**	**-**	**3.7**	**4.3**	**-**	**-**	**-**	**3.6**	**3.6**	**-**	**4.5**	**2.3**	**3.1**	**-**	**-**	**1.8**	**-**	**3.2**	**-**	**3.2**	**2.8**	**-**	**-**
	**SD**	**-**	**1.2**	**0.3**	**-**	**-**	**-**	**1.0**	**1.3**	**-**	**0.0**	**1.5**	**1.6**	**-**	**-**	**1.6**	**-**	**1.3**	**-**	**1.6**	**1.5**	**-**	**-**
Cheese (D1-D5)	n	0	43	0	0	0	0	0	6	0	0	105	152	0	0	0	0	53	0	44	73	0	0
	Mean	-	3.3	-	-	-	-	-	3.1	-	-	2.3	2.8	-	-	-	-	2.9	-	3.1	2.9	-	-
	SD	-	1.3	-	-	-	-	-	2.2	-	-	1.5	1.5	-	-	-	-	1.4	-	1.5	1.4	-	-
Cream and cream substitutes (D6-D8, D14)	n	0	4	0	0	0	0	6	3	0	0	5	13	0	0	11	0	3	0	13	7	0	0
	Mean	-	0.5	-	-	-	-	1.3	1.3	-	-	1.3	1.2	-	-	0.5	-	2.2	-	1.0	1.2	-	-
	SD	-	0.0	-	-	-	-	0.7	1.4	-	-	1.3	1.2	-	-	0.0	-	1.8	-	1.3	0.8	-	-
Milk and milk alternatives (D10, D11)	n	0	22	4	0	0	0	20	2	0	0	0	15	0	0	5	0	0	0	26	5	0	0
	Mean	-	4.5	4.3	-	-	-	4.1	4.0	-	-	-	3.9	-	-	3.8	-	-	-	4.4	3.2	-	-
	SD	-	0.5	0.3	-	-	-	0.6	0.0	-	-	-	1.6	-	-	0.4	-	-	-	0.3	1.8	-	-
Yogurt, yogurt drinks and shakes (D12, D13, D15)	n	0	50	2	0	0	0	84	99	0	3	3	46	0	0	3	0	51	0	6	2	0	0
	Mean	-	3.9	4.3	-	-	-	3.7	3.7	-	4.5	4.0	4.5	-	-	3.2	-	3.5	-	3.3	2.8	-	-
	SD	-	0.8	0.4	-	-	-	0.9	1.2	-	0.0	0.0	0.9	-	-	0.8	-	1.1	-	1.4	0.4	-	-
**Desserts (E)**	**n**	**0**	**0**	**0**	**0**	**0**	**0**	**17**	**1**	**0**	**0**	**41**	**102**	**0**	**0**	**115**	**0**	**0**	**0**	**0**	**66**	**0**	**62**
	**Mean**	**-**	**-**	**-**	**-**	**-**	**-**	**2.9**	**2.0**	**-**	**-**	**3.2**	**2.8**	**-**	**-**	**2.6**	**-**	**-**	**-**	**-**	**2.9**	**-**	**2.4**
	**SD**	**-**	**-**	**-**	**-**	**-**	**-**	**0.7**	**N/A**	**-**	**-**	**0.4**	**0.5**	**-**	**-**	**0.6**	**-**	**-**	**-**	**-**	**0.4**	**-**	**0.6**

Food category[1]		A. Lassonde	Agropur	Campbell	Canada Bread	Canada Dry Mott's	Coca-Cola	Danone	General Mills	George Weston	Kellogg	Kraft Heinz	Loblaw	Maple Leaf	Mondelēz	Nestlé	Ocean Spray	Parmalat	PepsiCo	Saputo	Sobeys	Sun-Rype	Unilever
**Dessert toppings and fillings (F)**	**n**	**0**	**0**	**0**	**0**	**0**	**0**	**0**	**5**	**0**	**0**	**1**	**14**	**0**	**0**	**0**	**0**	**0**	**0**	**0**	**6**	**0**	**0**
	**Mean**	**-**	**-**	**-**	**-**	**-**	**-**	**-**	**0.6**	**-**	**-**	**1.5**	**2.2**	**-**	**-**	**-**	**-**	**-**	**-**	**-**	**2.1**	**-**	**-**
	**SD**	**-**	**-**	**-**	**-**	**-**	**-**	**-**	**0.2**	**-**	**-**	**N/A**	**0.8**	**-**	**-**	**-**	**-**	**-**	**-**	**-**	**1.0**	**-**	**-**
**Eggs and egg substitutes (G)**	**n**	**0**	**0**	**0**	**0**	**0**	**0**	**0**	**0**	**0**	**0**	**0**	**13**	**0**	**0**	**0**	**0**	**0**	**0**	**0**	**3**	**0**	**0**
	**Mean**	**-**	**-**	**-**	**-**	**-**	**-**	**-**	**-**	**-**	**-**	**-**	**3.9**	**-**	**-**	**-**	**-**	**-**	**-**	**-**	**4.0**	**-**	**-**
	**SD**	**-**	**-**	**-**	**-**	**-**	**-**	**-**	**-**	**-**	**-**	**-**	**0.2**	**-**	**-**	**-**	**-**	**-**	**-**	**-**	**0.0**	**-**	**-**
**Fats and oils (H)**	**n**	**0**	**3**	**7**	**1**	**0**	**0**	**0**	**0**	**0**	**0**	**85**	**130**	**0**	**0**	**0**	**0**	**13**	**0**	**2**	**69**	**0**	**21**
	**Mean**	**-**	**0.7**	**5.0**	**1.0**	**-**	**-**	**-**	**-**	**-**	**-**	**4.4**	**3.8**	**-**	**-**	**-**	**-**	**1.3**	**-**	**0.8**	**3.6**	**-**	**3.8**
	**SD**	**-**	**0.3**	**0.0**	**N/A**	**-**	**-**	**-**	**-**	**-**	**-**	**0.5**	**1.2**	**-**	**-**	**-**	**-**	**1.2**	**-**	**0.4**	**1.4**	**-**	**0.7**
Butter, margarine, shortening, lard, etc. (H1)	n	0	3	0	1	0	0	0	0	0	0	0	18	0	0	0	0	13	0	2	13	0	10
	Mean	-	0.7	-	1.0	-	-	-	-	-	-	-	1.8	-	-	-	-	1.3	-	0.8	1.1	-	3.6
	SD	-	0.3	-	N/A	-	-	-	-	-	-	-	1.3	-	-	-	-	1.2	-	0.4	0.7	-	0.8
Mayonnaise and salad dressings (H4, H5)	n	0	0	7	0	0	0	0	0	0	0	85	64	0	0	0	0	0	0	0	44	0	9
	Mean	-	-	5.0	-	-	-	-	-	-	-	4.4	4.3	-	-	-	-	-	-	-	4.2	-	3.9
	SD	-	-	0.0	-	-	-	-	-	-	-	0.5	0.5	-	-	-	-	-	-	-	0.7	-	0.7
Oils (H2, H6)	n	0	0	0	0	0	0	0	0	0	0	0	48	0	0	0	0	0	0	0	12	0	2
	Mean	-	-	-	-	-	-	-	-	-	-	-	4.0	-	-	-	-	-	-	-	4.1	-	4.0
	SD	-	-	-	-	-	-	-	-	-	-	-	0.8	-	-	-	-	-	-	-	0.9	-	0.0
**Marine and Fresh Water Animals (I)**	**n**	**0**	**0**	**0**	**0**	**0**	**0**	**0**	**0**	**0**	**0**	**0**	**87**	**0**	**0**	**0**	**0**	**0**	**0**	**0**	**48**	**0**	**0**
	**Mean**	**-**	**-**	**-**	**-**	**-**	**-**	**-**	**-**	**-**	**-**	**-**	**3.8**	**-**	**-**	**-**	**-**	**-**	**-**	**-**	**3.9**	**-**	**-**
	**SD**	**-**	**-**	**-**	**-**	**-**	**-**	**-**	**-**	**-**	**-**	**-**	**0.6**	**-**	**-**	**-**	**-**	**-**	**-**	**-**	**0.6**	**-**	**-**
**Fruit and fruit juices (J)**	**n**	**71**	**0**	**11**	**0**	**25**	**65**	**0**	**0**	**0**	**0**	**9**	**201**	**0**	**0**	**0**	**32**	**0**	**24**	**0**	**126**	**18**	**0**
	**Mean**	**2.3**	**-**	**2.2**	**-**	**3.3**	**2.6**	**-**	**-**	**-**	**-**	**1.4**	**3.3**	**-**	**-**	**-**	**3.0**	**-**	**3.5**	**-**	**3.0**	**2.4**	**-**
	**SD**	**1.0**	**-**	**0.5**	**-**	**0.9**	**1.5**	**-**	**-**	**-**	**-**	**0.5**	**1.3**	**-**	**-**	**-**	**1.2**	**-**	**1.7**	**-**	**1.3**	**1.0**	**-**
Fruit, frozen or canned, coated or uncoated (J1-J5)	n	0	0	0	0	0	0	0	0	0	0	0	54	0	0	0	0	0	0	0	42	0	0
	Mean	-	-	-	-	-	-	-	-	-	-	-	4.3	-	-	-	-	-	-	-	4.3	-	-
	SD	-	-	-	-	-	-	-	-	-	-	-	0.4	-	-	-	-	-	-	-	0.5	-	-
Fruit juices, nectars and fruit drinks (J11)	n	71	0	11	0	8	65	0	0	0	0	9	102	0	0	0	27	0	24	0	56	18	0
	Mean	2.3	-	2.2	-	2.4	2.6	-	-	-	-	1.4	2.8	-	-	-	3.0	-	3.5	-	2.0	2.4	-
	SD	1.0	-	0.5	-	1.1	1.5	-	-	-	-	0.5	1.4	-	-	-	1.3	-	1.7	-	1.1	1.0	-
Other fruit products, e.g., apple sauce, dried fruit (J6-J10, J12)	n	0	0	0	0	17	0	0	0	0	0	0	45	0	0	0	5	0	0	0	28	0	0
	Mean	-	-	-	-	3.8	-	-	-	-	-	-	3.3	-	-	-	2.9	-	-	-	3.2	-	-
	SD	-	-	-	-	0.4	-	-	-	-	-	-	0.9	-	-	-	0.9	-	-	-	0.9	-	-
**Legumes (K)**	**n**	**0**	**0**	**0**	**0**	**0**	**0**	**0**	**0**	**0**	**0**	**0**	**59**	**2**	**0**	**0**	**0**	**0**	**0**	**0**	**18**	**0**	**0**
	**Mean**	**-**	**-**	**-**	**-**	**-**	**-**	**-**	**-**	**-**	**-**	**-**	**4.9**	**4.5**	**-**	**-**	**-**	**-**	**-**	**-**	**4.8**	**-**	**-**
	**SD**	**-**	**-**	**-**	**-**	**-**	**-**	**-**	**-**	**-**	**-**	**-**	**0.2**	**0.7**	**-**	**-**	**-**	**-**	**-**	**-**	**0.3**	**-**	**-**
**Meat, poultry, their products and substitutes (L)**	**n**	**0**	**0**	**0**	**0**	**0**	**0**	**0**	**0**	**0**	**0**	**0**	**253**	**126**	**0**	**0**	**0**	**0**	**0**	**0**	**105**	**0**	**0**
	**Mean**	**-**	**-**	**-**	**-**	**-**	**-**	**-**	**-**	**-**	**-**	**-**	**2.9**	**2.3**	**-**	**-**	**-**	**-**	**-**	**-**	**2.7**	**-**	**-**
	**SD**	**-**	**-**	**-**	**-**	**-**	**-**	**-**	**-**	**-**	**-**	**-**	**1.2**	**1.1**	**-**	**-**	**-**	**-**	**-**	**-**	**1.1**	**-**	**-**
**Miscellaneous category (M)**	n	0	0	0	0	2	0	0	44	1	1	11	81	1	7	5	0	0	17	0	44	0	1
	Mean	-	-	-	-	0.5	-	-	1.4	2.0	2.5	1.3	2.2	0.5	2.9	0.7	-	-	2.6	-	1.9	-	0.5
	SD	-	-	-	-	0.0	-	-	0.9	N/A	N/A	1.0	1.2	N/A	0.7	0.3	-	-	0.6	-	1.2	-	N/A
Baking/cooking ingredients (M1, M3, M5, M8, M11, M12)	n	0	0	0	0	0	0	0	30	1	1	11	60	1	7	0	0	0	17	0	34	0	0
	Mean	-	-	-	-	-	-	-	1.9	2.0	2.5	1.3	2.1	0.5	2.9	-	-	-	2.6	-	2.1	-	-
	SD	-	-	-	-	-	-	-	0.7	N/A	N/A	1.0	1.1	N/A	0.7	-	-	-	0.6	-	1.1	-	-
Seasoning salts and mixes (M9, M10)	n	0	0	0	0	2	0	0	14	0	0	0	19	0	0	5	0	0	0	0	10	0	1
	Mean	-	-	-	-	0.5	-	-	0.5	-	-	-	2.4	-	-	0.7	-	-	-	-	1.4	-	0.5
	SD	-	-	-	-	0.0	-	-	0.1	-	-	-	1.6	-	-	0.3	-	-	-	-	1.4	-	N/A
**Combination dishes (N)**	**n**	**0**	**0**	**6**	**2**	**0**	**0**	**0**	**45**	**0**	**0**	**47**	**243**	**20**	**0**	**84**	**0**	**0**	**0**	**0**	**150**	**0**	**22**
	**Mean**	**-**	**-**	**4.1**	**2.0**	**-**	**-**	**-**	**3.1**	**-**	**-**	**3.5**	**3.4**	**2.2**	**-**	**3.3**	**-**	**-**	**-**	**-**	**3.3**	**-**	**3.5**
	**SD**	**-**	**-**	**0.5**	**0.0**	**-**	**-**	**-**	**0.8**	**-**	**-**	**0.5**	**0.6**	**0.8**	**-**	**0.5**	**-**	**-**	**-**	**-**	**0.6**	**-**	**0.1**
**Nuts and seeds (O)**	**n**	**0**	**0**	**0**	**0**	**0**	**0**	**0**	**0**	**0**	**0**	**11**	**44**	**0**	**0**	**0**	**0**	**0**	**0**	**0**	**28**	**0**	**0**
	**Mean**	**-**	**-**	**-**	**-**	**-**	**-**	**-**	**-**	**-**	**-**	**3.1**	**4.5**	**-**	**-**	**-**	**-**	**-**	**-**	**-**	**4.4**	**-**	**-**
	**SD**	**-**	**-**	**-**	**-**	**-**	**-**	**-**	**-**	**-**	**-**	**1.2**	**0.8**	**-**	**-**	**-**	**-**	**-**	**-**	**-**	**0.9**	**-**	**-**
Nuts, seeds and flours (O1, O4)	n	0	0	0	0	0	0	0	0	0	0	0	32	0	0	0	0	0	0	0	20	0	0
	Mean	-	-	-	-	-	-	-	-	-	-	-	4.8	-	-	-	-	-	-	-	4.7	-	-
	SD	-	-	-	-	-	-	-	-	-	-	-	0.3	-	-	-	-	-	-	-	0.4	-	-
Nut butters and pastes (O2, O3)	n	0	0	0	0	0	0	0	0	0	0	11.0	12.0	0	0	0	0	0	0	0	8.0	0	0
	Mean	-	-	-	-	-	-	-	-	-	-	3.1	4.0	-	-	-	-	-	-	-	3.9	-	-

Food category[1]		A. Lassonde	Agropur	Campbell	Canada Bread	Canada Dry Mott's	Coca-Cola	Danone	General Mills	George Weston	Kellogg	Kraft Heinz	Loblaw	Maple Leaf	Mondelēz	Nestlé	Ocean Spray	Parmalat	PepsiCo	Saputo	Sobeys	Sun-Rype	Unilever
	SD	-	-	-	-	-	-	-	-	-	-	1.2	1.3	-	-	-	-	-	-	-	1.6	-	-
**Potatoes, sweet potatoes and yams (P)**	**n**	**0**	**0**	**0**	**0**	**0**	**0**	**0**	**7**	**0**	**0**	**0**	**23**	**0**	**0**	**0**	**0**	**0**	**0**	**0**	**23**	**0**	**0**
	**Mean**	-	-	-	-	-	-	-	**3.5**	-	-	-	**4.0**	-	-	-	-	-	-	-	**4.1**	-	-
	**SD**	-	-	-	-	-	-	-	**0.0**	-	-	-	**0.5**	-	-	-	-	-	-	-	**0.7**	-	-
**Salads (Q)**	**n**	**0**	**0**	**0**	**0**	**0**	**0**	**0**	**0**	**0**	**0**	**0**	**32**	**0**	**0**	**0**	**0**	**0**	**0**	**0**	**29**	**0**	**0**
	**Mean**	-	-	-	-	-	-	-	-	-	-	-	**3.7**	-	-	-	-	-	-	-	**4.1**	-	-
	**SD**	-	-	-	-	-	-	-	-	-	-	-	**0.5**	-	-	-	-	-	-	-	**0.3**	-	-
**Sauces, dips, gravies and condiments (R)**	**n**	**0**	**0**	**21**	**0**	**0**	**0**	**0**	**7**	**0**	**0**	**80**	**258**	**1**	**0**	**3**	**2**	**0**	**19**	**4**	**83**	**0**	**19**
	**Mean**	-	-	**3.3**	-	-	-	-	**3.3**	-	-	**2.5**	**2.8**	**1.0**	-	**1.5**	**2.5**	-	**3.4**	**1.5**	**2.7**	-	**2.6**
	**SD**	-	-	**0.6**	-	-	-	-	**0.4**	-	-	**0.9**	**0.9**	**N/A**	-	**1.3**	**0.0**	-	**0.7**	**0.0**	**0.8**	-	**0.8**
**Snacks (S)**	**n**	**0**	**0**	**0**	**3**	**0**	**0**	**0**	**7**	**0**	**17**	**0**	**145**	**8**	**6**	**1**	**1**	**0**	**109**	**0**	**79**	**0**	**0**
	**Mean**	-	-	-	**1.5**	-	-	-	**2.0**	-	**1.8**	-	**2.8**	**1.1**	**1.9**	**2.5**	**3.0**	-	**2.5**	-	**2.9**	-	-
	**SD**	-	-	-	**0.0**	-	-	-	**1.3**	-	**0.4**	-	**1.1**	**0.6**	**0.2**	**N/A**	**N/A**	-	**0.7**	-	**1.0**	-	-
**Soups (T)**	**n**	**0**	**0**	**135**	**0**	**0**	**0**	**0**	**0**	**0**	**0**	**0**	**66**	**0**	**0**	**0**	**0**	**0**	**0**	**0**	**41**	**0**	**51**
	**Mean**	-	-	**3.4**	-	-	-	-	-	-	-	-	**3.3**	-	-	-	-	-	-	-	**3.3**	-	**3.2**
	**SD**	-	-	**0.2**	-	-	-	-	-	-	-	-	**0.3**	-	-	-	-	-	-	-	**0.2**	-	**0.3**
**Sugars and sweets (U)**	**n**	**0**	**0**	**0**	**0**	**4**	**0**	**0**	**17**	**0**	**0**	**8**	**132**	**0**	**49**	**32**	**0**	**0**	**3**	**0**	**55**	**15**	**0**
	**Mean**	-	-	-	-	**1.5**	-	-	**1.9**	-	-	**1.1**	**1.5**	-	**0.8**	**0.7**	-	-	**2.0**	-	**1.5**	**2.5**	-
	**SD**	-	-	-	-	**0.7**	-	-	**0.5**	-	-	**0.6**	**0.7**	-	**0.4**	**0.3**	-	-	**0.5**	-	**0.6**	**0.0**	-
Confectionary (U1, U3, U4, U10, U11)	n	0	0	0	0	0	0	0	17	0	0	6	56	0	49	28	0	0	0	0	30	15	0
	Mean	-	-	-	-	-	-	-	1.9	-	-	1.2	1.2	-	0.8	0.6	-	-	-	-	1.4	2.5	-
	SD	-	-	-	-	-	-	-	0.5	-	-	0.8	0.7	-	0.4	0.2	-	-	-	-	0.5	0.0	-
Sugars and syrups (U8, U9, U12, U14, U15)	n	0	0	0	0	4	0	0	0	0	0	2	76	0	0	4	0	0	3	0	25	0	0
	Mean	-	-	-	-	1.5	-	-	-	-	-	1.0	1.7	-	-	1.3	-	-	2.0	-	1.6	-	-
	SD	-	-	-	-	0.7	-	-	-	-	-	0.0	0.7	-	-	0.3	-	-	0.5	-	0.7	-	-
**Vegetables (V)**	**n**	**1**	**0**	**6**	**0**	**8**	**0**	**0**	**9**	**0**	**0**	**3**	**200**	**0**	**0**	**0**	**0**	**0**	**0**	**0**	**106**	**0**	**4**
	**Mean**	**2.5**	-	**3.0**	-	**1.5**	-	-	**4.4**	-	-	**3.8**	**4.0**	-	-	-	-	-	-	-	**3.9**	-	**2.9**
	**SD**	**N/A**	-	**1.4**	-	**0.5**	-	-	**0.3**	-	-	**1.3**	**0.9**	-	-	-	-	-	-	-	**1.1**	-	**0.5**
Vegetables without sauce (V1, V3-V5, V8)	n	0	0	1	0	0	0	0	7	0	0	0	145	0	0	0	0	0	0	0	77	0	0
	Mean	-	-	4.5	-	-	-	-	4.4	-	-	-	4.3	-	-	-	-	-	-	-	4.3	-	-
	SD	-	-	N/A	-	-	-	-	0.4	-	-	-	0.8	-	-	-	-	-	-	-	0.8	-	-
Vegetables with sauce, pastes (V2, V9-V12)	n	0	0	0	0	0	0	0	2	0	0	2	45	0	0	0	0	0	0	0	21	0	4
	Mean	-	-	-	-	-	-	-	4.5	-	-	3.3	3.4	-	-	-	-	-	-	-	3.2	-	2.9
	SD	-	-	-	-	-	-	-	0.0	-	-	1.1	0.8	-	-	-	-	-	-	-	0.7	-	0.5
Vegetable juice and vegetable drink (V7)	n	1	0	5	0	8	0	0	0	0	0	1	10	0	0	0	0	0	0	0	8	0	0
	Mean	2.5	-	2.7	-	1.5	-	-	-	-	-	5.0	2.6	-	-	-	-	-	-	-	2.4	-	-
	SD	N/A	-	1.4	-	0.5	-	-	-	-	-	N/A	1.3	-	-	-	-	-	-	-	1.7	-	-

[1]Food categories are based on those defined in Health Canada's Table of Reference Amounts for Foods (TRA), listed in brackets. Values at the TRA major food category level are shown in bold. TRA minor food categories that were combined are indicated in brackets. If none of the sampled products fell into that category, it was omitted from the table.

[2]The number of products offered by the company in that food category (that were included in the FLIP 2017 database). Dashes indicate that the company did not offer products within that food category.

[3]SD: standard deviation

Supplementary Table 3. Median (± interquartile range) amounts of calories, sodium, saturated fat and total sugars per 100 g (or mL) and per reference amount in products offered by each company, presented by food category.

Food category[1]			A. Lassonde	Agropur	Campbell	Canada Bread	Canada Dry Mott's	Coca-Cola	Danone	General Mills	George Weston	Kellogg	Kraft Heinz	Loblaw	Maple Leaf Foods	Mondelez	Nestlé	Ocean Spray	Parmalat	PepsiCo	Saputo	Sobeys	Sun-Rype	Unilever
**Bakery products (A)**		**n[2]**	**0**	**1**	**27**	**90**	**0**	**0**	**4**	**97**	**164**	**72**	**0**	**456**	**0**	**164**	**1**	**0**	**0**	**50**	**0**	**333**	**0**	**0**
	**Calories (kcal)**	**Med[3] per 100 g/mL**	-	409.1	450.0	266.7	-	-	476.2	416.7	280.0	400.0	-	400.0	-	457.3	500.0	-	-	428.6	-	360.0	-	-
		**IQR[4] per 100 g/mL**	-	0.0	50.0	117.6	-	-	22.6	111.0	46.0	73.6	-	157.3	-	71.4	0.0	-	-	50.0	-	139.7	-	-
		**Med per RA[5]**	-	122.7	135.0	190.1	-	-	204.6	161.8	183.3	140.5	-	154.5	-	135.0	35.0	-	-	127.7	-	198.0	-	-
		**IQR per RA**	-	0.0	15.0	72.6	-	-	25.8	48.8	60.0	72.9	-	94.6	-	53.1	0.0	-	-	101.1	-	139.0	-	-
	**Sodium (mg)**	**Med per 100 g/mL**	-	409.1	500.0	375.0	-	-	232.1	333.3	455.8	410.0	-	338.3	-	362.7	642.9	-	-	328.6	-	352.1	-	-
		**IQR per 100 g/mL**	-	0.0	542.3	77.3	-	-	53.6	204.5	191.8	248.0	-	260.0	-	295.1	0.0	-	-	416.7	-	242.1	-	-
		**Med per RA**	-	122.7	150.0	277.3	-	-	104.5	128.0	292.8	126.0	-	148.5	-	100.9	45.0	-	-	111.7	-	233.1	-	-
		**IQR per RA**	-	0.0	162.7	68.0	-	-	20.4	103.2	115.3	134.2	-	176.3	-	54.3	0.0	-	-	51.2	-	201.4	-	-
	**Saturated fat (g)**	**Med per 100 g/mL**	-	4.5	2.5	0.8	-	-	4.9	5.2	0.9	3.0	-	3.4	-	3.3	8.9	-	-	2.5	-	4.3	-	-
		**IQR per 100 g/mL**	-	0.0	7.4	3.0	-	-	3.2	3.3	1.0	2.8	-	6.5	-	5.5	0.0	-	-	2.8	-	7.5	-	-
		**Med per RA**	-	1.4	0.8	0.6	-	-	2.2	2.3	0.6	1.6	-	1.5	-	1.0	0.6	-	-	0.9	-	2.3	-	-
		**IQR per RA**	-	0.0	2.2	1.9	-	-	1.5	1.3	0.5	1.6	-	3.1	-	1.8	0.0	-	-	1.4	-	5.2	-	-
	**Total sugars (g)**	**Med per 100 g/mL**	-	4.5	19.2	3.6	-	-	19.0	22.5	3.2	23.4	-	16.2	-	20.0	0.0	-	-	19.2	-	20.0	-	-
		**IQR per 100 g/mL**	-	0.0	32.9	3.9	-	-	2.5	17.9	4.3	21.7	-	24.3	-	29.5	0.0	-	-	21.4	-	25.5	-	-
		**Med per RA**	-	1.4	5.8	2.3	-	-	8.1	9.2	2.2	8.6	-	6.6	-	6.1	0.0	-	-	5.8	-	9.5	-	-
		**IQR per RA**	-	0.0	9.9	2.6	-	-	1.8	6.8	2.9	7.0	-	11.3	-	9.4	0.0	-	-	9.3	-	18.4	-	-
Bread (A1-A3, A24)		n	0	0	0	68	0	0	0	13	137	0	0	112	0	0	0	0	0	0	0	98	0	0
	Calories (kcal)	Med per 100 g/mL	-	-	-	262.0	-	-	-	294.1	270.8	-	-	267.9	-	-	-	-	-	-	-	281.6	-	-
		IQR per 100 g/mL	-	-	-	28.7	-	-	-	76.9	39.0	-	-	35.7	-	-	-	-	-	-	-	74.2	-	-
		Med per RA	-	-	-	182.7	-	-	-	161.8	183.3	-	-	187.5	-	-	-	-	-	-	-	190.4	-	-
		IQR per RA	-	-	-	46.8	-	-	-	42.3	48.2	-	-	56.8	-	-	-	-	-	-	-	62.8	-	-
	Sodium (mg)	Med per 100 g/mL	-	-	-	392.0	-	-	-	724.1	457.1	-	-	450.8	-	-	-	-	-	-	-	512.9	-	-
		IQR per 100 g/mL	-	-	-	116.5	-	-	-	119.0	161.7	-	-	198.8	-	-	-	-	-	-	-	252.1	-	-
		Med per RA	-	-	-	284.8	-	-	-	398.3	300.0	-	-	301.7	-	-	-	-	-	-	-	330.8	-	-
		IQR per RA	-	-	-	58.0	-	-	-	65.5	131.9	-	-	168.0	-	-	-	-	-	-	-	141.5	-	-
	Saturated fat (g)	Med per 100 g/mL	-	-	-	0.7	-	-	-	4.0	0.8	-	-	0.8	-	-	-	-	-	-	-	0.7	-	-
		IQR per 100 g/mL	-	-	-	0.5	-	-	-	1.2	0.8	-	-	0.9	-	-	-	-	-	-	-	1.2	-	-
		Med per RA	-	-	-	0.5	-	-	-	2.2	0.6	-	-	0.5	-	-	-	-	-	-	-	0.5	-	-
		IQR per RA	-	-	-	0.4	-	-	-	0.7	0.5	-	-	0.5	-	-	-	-	-	-	-	0.9	-	-
	Total sugars (g)	Med per 100 g/mL	-	-	-	3.2	-	-	-	5.9	3.0	-	-	3.7	-	-	-	-	-	-	-	3.5	-	-
		IQR per 100 g/mL	-	-	-	1.7	-	-	-	6.6	2.7	-	-	2.9	-	-	-	-	-	-	-	3.2	-	-
		Med per RA	-	-	-	2.2	-	-	-	3.2	2.0	-	-	2.5	-	-	-	-	-	-	-	2.0	-	-
		IQR per RA	-	-	-	1.4	-	-	-	3.6	2.0	-	-	2.3	-	-	-	-	-	-	-	1.7	-	-
Baked goods (A4-A10, A14, A15, A17, A22, A23)		n	0	0	13	22	0	0	0	27	11	28	0	227	0	83	0	0	0	0	0	189	0	0
	Calories (kcal)	Med per 100 g/mL	-	-	500.0	438.9	-	-	-	400.0	392.9	353.9	-	413.0	-	478.3	-	-	-	-	-	376.5	-	-
		IQR per 100 g/mL	-	-	90.0	56.4	-	-	-	88.7	64.3	142.2	-	105.4	-	55.9	-	-	-	-	-	124.4	-	-
		Med per RA	-	-	150.0	318.3	-	-	-	144.0	215.2	192.9	-	175.6	-	144.8	-	-	-	-	-	250.7	-	-
		IQR per RA	-	-	27.0	108.3	-	-	-	56.7	129.0	95.3	-	168.2	-	14.8	-	-	-	-	-	198.9	-	-
	Sodium (mg)	Med per 100 g/mL	-	-	307.7	343.6	-	-	-	333.3	295.8	500.0	-	263.2	-	293.1	-	-	-	-	-	294.7	-	-
		IQR per 100 g/mL	-	-	154.5	82.7	-	-	-	175.6	157.8	110.3	-	138.3	-	83.4	-	-	-	-	-	142.1	-	-
		Med per RA	-	-	92.3	243.0	-	-	-	128.0	177.9	298.3	-	117.3	-	90.0	-	-	-	-	-	217.3	-	-
		IQR per RA	-	-	46.4	179.1	-	-	-	90.0	152.7	264.4	-	164.8	-	26.6	-	-	-	-	-	182.2	-	-
	Saturated fat (g)	Med per 100 g/mL	-	-	11.5	11.0	-	-	-	5.8	6.3	3.1	-	6.7	-	6.5	-	-	-	-	-	7.1	-	-
		IQR per 100 g/mL	-	-	10.4	7.1	-	-	-	2.9	4.0	1.9	-	8.1	-	5.5	-	-	-	-	-	6.0	-	-
		Med per RA	-	-	3.5	5.0	-	-	-	2.4	4.1	1.6	-	3.5	-	1.9	-	-	-	-	-	5.0	-	-
		IQR per RA	-	-	3.1	4.6	-	-	-	0.9	3.9	1.3	-	3.4	-	1.6	-	-	-	-	-	5.3	-	-
	Total sugars (g)	Med per 100 g/mL	-	-	34.5	44.6	-	-	-	22.2	28.3	21.3	-	26.4	-	33.3	-	-	-	-	-	26.4	-	-
		IQR per 100 g/mL	-	-	11.5	35.3	-	-	-	17.1	14.6	18.9	-	11.3	-	14.3	-	-	-	-	-	14.5	-	-
		Med per RA	-	-	10.3	35.7	-	-	-	11.1	12.5	8.0	-	12.0	-	10.4	-	-	-	-	-	18.4	-	-
		IQR per RA	-	-	3.5	32.2	-	-	-	2.5	8.1	5.3	-	14.9	-	4.6	-	-	-	-	-	17.9	-	-
Crackers, croutons and rice cakes (A11-A13, A16, A21, A25)		n	0	1	14	0	0	0	0	7	13	10	0	89	0	73	1	0	0	21	0	26	0	0
	Calories (kcal)	Med per 100 g/mL	-	409.1	450.0	-	-	-	-	483.9	400.0	400.0	-	450.0	-	450.0	500.0	-	-	450.0	-	428.6	-	-
		IQR per 100 g/mL	-	0.0	0.0	-	-	-	-	20.5	50.0	0.0	-	76.9	-	55.1	0.0	-	-	90.9	-	44.6	-	-
		Med per RA	-	122.7	135.0	-	-	-	-	145.2	120.0	120.0	-	90.0	-	90.0	35.0	-	-	67.5	-	72.1	-	-
		IQR per RA	-	0.0	0.0	-	-	-	-	6.2	15.0	23.9	-	20.0	-	34.3	0.0	-	-	38.6	-	32.4	-	-
	Sodium (mg)	Med per 100 g/mL	-	409.1	850.0	-	-	-	-	483.9	1050.0	750.0	-	600.0	-	605.3	642.9	-	-	909.1	-	786.4	-	-
		IQR per 100 g/mL	-	0.0	100.0	-	-	-	-	209.9	500.0	150.0	-	235.7	-	250.0	0.0	-	-	450.0	-	548.2	-	-
		Med per RA	-	122.7	255.0	-	-	-	-	145.2	315.0	225.0	-	120.0	-	125.0	45.0	-	-	142.5	-	105.4	-	-
		IQR per RA	-	0.0	30.0	-	-	-	-	63.0	75.0	91.8	-	53.9	-	100.0	0.0	-	-	35.0	-	112.1	-	-
	Saturated fat (g)	Med per 100 g/mL	-	4.5	2.5	-	-	-	-	9.3	2.5	1.8	-	2.0	-	1.5	8.9	-	-	1.5	-	1.5	-	-
		IQR per 100 g/mL	-	0.0	1.2	-	-	-	-	6.3	0.5	1.4	-	2.1	-	3.7	0.0	-	-	0.0	-	2.4	-	-
		Med per RA	-	1.4	0.8	-	-	-	-	2.8	0.6	0.5	-	0.4	-	0.3	0.6	-	-	0.3	-	0.3	-	-
		IQR per RA	-	0.0	0.4	-	-	-	-	1.9	0.3	0.2	-	0.4	-	0.7	0.0	-	-	0.1	-	0.5	-	-
	Total sugars (g)	Med per 100 g/mL	-	4.5	0.0	-	-	-	-	3.2	0.0	7.0	-	4.8	-	5.0	0.0	-	-	5.0	-	2.0	-	-
		IQR per 100 g/mL	-	0.0	5.0	-	-	-	-	3.3	20.0	5.0	-	6.0	-	9.8	0.0	-	-	0.0	-	7.1	-	-
		Med per RA	-	1.4	0.0	-	-	-	-	1.0	0.0	1.7	-	0.9	-	1.0	0.0	-	-	1.0	-	0.5	-	-

Food category[1]			A. Lassonde	Agropur	Campbell	Canada Bread	Canada Dry Mott's	Coca-Cola	Danone	General Mills	George Weston	Kellogg	Kraft Heinz	Loblaw	Maple Leaf Foods	Mondelez	Nestlé	Ocean Spray	Parmalat	PepsiCo	Saputo	Sobeys	Sun-Rype	Unilever
		IQR per RA	-	0.0	1.5	-	-	-	-	1.0	6.0	1.3	-	1.1	-	2.0	0.0	-	-	0.6	-	1.1	-	-
Grain-based, protein and energy bars (A18-A20)		n	0	0	0	0	0	0	4	50	3	34	0	28	0	8	0	0	0	29	0	20	0	0
	Calories (kcal)	Med per 100 g/mL	-	-	-	-	-	-	476.2	426.4	425.0	404.5	-	423.1	-	434.9	-	-	-	423.1	-	441.2	-	-
		IQR per 100 g/mL	-	-	-	-	-	-	22.6	68.2	50.0	56.3	-	79.8	-	72.0	-	-	-	44.0	-	61.8	-	-
		Med per RA	-	-	-	-	-	-	204.6	169.7	127.5	140.5	-	140.5	-	140.0	-	-	-	160.0	-	161.9	-	-
		IQR per RA	-	-	-	-	-	-	25.8	46.1	15.0	51.5	-	55.3	-	38.4	-	-	-	31.4	-	58.8	-	-
	Sodium (mg)	Med per 100 g/mL	-	-	-	-	-	-	232.1	291.0	87.5	253.6	-	207.1	-	321.4	-	-	-	250.0	-	153.8	-	-
		IQR per 100 g/mL	-	-	-	-	-	-	53.6	210.7	12.5	171.8	-	108.3	-	154.1	-	-	-	69.2	-	149.1	-	-
		Med per RA	-	-	-	-	-	-	104.5	107.7	26.3	91.9	-	78.0	-	113.1	-	-	-	93.3	-	55.5	-	-
		IQR per RA	-	-	-	-	-	-	20.4	85.9	3.8	60.4	-	51.1	-	72.1	-	-	-	33.5	-	38.3	-	-
	Saturated fat (g)	Med per 100 g/mL	-	-	-	-	-	-	4.9	5.7	2.5	4.4	-	5.1	-	3.5	-	-	-	3.8	-	3.8	-	-
		IQR per 100 g/mL	-	-	-	-	-	-	3.2	3.2	6.3	5.2	-	3.4	-	3.2	-	-	-	3.4	-	3.9	-	-
		Med per RA	-	-	-	-	-	-	2.2	2.3	0.8	1.3	-	1.8	-	1.0	-	-	-	1.5	-	1.2	-	-
		IQR per RA	-	-	-	-	-	-	1.5	1.5	1.9	2.0	-	1.6	-	1.4	-	-	-	1.7	-	1.8	-	-
	Total sugars (g)	Med per 100 g/mL	-	-	-	-	-	-	19.0	27.3	32.5	31.0	-	27.0	-	25.7	-	-	-	25.7	-	29.9	-	-
		IQR per 100 g/mL	-	-	-	-	-	-	2.5	14.5	5.0	12.6	-	9.5	-	5.2	-	-	-	12.2	-	9.1	-	-
		Med per RA	-	-	-	-	-	-	8.1	10.3	9.8	9.9	-	10.3	-	8.0	-	-	-	10.3	-	11.5	-	-
		IQR per RA	-	-	-	-	-	-	1.8	7.3	1.5	5.1	-	4.7	-	2.4	-	-	-	5.2	-	6.6	-	-
**Beverages (B)**		**n**	5	0	3	0	25	73	1	0	0	0	47	104	0	0	53	3	0	72	0	56	3	24
	**Calories (kcal)**	**Med per 100 g/mL**	14.1	-	21.1	-	36.6	21.1	0.0	-	-	-	0.0	25.9	-	-	25.3	2.1	-	23.9	-	4.0	28.0	0.0
		**IQR per 100 g/mL**	5.9	-	23.5	-	44.4	33.8	0.0	-	-	-	2.0	40.0	-	-	42.4	0.0	-	33.8	-	45.0	12.0	18.1
		**Med per RA**	52.8	-	79.1	-	137.3	79.2	0.0	-	-	-	0.0	72.3	-	-	73.8	7.9	-	89.8	-	15.0	105.0	0.0
		**IQR per RA**	22.2	-	87.9	-	166.5	126.8	0.0	-	-	-	7.5	150.0	-	-	142.9	0.0	-	126.9	-	165.0	45.0	67.8
	**Sodium (mg)**	**Med per 100 g/mL**	0.0	-	8.4	-	15.5	11.3	18.0	-	-	-	4.0	5.6	-	-	3.3	15.9	-	22.0	-	6.0	4.0	0.0
		**IQR per 100 g/mL**	2.0	-	16.8	-	7.0	19.8	0.0	-	-	-	11.5	16.1	-	-	23.2	0.0	-	33.8	-	8.0	3.0	0.0
		**Med per RA**	0.0	-	31.6	-	58.1	42.3	67.5	-	-	-	15.0	21.1	-	-	12.5	59.5	-	82.6	-	22.5	15.0	0.0
		**IQR per RA**	7.5	-	62.9	-	26.4	74.1	0.0	-	-	-	43.0	62.2	-	-	58.0	0.0	-	126.7	-	30.0	11.3	0.0
	**Saturated fat (g)**	**Med per 100 g/mL**	0.0	-	0.0	-	0.0	0.0	0.0	-	-	-	0.0	0.0	-	-	0.0	0.0	-	0.0	-	0.0	0.0	0.0
		**IQR per 100 g/mL**	0.0	-	0.3	-	0.0	0.0	0.0	-	-	-	0.0	0.0	-	-	0.8	0.0	-	0.0	-	0.0	0.0	0.0
		**Med per RA**	0.0	-	0.0	-	0.0	0.0	0.0	-	-	-	0.0	0.0	-	-	0.0	0.0	-	0.0	-	0.0	0.0	0.0
		**IQR per RA**	0.0	-	1.1	-	0.0	0.0	0.0	-	-	-	0.0	0.0	-	-	2.1	0.0	-	0.0	-	0.0	0.0	0.0
	**Total sugars (g)**	**Med per 100 g/mL**	3.1	-	4.6	-	9.6	5.4	0.0	-	-	-	0.0	4.3	-	-	4.0	0.2	-	5.8	-	0.0	6.8	0.0
		**IQR per 100 g/mL**	1.3	-	3.7	-	11.3	7.9	0.0	-	-	-	0.0	10.0	-	-	9.1	0.0	-	9.7	-	11.5	3.4	4.6
		**Med per RA**	11.6	-	17.4	-	35.9	20.3	0.0	-	-	-	0.0	10.8	-	-	10.0	0.8	-	21.7	-	0.0	25.5	0.0
		**IQR per RA**	4.9	-	13.8	-	42.3	29.6	0.0	-	-	-	0.0	37.2	-	-	27.1	0.0	-	36.3	-	40.9	12.8	17.4
Carbonated and non-carbonated beverages (B1)		n	5	0	3	0	25	73	1	0	0	0	43	92	0	0	31	3	0	72	0	54	3	8
	Calories (kcal)	Med per 100 g/mL	14.1	-	21.1	-	36.6	21.1	0.0	-	-	-	0.0	16.0	-	-	0.0	2.1	-	23.9	-	3.0	28.0	19.3
		IQR per 100 g/mL	5.9	-	23.5	-	44.4	33.8	0.0	-	-	-	2.0	40.0	-	-	25.5	0.0	-	33.8	-	44.0	12.0	4.7
		Med per RA	52.8	-	79.1	-	137.3	79.2	0.0	-	-	-	0.0	60.2	-	-	0.0	7.9	-	89.8	-	11.3	105.0	72.5
		IQR per RA	22.2	-	87.9	-	166.5	126.8	0.0	-	-	-	7.5	150.0	-	-	95.6	0.0	-	126.9	-	165.0	45.0	17.5
	Sodium (mg)	Med per 100 g/mL	0.0	-	8.4	-	15.5	11.3	18.0	-	-	-	2.0	5.6	-	-	0.0	15.9	-	22.0	-	6.0	4.0	0.0
		IQR per 100 g/mL	2.0	-	16.8	-	7.0	19.8	0.0	-	-	-	8.3	10.0	-	-	2.7	0.0	-	33.8	-	7.4	3.0	2.4
		Med per RA	0.0	-	31.6	-	58.1	42.3	67.5	-	-	-	7.5	21.1	-	-	0.0	59.5	-	82.6	-	22.5	15.0	0.0
		IQR per RA	7.5	-	62.9	-	26.4	74.1	0.0	-	-	-	31.3	37.4	-	-	10.0	0.0	-	126.7	-	27.7	11.3	9.1
	Saturated fat (g)	Med per 100 g/mL	0.0	-	0.0	-	0.0	0.0	0.0	-	-	-	0.0	0.0	-	-	0.0	0.0	-	0.0	-	0.0	0.0	0.0
		IQR per 100 g/mL	0.0	-	0.3	-	0.0	0.0	0.0	-	-	-	0.0	0.0	-	-	0.0	0.0	-	0.0	-	0.0	0.0	0.0
		Med per RA	0.0	-	0.0	-	0.0	0.0	0.0	-	-	-	0.0	0.0	-	-	0.0	0.0	-	0.0	-	0.0	0.0	0.0
		IQR per RA	0.0	-	1.1	-	0.0	0.0	0.0	-	-	-	0.0	0.0	-	-	0.0	0.0	-	0.0	-	0.0	0.0	0.0
	Total sugars (g)	Med per 100 g/mL	3.1	-	4.6	-	9.6	5.4	0.0	-	-	-	0.0	2.3	-	-	0.0	0.2	-	5.8	-	0.0	6.8	4.8
		IQR per 100 g/mL	1.3	-	3.7	-	11.3	7.9	0.0	-	-	-	0.0	10.0	-	-	6.8	0.0	-	9.7	-	11.1	3.4	1.4
		Med per RA	11.6	-	17.4	-	35.9	20.3	0.0	-	-	-	0.0	8.7	-	-	0.0	0.8	-	21.7	-	0.0	25.5	18.1
		IQR per RA	4.9	-	13.8	-	42.3	29.6	0.0	-	-	-	0.0	37.5	-	-	25.6	0.0	-	36.3	-	41.6	12.8	5.2
Coffee, tea and hot chocolate (B3-B5)		n	0	0	0	0	0	0	0	0	0	0	4	12	0	0	22	0	0	0	0	2	0	16
	Calories (kcal)	Med per 100 g/mL	-	-	-	-	-	-	-	-	-	-	29.1	30.8	-	-	36.0	-	-	-	-	65.7	-	0.0
		IQR per 100 g/mL	-	-	-	-	-	-	-	-	-	-	27.6	45.3	-	-	35.2	-	-	-	-	2.9	-	0.0
		Med per RA	-	-	-	-	-	-	-	-	-	-	72.7	77.1	-	-	90.0	-	-	-	-	164.3	-	0.0
		IQR per RA	-	-	-	-	-	-	-	-	-	-	69.0	113.2	-	-	88.1	-	-	-	-	7.1	-	0.0
	Sodium (mg)	Med per 100 g/mL	-	-	-	-	-	-	-	-	-	-	41.2	81.5	-	-	32.4	-	-	-	-	72.9	-	0.0
		IQR per 100 g/mL	-	-	-	-	-	-	-	-	-	-	18.7	20.3	-	-	37.6	-	-	-	-	12.9	-	0.0
		Med per RA	-	-	-	-	-	-	-	-	-	-	102.9	203.7	-	-	81.0	-	-	-	-	182.1	-	0.0
		IQR per RA	-	-	-	-	-	-	-	-	-	-	46.8	50.7	-	-	93.9	-	-	-	-	32.1	-	0.0
	Saturated fat (g)	Med per 100 g/mL	-	-	-	-	-	-	-	-	-	-	0.8	0.7	-	-	0.8	-	-	-	-	0.9	-	0.0
		IQR per 100 g/mL	-	-	-	-	-	-	-	-	-	-	0.1	0.6	-	-	0.2	-	-	-	-	0.0	-	0.0
		Med per RA	-	-	-	-	-	-	-	-	-	-	2.1	1.7	-	-	2.1	-	-	-	-	2.1	-	0.0
		IQR per RA	-	-	-	-	-	-	-	-	-	-	0.2	1.4	-	-	0.4	-	-	-	-	0.0	-	0.0
	Total sugars (g)	Med per 100 g/mL	-	-	-	-	-	-	-	-	-	-	3.4	4.4	-	-	5.4	-	-	-	-	11.7	-	0.0
		IQR per 100 g/mL	-	-	-	-	-	-	-	-	-	-	4.6	7.5	-	-	6.4	-	-	-	-	0.3	-	0.0
		Med per RA	-	-	-	-	-	-	-	-	-	-	8.6	11.0	-	-	13.5	-	-	-	-	29.3	-	0.0
		IQR per RA	-	-	-	-	-	-	-	-	-	-	11.4	18.8	-	-	16.0	-	-	-	-	0.7	-	0.0

Food category[1]			A. Lassonde	Agropur	Campbell	Canada Bread	Canada Dry Mott's	Coca-Cola	Danone	General Mills	George Weston	Kellogg	Kraft Heinz	Loblaw	Maple Leaf Foods	Mondelez	Nestlé	Ocean Spray	Parmalat	PepsiCo	Saputo	Sobeys	Sun-Rype	Unilever
**Cereals and other grain products (C)**		**n**	**0**	**0**	**0**	**0**	**0**	**0**	**0**	**33**	**4**	**45**	**4**	**230**	**0**	**1**	**0**	**0**	**0**	**46**	**0**	**78**	**0**	**13**
	**Calories (kcal)**	**Med per 100 g/mL**	-	-	-	-	-	-	-	**384.6**	**436.4**	**375.0**	**366.7**	**355.6**	-	**366.7**	-	-	-	**373.5**	-	**365.3**	-	**372.1**
		**IQR per 100 g/mL**	-	-	-	-	-	-	-	**26.9**	**53.3**	**38.4**	**20.6**	**25.0**	-	**0.0**	-	-	-	**35.6**	-	**20.9**	-	**9.1**
		**Med per RA**	-	-	-	-	-	-	-	**125.8**	**260.0**	**193.5**	**366.7**	**235.4**	-	**110.0**	-	-	-	**148.3**	-	**270.0**	-	**167.4**
		**IQR per RA**	-	-	-	-	-	-	-	**96.5**	**54.2**	**100.1**	**92.1**	**146.0**	-	**0.0**	-	-	-	**15.0**	-	**158.9**	-	**4.1**
	**Sodium (mg)**	**Med per 100 g/mL**	-	-	-	-	-	-	-	**533.3**	**109.1**	**275.9**	**1116.7**	**11.8**	-	**33.3**	-	-	-	**394.7**	-	**17.4**	-	**1027.8**
		**IQR per 100 g/mL**	-	-	-	-	-	-	-	**255.3**	**397.7**	**281.6**	**725.0**	**249.2**	-	**0.0**	-	-	-	**430.7**	-	**216.4**	-	**181.3**
		**Med per RA**	-	-	-	-	-	-	-	**174.2**	**60.0**	**134.8**	**1116.7**	**9.0**	-	**10.0**	-	-	-	**159.7**	-	**20.0**	-	**462.5**
		**IQR per RA**	-	-	-	-	-	-	-	**45.5**	**398.8**	**79.5**	**725.0**	**138.8**	-	**0.0**	-	-	-	**158.1**	-	**107.0**	-	**81.6**
	**Saturated fat (g)**	**Med per 100 g/mL**	-	-	-	-	-	-	-	**0.8**	**2.7**	**0.6**	**0.0**	**0.4**	-	**0.0**	-	-	-	**1.0**	-	**0.5**	-	**0.0**
		**IQR per 100 g/mL**	-	-	-	-	-	-	-	**0.6**	**4.7**	**1.0**	**0.2**	**0.7**	-	**0.0**	-	-	-	**0.5**	-	**1.2**	-	**1.2**
		**Med per RA**	-	-	-	-	-	-	-	**0.4**	**1.5**	**0.3**	**0.0**	**0.3**	-	**0.0**	-	-	-	**0.4**	-	**0.4**	-	**0.0**
		**IQR per RA**	-	-	-	-	-	-	-	**0.3**	**2.4**	**0.5**	**0.2**	**0.5**	-	**0.0**	-	-	-	**0.2**	-	**0.9**	-	**0.5**
	**Total sugars (g)**	**Med per 100 g/mL**	-	-	-	-	-	-	-	**27.9**	**20.9**	**20.0**	**6.7**	**2.4**	-	**0.0**	-	-	-	**20.9**	-	**2.6**	-	**3.0**
		**IQR per 100 g/mL**	-	-	-	-	-	-	-	**8.6**	**10.2**	**7.8**	**1.7**	**3.3**	-	**0.0**	-	-	-	**21.1**	-	**2.7**	-	**2.4**
		**Med per RA**	-	-	-	-	-	-	-	**10.0**	**11.5**	**10.0**	**6.7**	**2.0**	-	**0.0**	-	-	-	**8.4**	-	**2.0**	-	**1.4**
		**IQR per RA**	-	-	-	-	-	-	-	**3.6**	**4.8**	**2.5**	**1.7**	**2.3**	-	**0.0**	-	-	-	**9.3**	-	**4.1**	-	**1.1**
Breakfast cereals, hot and ready-to-eat (C1-C4)		n	0	0	0	0	0	0	0	32	3	45	0	51	0	0	0	0	0	43	0	22	0	0
	Calories (kcal)	Med per 100 g/mL	-	-	-	-	-	-	-	386.8	454.5	375.0	-	377.8	-	-	-	-	-	375.0	-	393.9	-	-
		IQR per 100 g/mL	-	-	-	-	-	-	-	29.2	36.4	38.4	-	32.5	-	-	-	-	-	33.3	-	31.2	-	-
		Med per RA	-	-	-	-	-	-	-	125.8	250.0	193.5	-	199.1	-	-	-	-	-	148.8	-	157.6	-	-
		IQR per RA	-	-	-	-	-	-	-	96.8	20.0	100.1	-	70.0	-	-	-	-	-	15.6	-	36.5	-	-
	Sodium (mg)	Med per 100 g/mL	-	-	-	-	-	-	-	516.7	100.0	275.9	-	200.0	-	-	-	-	-	394.7	-	240.0	-	-
		IQR per 100 g/mL	-	-	-	-	-	-	-	240.0	27.3	281.6	-	324.2	-	-	-	-	-	401.5	-	351.8	-	-
		Med per RA	-	-	-	-	-	-	-	172.8	55.0	134.8	-	105.0	-	-	-	-	-	159.4	-	82.7	-	-
		IQR per RA	-	-	-	-	-	-	-	47.4	15.0	79.5	-	142.5	-	-	-	-	-	152.7	-	130.4	-	-
	Saturated fat (g)	Med per 100 g/mL	-	-	-	-	-	-	-	0.8	2.7	0.6	-	1.0	-	-	-	-	-	1.0	-	0.8	-	-
		IQR per 100 g/mL	-	-	-	-	-	-	-	0.4	8.6	1.0	-	0.8	-	-	-	-	-	0.5	-	1.3	-	-
		Med per RA	-	-	-	-	-	-	-	0.4	1.5	0.3	-	0.4	-	-	-	-	-	0.4	-	0.4	-	-
		IQR per RA	-	-	-	-	-	-	-	0.3	4.8	0.5	-	0.3	-	-	-	-	-	0.2	-	0.5	-	-
	Total sugars (g)	Med per 100 g/mL	-	-	-	-	-	-	-	28.0	23.6	20.0	-	18.2	-	-	-	-	-	21.1	-	21.8	-	-
		IQR per 100 g/mL	-	-	-	-	-	-	-	8.6	6.4	7.8	-	18.5	-	-	-	-	-	13.2	-	19.7	-	-
		Med per RA	-	-	-	-	-	-	-	10.2	13.0	10.0	-	9.0	-	-	-	-	-	8.4	-	9.9	-	-
		IQR per RA	-	-	-	-	-	-	-	3.3	3.5	2.5	-	8.2	-	-	-	-	-	6.8	-	10.1	-	-
Grains (e.g., rice, barley), including flavoured (C7)		n	0	0	0	0	0	0	0	1	0	0	0	59	0	0	0	0	0	2	0	12	0	12
	Calories (kcal)	Med per 100 g/mL	-	-	-	-	-	-	-	351.9	-	-	-	355.6	-	-	-	-	-	335.7	-	359.6	-	372.1
		IQR per 100 g/mL	-	-	-	-	-	-	-	0.0	-	-	-	22.2	-	-	-	-	-	7.1	-	22.2	-	7.9
		Med per RA	-	-	-	-	-	-	-	158.3	-	-	-	160.0	-	-	-	-	-	151.1	-	161.8	-	167.4
		IQR per RA	-	-	-	-	-	-	-	0.0	-	-	-	10.0	-	-	-	-	-	3.2	-	10.0	-	3.5
	Sodium (mg)	Med per 100 g/mL	-	-	-	-	-	-	-	870.4	-	-	-	0.0	-	-	-	-	-	1650.0	-	0.0	-	1037.1
		IQR per 100 g/mL	-	-	-	-	-	-	-	0.0	-	-	-	22.2	-	-	-	-	-	121.4	-	11.4	-	130.9
		Med per RA	-	-	-	-	-	-	-	391.7	-	-	-	0.0	-	-	-	-	-	742.5	-	0.0	-	466.7
		IQR per RA	-	-	-	-	-	-	-	0.0	-	-	-	10.5	-	-	-	-	-	54.6	-	5.1	-	58.9
	Saturated fat (g)	Med per 100 g/mL	-	-	-	-	-	-	-	0.4	-	-	-	0.2	-	-	-	-	-	0.1	-	0.0	-	0.4
		IQR per 100 g/mL	-	-	-	-	-	-	-	0.0	-	-	-	0.7	-	-	-	-	-	0.1	-	0.7	-	1.2
		Med per RA	-	-	-	-	-	-	-	0.2	-	-	-	0.2	-	-	-	-	-	0.0	-	0.0	-	0.2
		IQR per RA	-	-	-	-	-	-	-	0.0	-	-	-	0.3	-	-	-	-	-	0.0	-	0.3	-	0.5
	Total sugars (g)	Med per 100 g/mL	-	-	-	-	-	-	-	3.7	-	-	-	0.0	-	-	-	-	-	4.3	-	0.0	-	3.0
		IQR per 100 g/mL	-	-	-	-	-	-	-	0.0	-	-	-	0.0	-	-	-	-	-	1.4	-	0.0	-	2.3
		Med per RA	-	-	-	-	-	-	-	1.7	-	-	-	0.0	-	-	-	-	-	1.9	-	0.0	-	1.3
		IQR per RA	-	-	-	-	-	-	-	0.0	-	-	-	0.0	-	-	-	-	-	0.6	-	0.0	-	1.0
Pastas, including filled pastas, without sauce (C8)		n	0	0	0	0	0	0	0	0	0	0	0	106	0	0	0	0	0	0	0	37	0	0
	Calories (kcal)	Med per 100 g/mL	-	-	-	-	-	-	-	-	-	-	-	352.9	-	-	-	-	-	-	-	364.7	-	-
		IQR per 100 g/mL	-	-	-	-	-	-	-	-	-	-	-	23.5	-	-	-	-	-	-	-	69.1	-	-
		Med per RA	-	-	-	-	-	-	-	-	-	-	-	306.0	-	-	-	-	-	-	-	310.0	-	-
		IQR per RA	-	-	-	-	-	-	-	-	-	-	-	10.0	-	-	-	-	-	-	-	288.3	-	-
	Sodium (mg)	Med per 100 g/mL	-	-	-	-	-	-	-	-	-	-	-	11.8	-	-	-	-	-	-	-	5.9	-	-
		IQR per 100 g/mL	-	-	-	-	-	-	-	-	-	-	-	193.6	-	-	-	-	-	-	-	23.5	-	-
		Med per RA	-	-	-	-	-	-	-	-	-	-	-	10.0	-	-	-	-	-	-	-	5.0	-	-
		IQR per RA	-	-	-	-	-	-	-	-	-	-	-	222.8	-	-	-	-	-	-	-	37.4	-	-
	Saturated fat (g)	Med per 100 g/mL	-	-	-	-	-	-	-	-	-	-	-	0.4	-	-	-	-	-	-	-	0.5	-	-
		IQR per 100 g/mL	-	-	-	-	-	-	-	-	-	-	-	0.5	-	-	-	-	-	-	-	0.9	-	-
		Med per RA	-	-	-	-	-	-	-	-	-	-	-	0.4	-	-	-	-	-	-	-	0.4	-	-
		IQR per RA	-	-	-	-	-	-	-	-	-	-	-	0.4	-	-	-	-	-	-	-	0.8	-	-
	Total sugars (g)	Med per 100 g/mL	-	-	-	-	-	-	-	-	-	-	-	2.4	-	-	-	-	-	-	-	2.4	-	-
		IQR per 100 g/mL	-	-	-	-	-	-	-	-	-	-	-	1.3	-	-	-	-	-	-	-	1.8	-	-
		Med per RA	-	-	-	-	-	-	-	-	-	-	-	2.0	-	-	-	-	-	-	-	2.0	-	-
		IQR per RA	-	-	-	-	-	-	-	-	-	-	-	0.9	-	-	-	-	-	-	-	1.1	-	-
Other grain		n	0	0	0	0	0	0	0	0	1	0	4	14	0	1	0	0	0	1	0	7	0	1

Food category[1]			A. Lassonde	Agropur	Campbell	Canada Bread	Canada Dry Mott's	Coca-Cola	Danone	General Mills	George Weston	Kellogg	Kraft Heinz	Loblaw	Maple Leaf Foods	Mondelez	Nestlé	Ocean Spray	Parmalat	PepsiCo	Saputo	Sobeys	Sun-Rype	Unilever
products (C5, C6, C10, C11)	Calories (kcal)	Med per 100 g/mL	-	-	-	-	-	-	-	-	386.7	-	366.7	341.7	-	366.7	-	-	-	333.3	-	366.7	-	285.7
		IQR per 100 g/mL	-	-	-	-	-	-	-	-	0.0	-	20.6	33.3	-	0.0	-	-	-	0.0	-	0.0	-	0.0
		Med per RA	-	-	-	-	-	-	-	-	386.7	-	366.7	100.0	-	110.0	-	-	-	50.0	-	110.0	-	28.6
		IQR per RA	-	-	-	-	-	-	-	-	0.0	-	92.1	7.5	-	0.0	-	-	-	0.0	-	128.3	-	0.0
	Sodium (mg)	Med per 100 g/mL	-	-	-	-	-	-	-	-	1600.0	-	1116.7	0.0	-	33.3	-	-	-	16.7	-	0.0	-	0.0
		IQR per 100 g/mL	-	-	-	-	-	-	-	-	0.0	-	725.0	0.0	-	0.0	-	-	-	0.0	-	741.7	-	0.0
		Med per RA	-	-	-	-	-	-	-	-	1600.0	-	1116.7	0.0	-	10.0	-	-	-	2.5	-	0.0	-	0.0
		IQR per RA	-	-	-	-	-	-	-	-	0.0	-	725.0	0.0	-	0.0	-	-	-	0.0	-	700.8	-	0.0
	Saturated fat (g)	Med per 100 g/mL	-	-	-	-	-	-	-	-	1.3	-	0.0	0.0	-	0.0	-	-	-	0.7	-	0.0	-	0.0
		IQR per 100 g/mL	-	-	-	-	-	-	-	-	0.0	-	0.2	0.8	-	0.0	-	-	-	0.0	-	0.0	-	0.0
		Med per RA	-	-	-	-	-	-	-	-	1.3	-	0.0	0.0	-	0.0	-	-	-	0.1	-	0.0	-	0.0
		IQR per RA	-	-	-	-	-	-	-	-	0.0	-	0.2	0.2	-	0.0	-	-	-	0.0	-	0.0	-	0.0
	Total sugars (g)	Med per 100 g/mL	-	-	-	-	-	-	-	-	6.7	-	6.7	0.0	-	0.0	-	-	-	3.3	-	3.3	-	28.6
		IQR per 100 g/mL	-	-	-	-	-	-	-	-	0.0	-	1.7	2.5	-	0.0	-	-	-	0.0	-	1.7	-	0.0
		Med per RA	-	-	-	-	-	-	-	-	6.7	-	6.7	0.0	-	0.0	-	-	-	0.5	-	1.0	-	2.9
		IQR per RA	-	-	-	-	-	-	-	-	0.0	-	1.7	0.4	-	0.0	-	-	-	0.0	-	2.8	-	0.0
**Dairy products and substitutes (D)**		n	0	119	6	0	0	0	110	110	0	3	113	226	0	0	19	0	107	0	89	87	0	0
	Calories (kcal)	Med per 100 g/mL	-	85.7	48.0	-	-	-	90.0	80.0	-	64.2	300.0	300.0	-	-	233.3	-	157.9	-	233.3	333.3	-	-
		IQR per 100 g/mL	-	269.3	38.0	-	-	-	40.5	35.0	-	0.0	125.7	264.8	-	-	157.0	-	266.7	-	269.3	114.3	-	-
		Med per RA	-	116.7	120.0	-	-	-	159.6	140.5	-	160.5	90.0	110.0	-	-	35.0	-	120.0	-	114.3	105.0	-	-
		IQR per RA	-	40.0	95.0	-	-	-	85.0	70.0	-	0.0	45.6	40.0	-	-	127.9	-	76.0	-	70.0	40.0	-	-
	Sodium (mg)	Med per 100 g/mL	-	46.0	60.0	-	-	-	54.3	44.0	-	74.3	700.0	529.8	-	-	56.0	-	133.3	-	200.0	666.7	-	-
		IQR per 100 g/mL	-	589.3	25.5	-	-	-	27.8	12.9	-	1.7	533.3	660.3	-	-	50.0	-	637.5	-	650.0	261.7	-	-
		Med per RA	-	95.0	150.0	-	-	-	95.0	78.8	-	185.8	210.0	180.0	-	-	5.0	-	112.0	-	170.0	200.0	-	-
		IQR per RA	-	113.8	63.8	-	-	-	49.9	22.5	-	4.2	60.0	145.0	-	-	129.3	-	122.5	-	100.0	92.5	-	-
	Saturated fat (g)	Med per 100 g/mL	-	1.5	0.1	-	-	-	1.3	1.2	-	0.2	16.1	12.5	-	-	1.3	-	3.6	-	6.7	16.7	-	-
		IQR per 100 g/mL	-	15.3	0.1	-	-	-	0.7	1.5	-	0.0	9.3	18.3	-	-	0.5	-	15.5	-	15.5	8.3	-	-
		Med per RA	-	2.5	0.4	-	-	-	2.0	2.2	-	0.4	4.0	4.1	-	-	0.4	-	3.6	-	3.5	5.0	-	-
		IQR per RA	-	4.0	0.3	-	-	-	2.1	3.1	-	0.0	3.0	4.0	-	-	1.8	-	3.9	-	4.5	3.0	-	-
	Total sugars (g)	Med per 100 g/mL	-	4.4	6.8	-	-	-	10.0	10.5	-	6.4	0.0	0.0	-	-	13.6	-	3.3	-	4.8	0.0	-	-
		IQR per 100 g/mL	-	10.0	6.2	-	-	-	4.7	7.4	-	0.2	7.1	5.6	-	-	25.3	-	12.0	-	6.7	3.8	-	-
		Med per RA	-	10.0	17.0	-	-	-	17.5	19.0	-	16.0	0.0	0.0	-	-	5.0	-	3.2	-	1.0	0.0	-	-
		IQR per RA	-	17.5	15.5	-	-	-	11.0	13.9	-	0.4	2.0	6.0	-	-	15.0	-	21.0	-	12.0	1.0	-	-
Cheese (D1-D5)		n	0	43	0	0	0	0	0	6	0	0	105	152	0	0	0	0	53	0	44	73	0	0
	Calories (kcal)	Med per 100 g/mL	-	350.0	-	-	-	-	-	118.2	-	-	300.0	350.0	-	-	-	-	366.7	-	333.3	366.7	-	-
		IQR per 100 g/mL	-	105.8	-	-	-	-	-	95.5	-	-	126.2	108.2	-	-	-	-	100.0	-	94.0	100.0	-	-
		Med per RA	-	105.0	-	-	-	-	-	60.0	-	-	90.0	110.0	-	-	-	-	114.3	-	112.1	110.0	-	-
		IQR per RA	-	25.0	-	-	-	-	-	22.5	-	-	37.7	30.0	-	-	-	-	30.0	-	20.0	30.0	-	-
	Sodium (mg)	Med per 100 g/mL	-	666.7	-	-	-	-	-	263.6	-	-	700.0	666.7	-	-	-	-	700.0	-	700.0	666.7	-	-
		IQR per 100 g/mL	-	200.0	-	-	-	-	-	284.0	-	-	690.8	252.6	-	-	-	-	100.0	-	300.0	200.0	-	-
		Med per RA	-	200.0	-	-	-	-	-	117.5	-	-	210.0	210.0	-	-	-	-	210.0	-	215.0	200.0	-	-
		IQR per RA	-	70.0	-	-	-	-	-	68.8	-	-	62.5	90.5	-	-	-	-	40.0	-	62.0	60.0	-	-
	Saturated fat (g)	Med per 100 g/mL	-	16.7	-	-	-	-	-	3.4	-	-	16.1	16.7	-	-	-	-	16.7	-	16.7	16.7	-	-
		IQR per 100 g/mL	-	6.6	-	-	-	-	-	8.7	-	-	8.3	8.1	-	-	-	-	6.7	-	6.7	5.7	-	-
		Med per RA	-	5.3	-	-	-	-	-	1.6	-	-	4.8	5.5	-	-	-	-	6.0	-	6.0	5.0	-	-
		IQR per RA	-	2.3	-	-	-	-	-	3.2	-	-	2.8	2.7	-	-	-	-	1.7	-	2.1	2.5	-	-
	Total sugars (g)	Med per 100 g/mL	-	0.0	-	-	-	-	-	2.4	-	-	0.0	0.0	-	-	-	-	0.0	-	0.0	0.0	-	-
		IQR per 100 g/mL	-	0.0	-	-	-	-	-	1.4	-	-	6.7	0.0	-	-	-	-	0.0	-	0.0	0.0	-	-
		Med per RA	-	0.0	-	-	-	-	-	1.0	-	-	0.0	0.0	-	-	-	-	0.0	-	0.0	0.0	-	-
		IQR per RA	-	0.0	-	-	-	-	-	0.0	-	-	2.0	0.0	-	-	-	-	0.0	-	0.0	0.0	-	-
Cream and cream substitutes (D6-D8, D14)		n	0	4	0	0	0	0	6	3	0	0	5	13	0	0	11	0	3	0	13	7	0	0
	Calories (kcal)	Med per 100 g/mL	-	233.3	-	-	-	-	133.3	166.7	-	-	229.9	150.0	-	-	233.3	-	100.0	-	233.3	150.0	-	-
		IQR per 100 g/mL	-	208.3	-	-	-	-	50.0	125.0	-	-	205.8	100.0	-	-	266.7	-	50.0	-	33.3	138.6	-	-
		Med per RA	-	35.0	-	-	-	-	20.0	50.0	-	-	34.5	30.0	-	-	35.0	-	30.0	-	35.0	30.0	-	-
		IQR per RA	-	31.3	-	-	-	-	7.5	12.5	-	-	30.9	29.1	-	-	25.0	-	15.0	-	5.0	23.3	-	-
	Sodium (mg)	Med per 100 g/mL	-	33.3	-	-	-	-	0.0	50.0	-	-	11.2	66.7	-	-	33.3	-	133.3	-	100.0	66.7	-	-
		IQR per 100 g/mL	-	66.7	-	-	-	-	0.0	8.3	-	-	34.5	45.2	-	-	58.3	-	16.7	-	180.0	62.9	-	-
		Med per RA	-	5.0	-	-	-	-	0.0	15.0	-	-	1.7	14.3	-	-	3.3	-	40.0	-	15.0	5.0	-	-
		IQR per RA	-	10.0	-	-	-	-	0.0	5.0	-	-	5.2	17.4	-	-	2.0	-	5.0	-	27.0	15.8	-	-
	Saturated fat (g)	Med per 100 g/mL	-	13.3	-	-	-	-	3.3	8.3	-	-	6.3	8.3	-	-	1.3	-	3.3	-	6.7	6.7	-	-
		IQR per 100 g/mL	-	13.3	-	-	-	-	2.5	10.0	-	-	11.6	9.7	-	-	15.8	-	4.2	-	3.3	6.9	-	-
		Med per RA	-	2.0	-	-	-	-	0.5	2.5	-	-	0.9	1.0	-	-	0.2	-	1.0	-	1.0	1.0	-	-
		IQR per RA	-	2.0	-	-	-	-	0.4	1.3	-	-	1.7	2.1	-	-	0.1	-	1.3	-	0.5	0.9	-	-
	Total sugars (g)	Med per 100 g/mL	-	3.3	-	-	-	-	21.7	3.3	-	-	11.5	5.0	-	-	33.3	-	6.7	-	33.3	4.5	-	-
		IQR per 100 g/mL	-	6.7	-	-	-	-	17.5	1.7	-	-	18.3	6.7	-	-	0.0	-	1.7	-	26.7	6.1	-	-
		Med per RA	-	0.5	-	-	-	-	3.3	1.0	-	-	1.7	1.0	-	-	5.0	-	2.0	-	5.0	1.0	-	-
		IQR per RA	-	1.0	-	-	-	-	2.6	0.5	-	-	2.7	2.0	-	-	3.8	-	0.5	-	4.0	1.3	-	-
Milk and milk alternatives (D10,		n	0	22	4	0	0	0	20	2	0	0	0	15	0	0	5	0	0	0	26	5	0	0
	Calories	Med per 100 g/mL	-	52.0	38.0	-	-	-	24.0	52.0	-	-	-	52.0	-	-	62.5	-	-	-	54.0	100.0	-	-

Food category[1]			A. Lassonde	Agropur	Campbell	Canada Bread	Canada Dry Mott's	Coca-Cola	Danone	General Mills	George Weston	Kellogg	Kraft Heinz	Loblaw	Maple Leaf Foods	Mondelez	Nestlé	Ocean Spray	Parmalat	PepsiCo	Saputo	Sobeys	Sun-Rype	Unilever
D11)	(kcal)	IQR per 100 g/mL	-	24.0	35.0	-	-	-	15.5	4.0	-	-	-	48.0	-	-	18.0	-	-	-	20.0	57.3	-	-
		Med per RA	-	130.0	95.0	-	-	-	60.0	130.0	-	-	-	90.0	-	-	156.3	-	-	-	135.0	60.0	-	-
		IQR per RA	-	60.0	87.5	-	-	-	38.8	10.0	-	-	-	67.5	-	-	45.0	-	-	-	50.0	70.0	-	-
	Sodium (mg)	Med per 100 g/mL	-	44.0	60.0	-	-	-	50.0	40.0	-	-	-	48.0	-	-	50.0	-	-	-	50.0	98.0	-	-
		IQR per 100 g/mL	-	30.0	8.5	-	-	-	50.5	0.0	-	-	-	39.0	-	-	20.6	-	-	-	23.0	33.3	-	-
		Med per RA	-	110.0	150.0	-	-	-	125.0	100.0	-	-	-	90.0	-	-	125.0	-	-	-	125.0	15.0	-	-
		IQR per RA	-	75.0	21.3	-	-	-	126.3	0.0	-	-	-	75.0	-	-	51.5	-	-	-	57.5	100.0	-	-
	Saturated fat (g)	Med per 100 g/mL	-	0.6	0.1	-	-	-	0.2	1.6	-	-	-	0.4	-	-	0.8	-	-	-	0.6	0.6	-	-
		IQR per 100 g/mL	-	0.6	0.1	-	-	-	1.5	0.4	-	-	-	1.5	-	-	0.1	-	-	-	0.6	3.3	-	-
		Med per RA	-	1.5	0.4	-	-	-	0.5	4.0	-	-	-	1.0	-	-	2.0	-	-	-	1.5	0.5	-	-
		IQR per RA	-	1.5	0.3	-	-	-	3.7	1.0	-	-	-	0.7	-	-	0.2	-	-	-	1.5	1.0	-	-
	Total sugars (g)	Med per 100 g/mL	-	4.4	4.6	-	-	-	2.8	4.4	-	-	-	4.8	-	-	8.0	-	-	-	5.0	11.6	-	-
		IQR per 100 g/mL	-	0.7	5.4	-	-	-	3.2	0.0	-	-	-	2.9	-	-	0.4	-	-	-	5.2	6.7	-	-
		Med per RA	-	11.0	11.5	-	-	-	7.0	11.0	-	-	-	11.0	-	-	20.0	-	-	-	12.5	11.0	-	-
		IQR per RA	-	1.8	13.5	-	-	-	8.0	0.0	-	-	-	7.5	-	-	1.0	-	-	-	13.0	11.0	-	-
Yogurt, yogurt drinks and shakes (D12, D13, D15)		n	0	50	2	0	0	0	84	99	0	3	3	46	0	0	3	0	51	0	6	2	0	0
	Calories (kcal)	Med per 100 g/mL	-	74.3	65.3	-	-	-	100.0	80.0	-	64.2	73.7	79.7	-	-	84.7	-	100.0	-	85.5	166.7	-	-
		IQR per 100 g/mL	-	31.4	21.3	-	-	-	14.0	30.0	-	0.0	0.0	28.3	-	-	8.5	-	20.0	-	24.8	13.3	-	-
		Med per RA	-	130.2	163.3	-	-	-	175.0	141.0	-	160.5	184.2	140.0	-	-	211.9	-	175.0	-	213.7	291.7	-	-
		IQR per RA	-	47.9	53.3	-	-	-	17.5	43.8	-	0.0	0.0	39.9	-	-	21.2	-	35.0	-	62.0	23.3	-	-
	Sodium (mg)	Med per 100 g/mL	-	45.0	53.2	-	-	-	54.3	44.0	-	74.3	50.0	40.0	-	-	63.6	-	50.0	-	69.4	51.0	-	-
		IQR per 100 g/mL	-	5.0	31.2	-	-	-	22.1	12.9	-	1.7	2.6	9.0	-	-	2.1	-	25.5	-	27.0	5.7	-	-
		Med per RA	-	78.8	133.1	-	-	-	96.3	78.8	-	185.8	125.0	71.0	-	-	158.9	-	87.5	-	173.4	89.3	-	-
		IQR per RA	-	8.2	78.1	-	-	-	39.8	22.5	-	4.2	6.6	19.9	-	-	5.3	-	44.6	-	67.5	9.9	-	-
	Saturated fat (g)	Med per 100 g/mL	-	0.6	0.4	-	-	-	1.4	1.2	-	0.2	0.5	0.8	-	-	1.3	-	1.2	-	1.3	3.6	-	-
		IQR per 100 g/mL	-	1.4	0.3	-	-	-	0.5	1.3	-	0.0	0.0	1.2	-	-	0.2	-	1.0	-	0.9	0.4	-	-
		Med per RA	-	1.0	1.1	-	-	-	2.5	2.2	-	0.4	1.3	1.5	-	-	3.2	-	2.1	-	3.2	6.3	-	-
		IQR per RA	-	2.5	0.9	-	-	-	0.9	2.4	-	0.0	0.0	2.0	-	-	0.5	-	1.8	-	2.2	0.7	-	-
	Total sugars (g)	Med per 100 g/mL	-	10.0	9.0	-	-	-	10.8	10.9	-	6.4	11.1	8.6	-	-	13.1	-	12.7	-	12.7	14.3	-	-
		IQR per 100 g/mL	-	3.9	1.8	-	-	-	1.9	3.0	-	0.2	0.0	5.0	-	-	2.1	-	4.1	-	6.1	1.0	-	-
		Med per RA	-	17.5	22.6	-	-	-	19.0	19.3	-	16.0	27.6	15.1	-	-	32.8	-	22.2	-	31.9	25.1	-	-
		IQR per RA	-	7.0	4.6	-	-	-	4.2	5.0	-	0.4	0.0	9.3	-	-	5.3	-	7.3	-	15.3	1.8	-	-
**Desserts (E)**		**n**	**0**	**0**	**0**	**0**	**0**	**0**	**17**	**1**	**0**	**0**	**41**	**102**	**0**	**0**	**115**	**0**	**0**	**0**	**0**	**66**	**0**	**62**
	**Calories (kcal)**	**Med per 100 g/mL**	-	-	-	-	-	-	**128.0**	**228.8**	-	-	**54.8**	**126.5**	-	-	**150.0**	-	-	-	-	**104.0**	-	**178.6**
		**IQR per 100 g/mL**	-	-	-	-	-	-	**34.0**	**0.0**	-	-	**46.3**	**55.3**	-	-	**124.0**	-	-	-	-	**49.1**	-	**134.0**
		**Med per RA**	-	-	-	-	-	-	**187.5**	**297.5**	-	-	**71.2**	**180.5**	-	-	**211.5**	-	-	-	-	**165.4**	-	**206.5**
		**IQR per RA**	-	-	-	-	-	-	**82.6**	**0.0**	-	-	**60.2**	**127.2**	-	-	**113.3**	-	-	-	-	**85.2**	-	**83.9**
	**Sodium (mg)**	**Med per 100 g/mL**	-	-	-	-	-	-	**66.2**	**38.1**	-	-	**109.4**	**48.0**	-	-	**52.0**	-	-	-	-	**48.0**	-	**51.0**
		**IQR per 100 g/mL**	-	-	-	-	-	-	**51.2**	**0.0**	-	-	**141.5**	**50.3**	-	-	**29.1**	-	-	-	-	**27.7**	-	**39.5**
		**Med per RA**	-	-	-	-	-	-	**82.7**	**49.6**	-	-	**142.2**	**68.2**	-	-	**82.7**	-	-	-	-	**82.7**	-	**60.2**
		**IQR per RA**	-	-	-	-	-	-	**94.8**	**0.0**	-	-	**183.9**	**80.4**	-	-	**45.4**	-	-	-	-	**55.8**	-	**70.3**
	**Saturated fat (g)**	**Med per 100 g/mL**	-	-	-	-	-	-	**2.0**	**11.0**	-	-	**0.0**	**1.8**	-	-	**3.6**	-	-	-	-	**1.6**	-	**5.7**
		**IQR per 100 g/mL**	-	-	-	-	-	-	**3.2**	**0.0**	-	-	**1.0**	**3.8**	-	-	**6.5**	-	-	-	-	**2.8**	-	**5.9**
		**Med per RA**	-	-	-	-	-	-	**2.6**	**14.3**	-	-	**0.0**	**2.5**	-	-	**6.0**	-	-	-	-	**2.3**	-	**6.8**
		**IQR per RA**	-	-	-	-	-	-	**4.5**	**0.0**	-	-	**1.3**	**6.9**	-	-	**7.2**	-	-	-	-	**5.8**	-	**4.5**
	**Total sugars (g)**	**Med per 100 g/mL**	-	-	-	-	-	-	**14.0**	**14.4**	-	-	**13.0**	**15.0**	-	-	**16.0**	-	-	-	-	**12.8**	-	**18.4**
		**IQR per 100 g/mL**	-	-	-	-	-	-	**2.2**	**0.0**	-	-	**11.3**	**4.8**	-	-	**6.8**	-	-	-	-	**4.0**	-	**7.4**
		**Med per RA**	-	-	-	-	-	-	**19.5**	**18.7**	-	-	**16.9**	**21.1**	-	-	**22.6**	-	-	-	-	**21.1**	-	**19.6**
		**IQR per RA**	-	-	-	-	-	-	**9.4**	**0.0**	-	-	**14.6**	**7.3**	-	-	**10.0**	-	-	-	-	**6.5**	-	**11.1**
**Dessert toppings and fillings (F)**		**n**	**0**	**0**	**0**	**0**	**0**	**0**	**0**	**5**	**0**	**0**	**1**	**14**	**0**	**0**	**0**	**0**	**0**	**0**	**0**	**6**	**0**	**0**
	**Calories (kcal)**	**Med per 100 g/mL**	-	-	-	-	-	-	-	**424.2**	-	-	**346.2**	**286.7**	-	-	-	-	-	-	-	**223.3**	-	-
		**IQR per 100 g/mL**	-	-	-	-	-	-	-	**41.7**	-	-	**0.0**	**176.9**	-	-	-	-	-	-	-	**249.8**	-	-
		**Med per RA**	-	-	-	-	-	-	-	**148.5**	-	-	**103.8**	**105.2**	-	-	-	-	-	-	-	**121.7**	-	-
		**IQR per RA**	-	-	-	-	-	-	-	**14.6**	-	-	**0.0**	**37.8**	-	-	-	-	-	-	-	**37.8**	-	-
	**Sodium (mg)**	**Med per 100 g/mL**	-	-	-	-	-	-	-	**212.1**	-	-	**76.9**	**139.4**	-	-	-	-	-	-	-	**86.7**	-	-
		**IQR per 100 g/mL**	-	-	-	-	-	-	-	**41.7**	-	-	**0.0**	**211.9**	-	-	-	-	-	-	-	**136.0**	-	-
		**Med per RA**	-	-	-	-	-	-	-	**74.2**	-	-	**23.1**	**48.4**	-	-	-	-	-	-	-	**45.4**	-	-
		**IQR per RA**	-	-	-	-	-	-	-	**14.6**	-	-	**0.0**	**60.2**	-	-	-	-	-	-	-	**48.2**	-	-
	**Saturated fat (g)**	**Med per 100 g/mL**	-	-	-	-	-	-	-	**12.5**	-	-	**0.0**	**0.6**	-	-	-	-	-	-	-	**2.1**	-	-
		**IQR per 100 g/mL**	-	-	-	-	-	-	-	**4.9**	-	-	**0.0**	**3.8**	-	-	-	-	-	-	-	**4.8**	-	-
		**Med per RA**	-	-	-	-	-	-	-	**4.4**	-	-	**0.0**	**0.3**	-	-	-	-	-	-	-	**0.8**	-	-
		**IQR per RA**	-	-	-	-	-	-	-	**1.7**	-	-	**0.0**	**1.5**	-	-	-	-	-	-	-	**1.5**	-	-
	**Total sugars (g)**	**Med per 100 g/mL**	-	-	-	-	-	-	-	**57.6**	-	-	**61.5**	**41.3**	-	-	-	-	-	-	-	**32.0**	-	-
		**IQR per 100 g/mL**	-	-	-	-	-	-	-	**6.8**	-	-	**0.0**	**18.3**	-	-	-	-	-	-	-	**28.9**	-	-
		**Med per RA**	-	-	-	-	-	-	-	**20.2**	-	-	**18.5**	**15.7**	-	-	-	-	-	-	-	**19.9**	-	-
		**IQR per RA**	-	-	-	-	-	-	-	**2.4**	-	-	**0.0**	**9.7**	-	-	-	-	-	-	-	**0.9**	-	-
**Eggs and egg substitutes (G)**		**n**	**0**	**0**	**0**	**0**	**0**	**0**	**0**	**0**	**0**	**0**	**0**	**13**	**0**	**0**	**0**	**0**	**0**	**0**	**0**	**3**	**0**	**0**
	**Calories (kcal)**	**Med per 100 g/mL**	-	-	-	-	-	-	-	-	-	-	-	**132.1**	-	-	-	-	-	-	-	**132.1**	-	-
		**IQR per 100 g/mL**	-	-	-	-	-	-	-	-	-	-	-	**5.9**	-	-	-	-	-	-	-	**45.2**	-	-

Food category[1]			A. Lassonde	Agropur	Campbell	Canada Bread	Canada Dry Mott's	Coca-Cola	Danone	General Mills	George Weston	Kellogg	Kraft Heinz	Loblaw	Maple Leaf Foods	Mondelez	Nestlé	Ocean Spray	Parmalat	PepsiCo	Saputo	Sobeys	Sun-Rype	Unilever
		Med per RA	-	-	-	-	-	-	-	-	-	-	-	132.1	-	-	-	-	-	-	-	132.1	-	-
		IQR per RA	-	-	-	-	-	-	-	-	-	-	-	5.9	-	-	-	-	-	-	-	45.2	-	-
	Sodium (mg)	Med per 100 g/mL	-	-	-	-	-	-	-	-	-	-	-	129.3	-	-	-	-	-	-	-	129.3	-	-
		IQR per 100 g/mL	-	-	-	-	-	-	-	-	-	-	-	7.8	-	-	-	-	-	-	-	18.0	-	-
		Med per RA	-	-	-	-	-	-	-	-	-	-	-	129.3	-	-	-	-	-	-	-	129.3	-	-
		IQR per RA	-	-	-	-	-	-	-	-	-	-	-	7.8	-	-	-	-	-	-	-	18.0	-	-
	Saturated fat (g)	Med per 100 g/mL	-	-	-	-	-	-	-	-	-	-	-	2.8	-	-	-	-	-	-	-	2.8	-	-
		IQR per 100 g/mL	-	-	-	-	-	-	-	-	-	-	-	0.4	-	-	-	-	-	-	-	1.7	-	-
		Med per RA	-	-	-	-	-	-	-	-	-	-	-	2.8	-	-	-	-	-	-	-	2.8	-	-
		IQR per RA	-	-	-	-	-	-	-	-	-	-	-	0.4	-	-	-	-	-	-	-	1.7	-	-
	Total sugars (g)	Med per 100 g/mL	-	-	-	-	-	-	-	-	-	-	-	0.0	-	-	-	-	-	-	-	0.0	-	-
		IQR per 100 g/mL	-	-	-	-	-	-	-	-	-	-	-	0.0	-	-	-	-	-	-	-	0.0	-	-
		Med per RA	-	-	-	-	-	-	-	-	-	-	-	0.0	-	-	-	-	-	-	-	0.0	-	-
		IQR per RA	-	-	-	-	-	-	-	-	-	-	-	0.0	-	-	-	-	-	-	-	0.0	-	-
Fats and oils (H)		n	0	3	7	1	0	0	0	0	0	0	85	130	0	0	0	0	13	0	2	69	0	21
	Calories (kcal)	Med per 100 g/mL	-	700.0	166.7	900.0	-	-	-	-	-	-	300.0	666.7	-	-	-	-	700.0	-	700.0	466.7	-	666.7
		IQR per 100 g/mL	-	0.0	50.0	0.0	-	-	-	-	-	-	200.0	466.7	-	-	-	-	0.0	-	0.0	400.0	-	350.0
		Med per RA	-	70.0	50.0	90.0	-	-	-	-	-	-	80.0	80.0	-	-	-	-	70.0	-	70.0	80.0	-	70.0
		IQR per RA	-	0.0	15.0	0.0	-	-	-	-	-	-	60.0	20.0	-	-	-	-	0.0	-	0.0	30.0	-	40.0
	Sodium (mg)	Med per 100 g/mL	-	800.0	600.0	0.0	-	-	-	-	-	-	833.3	500.0	-	-	-	-	550.0	-	350.0	633.3	-	633.3
		IQR per 100 g/mL	-	400.0	100.0	0.0	-	-	-	-	-	-	266.7	700.0	-	-	-	-	600.0	-	350.0	733.3	-	100.0
		Med per RA	-	80.0	180.0	0.0	-	-	-	-	-	-	240.0	65.0	-	-	-	-	55.0	-	35.0	110.0	-	70.0
		IQR per RA	-	40.0	30.0	0.0	-	-	-	-	-	-	100.0	207.5	-	-	-	-	60.0	-	35.0	220.0	-	41.8
	Saturated fat (g)	Med per 100 g/mL	-	45.0	0.0	40.0	-	-	-	-	-	-	2.7	6.7	-	-	-	-	50.0	-	50.0	6.7	-	10.0
		IQR per 100 g/mL	-	0.0	3.3	0.0	-	-	-	-	-	-	1.3	6.7	-	-	-	-	15.0	-	0.0	11.7	-	3.3
		Med per RA	-	4.5	0.0	4.0	-	-	-	-	-	-	0.8	1.0	-	-	-	-	5.0	-	5.0	1.4	-	1.0
		IQR per RA	-	0.0	1.0	0.0	-	-	-	-	-	-	0.5	1.2	-	-	-	-	1.5	-	0.0	1.4	-	0.1
	Total sugars (g)	Med per 100 g/mL	-	0.0	6.7	0.0	-	-	-	-	-	-	6.7	0.0	-	-	-	-	0.0	-	0.0	0.0	-	0.0
		IQR per 100 g/mL	-	0.0	6.7	0.0	-	-	-	-	-	-	6.7	6.7	-	-	-	-	0.0	-	0.0	6.7	-	0.0
		Med per RA	-	0.0	2.0	0.0	-	-	-	-	-	-	2.0	0.0	-	-	-	-	0.0	-	0.0	0.0	-	0.0
		IQR per RA	-	0.0	2.0	0.0	-	-	-	-	-	-	3.0	2.0	-	-	-	-	0.0	-	0.0	2.0	-	0.0
Butter, margarine, shortening, lard, etc. (H1)		n	0	3	0	1	0	0	0	0	0	0	0	18	0	0	0	0	13	0	2	13	0	10
	Calories (kcal)	Med per 100 g/mL	-	700.0	-	900.0	-	-	-	-	-	-	-	700.0	-	-	-	-	700.0	-	700.0	700.0	-	650.0
		IQR per 100 g/mL	-	0.0	-	0.0	-	-	-	-	-	-	-	197.2	-	-	-	-	0.0	-	0.0	0.0	-	287.5
		Med per RA	-	70.0	-	90.0	-	-	-	-	-	-	-	70.0	-	-	-	-	70.0	-	70.0	70.0	-	65.0
		IQR per RA	-	0.0	-	0.0	-	-	-	-	-	-	-	19.7	-	-	-	-	0.0	-	0.0	0.0	-	28.8
	Sodium (mg)	Med per 100 g/mL	-	800.0	-	0.0	-	-	-	-	-	-	-	375.0	-	-	-	-	550.0	-	350.0	450.0	-	600.0
		IQR per 100 g/mL	-	400.0	-	0.0	-	-	-	-	-	-	-	600.0	-	-	-	-	600.0	-	350.0	500.0	-	87.5
		Med per RA	-	80.0	-	0.0	-	-	-	-	-	-	-	37.5	-	-	-	-	55.0	-	35.0	45.0	-	60.0
		IQR per RA	-	40.0	-	0.0	-	-	-	-	-	-	-	60.0	-	-	-	-	60.0	-	35.0	50.0	-	8.8
	Saturated fat (g)	Med per 100 g/mL	-	45.0	-	40.0	-	-	-	-	-	-	-	37.5	-	-	-	-	50.0	-	50.0	40.0	-	10.0
		IQR per 100 g/mL	-	0.0	-	0.0	-	-	-	-	-	-	-	38.8	-	-	-	-	15.0	-	0.0	20.0	-	0.0
		Med per RA	-	4.5	-	4.0	-	-	-	-	-	-	-	3.8	-	-	-	-	5.0	-	5.0	4.0	-	1.0
		IQR per RA	-	0.0	-	0.0	-	-	-	-	-	-	-	3.9	-	-	-	-	1.5	-	0.0	2.0	-	0.0
	Total sugars (g)	Med per 100 g/mL	-	0.0	-	0.0	-	-	-	-	-	-	-	0.0	-	-	-	-	0.0	-	0.0	0.0	-	0.0
		IQR per 100 g/mL	-	0.0	-	0.0	-	-	-	-	-	-	-	0.0	-	-	-	-	0.0	-	0.0	0.0	-	0.0
		Med per RA	-	0.0	-	0.0	-	-	-	-	-	-	-	0.0	-	-	-	-	0.0	-	0.0	0.0	-	0.0
		IQR per RA	-	0.0	-	0.0	-	-	-	-	-	-	-	0.0	-	-	-	-	0.0	-	0.0	0.0	-	0.0
Mayonnaise and salad dressings (H4, H5)		n	0	0	7	0	0	0	0	0	0	0	85	64	0	0	0	0	0	0	0	44	0	9
	Calories (kcal)	Med per 100 g/mL	-	-	166.7	-	-	-	-	-	-	-	300.0	333.3	-	-	-	-	-	-	-	397.7	-	642.9
		IQR per 100 g/mL	-	-	50.0	-	-	-	-	-	-	-	200.0	233.3	-	-	-	-	-	-	-	233.3	-	333.3
		Med per RA	-	-	50.0	-	-	-	-	-	-	-	80.0	90.0	-	-	-	-	-	-	-	90.0	-	96.4
		IQR per RA	-	-	15.0	-	-	-	-	-	-	-	60.0	40.0	-	-	-	-	-	-	-	52.0	-	50.0
	Sodium (mg)	Med per 100 g/mL	-	-	600.0	-	-	-	-	-	-	-	833.3	700.0	-	-	-	-	-	-	-	733.3	-	678.6
		IQR per 100 g/mL	-	-	100.0	-	-	-	-	-	-	-	266.7	166.7	-	-	-	-	-	-	-	215.5	-	166.7
		Med per RA	-	-	180.0	-	-	-	-	-	-	-	240.0	210.0	-	-	-	-	-	-	-	204.7	-	101.8
		IQR per RA	-	-	30.0	-	-	-	-	-	-	-	100.0	111.3	-	-	-	-	-	-	-	129.6	-	25.0
	Saturated fat (g)	Med per 100 g/mL	-	-	0.0	-	-	-	-	-	-	-	2.7	3.3	-	-	-	-	-	-	-	3.3	-	6.7
		IQR per 100 g/mL	-	-	3.3	-	-	-	-	-	-	-	1.3	4.0	-	-	-	-	-	-	-	4.3	-	3.8
		Med per RA	-	-	0.0	-	-	-	-	-	-	-	0.8	1.0	-	-	-	-	-	-	-	1.0	-	1.0
		IQR per RA	-	-	1.0	-	-	-	-	-	-	-	0.5	1.5	-	-	-	-	-	-	-	1.1	-	0.6
	Total sugars (g)	Med per 100 g/mL	-	-	6.7	-	-	-	-	-	-	-	6.7	6.7	-	-	-	-	-	-	-	6.7	-	0.0
		IQR per 100 g/mL	-	-	6.7	-	-	-	-	-	-	-	6.7	6.7	-	-	-	-	-	-	-	13.3	-	0.0
		Med per RA	-	-	2.0	-	-	-	-	-	-	-	2.0	2.0	-	-	-	-	-	-	-	2.0	-	0.0
		IQR per RA	-	-	2.0	-	-	-	-	-	-	-	3.0	1.3	-	-	-	-	-	-	-	3.1	-	0.0
Oils (H2, H6)		n	0	0	0	0	0	0	0	0	0	0	0	48	0	0	0	0	0	0	0	12	0	2
	Calories (kcal)	Med per 100 g/mL	-	-	-	-	-	-	-	-	-	-	-	800.0	-	-	-	-	-	-	-	800.0	-	800.0
		IQR per 100 g/mL	-	-	-	-	-	-	-	-	-	-	-	0.0	-	-	-	-	-	-	-	0.0	-	0.0
		Med per RA	-	-	-	-	-	-	-	-	-	-	-	80.0	-	-	-	-	-	-	-	80.0	-	80.0

Food category[1]			A. Lassonde	Agropur	Campbell	Canada Bread	Canada Dry Mott's	Coca-Cola	Danone	General Mills	George Weston	Kellogg	Kraft Heinz	Loblaw	Maple Leaf Foods	Mondelez	Nestlé	Ocean Spray	Parmalat	PepsiCo	Saputo	Sobeys	Sun-Rype	Unilever
		IQR per RA	-	-	-	-	-	-	-	-	-	-	-	0.0	-	-	-	-	-	-	-	19.0	-	0.0
	Sodium (mg)	Med per 100 g/mL	-	-	-	-	-	-	-	-	-	-	-	0.0	-	-	-	-	-	-	-	0.0	-	0.0
		IQR per 100 g/mL	-	-	-	-	-	-	-	-	-	-	-	0.0	-	-	-	-	-	-	-	0.0	-	0.0
		Med per RA	-	-	-	-	-	-	-	-	-	-	-	0.0	-	-	-	-	-	-	-	0.0	-	0.0
		IQR per RA	-	-	-	-	-	-	-	-	-	-	-	0.0	-	-	-	-	-	-	-	0.0	-	0.0
	Saturated fat (g)	Med per 100 g/mL	-	-	-	-	-	-	-	-	-	-	-	10.0	-	-	-	-	-	-	-	12.5	-	10.0
		IQR per 100 g/mL	-	-	-	-	-	-	-	-	-	-	-	10.0	-	-	-	-	-	-	-	11.3	-	0.0
		Med per RA	-	-	-	-	-	-	-	-	-	-	-	1.0	-	-	-	-	-	-	-	1.3	-	1.0
		IQR per RA	-	-	-	-	-	-	-	-	-	-	-	1.0	-	-	-	-	-	-	-	1.1	-	0.0
	Total sugars (g)	Med per 100 g/mL	-	-	-	-	-	-	-	-	-	-	-	0.0	-	-	-	-	-	-	-	0.0	-	0.0
		IQR per 100 g/mL	-	-	-	-	-	-	-	-	-	-	-	0.0	-	-	-	-	-	-	-	0.0	-	0.0
		Med per RA	-	-	-	-	-	-	-	-	-	-	-	0.0	-	-	-	-	-	-	-	0.0	-	0.0
		IQR per RA	-	-	-	-	-	-	-	-	-	-	-	0.0	-	-	-	-	-	-	-	0.0	-	0.0
**Marine and fresh water animals (I)**		**n**	**0**	**0**	**0**	**0**	**0**	**0**	**0**	**0**	**0**	**0**	**0**	**87**	**0**	**0**	**0**	**0**	**0**	**0**	**0**	**48**	**0**	**0**
	**Calories (kcal)**	**Med per 100 g/mL**	-	-	-	-	-	-	-	-	-	-	-	**105.9**	-	-	-	-	-	-	-	**108.1**	-	-
		**IQR per 100 g/mL**	-	-	-	-	-	-	-	-	-	-	-	**91.9**	-	-	-	-	-	-	-	**62.4**	-	-
		**Med per RA**	-	-	-	-	-	-	-	-	-	-	-	**102.3**	-	-	-	-	-	-	-	**100.0**	-	-
		**IQR per RA**	-	-	-	-	-	-	-	-	-	-	-	**115.3**	-	-	-	-	-	-	-	**74.0**	-	-
	**Sodium (mg)**	**Med per 100 g/mL**	-	-	-	-	-	-	-	-	-	-	-	**357.7**	-	-	-	-	-	-	-	**346.9**	-	-
		**IQR per 100 g/mL**	-	-	-	-	-	-	-	-	-	-	-	**234.3**	-	-	-	-	-	-	-	**190.5**	-	-
		**Med per RA**	-	-	-	-	-	-	-	-	-	-	-	**322.9**	-	-	-	-	-	-	-	**340.0**	-	-
		**IQR per RA**	-	-	-	-	-	-	-	-	-	-	-	**300.2**	-	-	-	-	-	-	-	**413.7**	-	-
	**Saturated fat (g)**	**Med per 100 g/mL**	-	-	-	-	-	-	-	-	-	-	-	**0.8**	-	-	-	-	-	-	-	**0.4**	-	-
		**IQR per 100 g/mL**	-	-	-	-	-	-	-	-	-	-	-	**1.6**	-	-	-	-	-	-	-	**0.8**	-	-
		**Med per RA**	-	-	-	-	-	-	-	-	-	-	-	**0.6**	-	-	-	-	-	-	-	**0.3**	-	-
		**IQR per RA**	-	-	-	-	-	-	-	-	-	-	-	**1.2**	-	-	-	-	-	-	-	**1.0**	-	-
	**Total sugars (g)**	**Med per 100 g/mL**	-	-	-	-	-	-	-	-	-	-	-	**0.0**	-	-	-	-	-	-	-	**0.0**	-	-
		**IQR per 100 g/mL**	-	-	-	-	-	-	-	-	-	-	-	**1.7**	-	-	-	-	-	-	-	**2.0**	-	-
		**Med per RA**	-	-	-	-	-	-	-	-	-	-	-	**0.0**	-	-	-	-	-	-	-	**0.0**	-	-
		**IQR per RA**	-	-	-	-	-	-	-	-	-	-	-	**1.6**	-	-	-	-	-	-	-	**2.5**	-	-
**Fruit and fruit juices (J)**		**n**	**71**	**0**	**11**	**0**	**25**	**65**	**0**	**0**	**0**	**0**	**9**	**201**	**0**	**0**	**0**	**32**	**0**	**24**	**0**	**126**	**18**	**0**
	**Calories (kcal)**	**Med per 100 g/mL**	**44.0**	-	**52.0**	-	**45.0**	**48.0**	-	-	-	-	**38.9**	**48.0**	-	-	-	**44.0**	-	**45.3**	-	**50.0**	**48.0**	-
		**IQR per 100 g/mL**	**5.0**	-	**10.0**	-	**9.6**	**3.9**	-	-	-	-	**24.9**	**20.3**	-	-	-	**37.0**	-	**12.0**	-	**26.7**	**4.0**	-
		**Med per RA**	**110.0**	-	**130.0**	-	**59.5**	**120.0**	-	-	-	-	**97.2**	**110.0**	-	-	-	**110.0**	-	**113.3**	-	**100.2**	**120.0**	-
		**IQR per RA**	**12.5**	-	**25.0**	-	**38.1**	**9.7**	-	-	-	-	**62.2**	**50.0**	-	-	-	**90.0**	-	**30.0**	-	**42.1**	**10.0**	-
	**Sodium (mg)**	**Med per 100 g/mL**	**6.0**	-	**10.0**	-	**0.0**	**5.9**	-	-	-	-	**8.3**	**4.0**	-	-	-	**8.0**	-	**0.0**	-	**5.7**	**10.0**	-
		**IQR per 100 g/mL**	**4.0**	-	**13.6**	-	**13.5**	**6.0**	-	-	-	-	**4.3**	**10.0**	-	-	-	**8.0**	-	**4.0**	-	**14.0**	**3.5**	-
		**Med per RA**	**15.0**	-	**25.0**	-	**0.0**	**14.7**	-	-	-	-	**20.8**	**6.7**	-	-	-	**20.0**	-	**0.0**	-	**9.7**	**25.0**	-
		**IQR per RA**	**10.0**	-	**33.9**	-	**20.0**	**15.0**	-	-	-	-	**10.8**	**15.0**	-	-	-	**20.0**	-	**10.0**	-	**30.0**	**8.8**	-
	**Saturated fat (g)**	**Med per 100 g/mL**	**0.0**	-	**0.0**	-	**0.0**	**0.0**	-	-	-	-	**0.0**	**0.0**	-	-	-	**0.0**	-	**0.0**	-	**0.0**	**0.0**	-
		**IQR per 100 g/mL**	**0.0**	-	**0.0**	-	**0.0**	**0.0**	-	-	-	-	**0.0**	**0.0**	-	-	-	**0.0**	-	**0.0**	-	**0.0**	**0.0**	-
		**Med per RA**	**0.0**	-	**0.0**	-	**0.0**	**0.0**	-	-	-	-	**0.0**	**0.0**	-	-	-	**0.0**	-	**0.0**	-	**0.0**	**0.0**	-
		**IQR per RA**	**0.0**	-	**0.0**	-	**0.0**	**0.0**	-	-	-	-	**0.0**	**0.0**	-	-	-	**0.0**	-	**0.0**	-	**0.0**	**0.0**	-
	**Total sugars (g)**	**Med per 100 g/mL**	**10.0**	-	**10.4**	-	**9.9**	**10.8**	-	-	-	-	**9.4**	**10.0**	-	-	-	**10.8**	-	**9.2**	-	**10.5**	**11.6**	-
		**IQR per 100 g/mL**	**1.6**	-	**2.0**	-	**1.9**	**2.0**	-	-	-	-	**6.6**	**5.2**	-	-	-	**8.9**	-	**2.1**	-	**6.5**	**2.3**	-
		**Med per RA**	**25.0**	-	**26.0**	-	**11.9**	**27.0**	-	-	-	-	**23.6**	**22.0**	-	-	-	**27.0**	-	**23.0**	-	**21.2**	**29.0**	-
		**IQR per RA**	**4.0**	-	**5.0**	-	**9.5**	**5.0**	-	-	-	-	**16.5**	**12.7**	-	-	-	**18.0**	-	**5.3**	-	**14.8**	**5.8**	-
Fruit, frozen or canned, coated or uncoated (J1-J5)		n	0	0	0	0	0	0	0	0	0	0	0	54	0	0	0	0	0	0	0	42	0	0
	Calories (kcal)	Med per 100 g/mL	-	-	-	-	-	-	-	-	-	-	-	50.0	-	-	-	-	-	-	-	55.8	-	-
		IQR per 100 g/mL	-	-	-	-	-	-	-	-	-	-	-	24.0	-	-	-	-	-	-	-	24.2	-	-
		Med per RA	-	-	-	-	-	-	-	-	-	-	-	66.8	-	-	-	-	-	-	-	74.0	-	-
		IQR per RA	-	-	-	-	-	-	-	-	-	-	-	37.5	-	-	-	-	-	-	-	48.7	-	-
	Sodium (mg)	Med per 100 g/mL	-	-	-	-	-	-	-	-	-	-	-	0.0	-	-	-	-	-	-	-	0.0	-	-
		IQR per 100 g/mL	-	-	-	-	-	-	-	-	-	-	-	4.5	-	-	-	-	-	-	-	6.5	-	-
		Med per RA	-	-	-	-	-	-	-	-	-	-	-	0.0	-	-	-	-	-	-	-	0.0	-	-
		IQR per RA	-	-	-	-	-	-	-	-	-	-	-	6.7	-	-	-	-	-	-	-	9.8	-	-
	Saturated fat (g)	Med per 100 g/mL	-	-	-	-	-	-	-	-	-	-	-	0.0	-	-	-	-	-	-	-	0.0	-	-
		IQR per 100 g/mL	-	-	-	-	-	-	-	-	-	-	-	0.0	-	-	-	-	-	-	-	0.0	-	-
		Med per RA	-	-	-	-	-	-	-	-	-	-	-	0.0	-	-	-	-	-	-	-	0.0	-	-
		IQR per RA	-	-	-	-	-	-	-	-	-	-	-	0.0	-	-	-	-	-	-	-	0.0	-	-
	Total sugars (g)	Med per 100 g/mL	-	-	-	-	-	-	-	-	-	-	-	8.6	-	-	-	-	-	-	-	8.9	-	-
		IQR per 100 g/mL	-	-	-	-	-	-	-	-	-	-	-	5.8	-	-	-	-	-	-	-	5.6	-	-
		Med per RA	-	-	-	-	-	-	-	-	-	-	-	12.0	-	-	-	-	-	-	-	13.4	-	-
		IQR per RA	-	-	-	-	-	-	-	-	-	-	-	8.6	-	-	-	-	-	-	-	12.0	-	-
Fruit juices, nectars and fruit drinks (J11)		n	71	0	11	0	8	65	0	0	0	0	9	102	0	0	0	27	0	24	0	56	18	0
	Calories (kcal)	Med per 100 g/mL	44.0	-	52.0	-	36.0	48.0	-	-	-	-	38.9	48.0	-	-	-	44.0	-	45.3	-	45.0	48.0	-
		IQR per 100 g/mL	5.0	-	10.0	-	44.0	3.9	-	-	-	-	24.9	6.0	-	-	-	32.0	-	12.0	-	10.5	4.0	-
		Med per RA	110.0	-	130.0	-	90.0	120.0	-	-	-	-	97.2	120.0	-	-	-	110.0	-	113.3	-	112.5	120.0	-
		IQR per RA	12.5	-	25.0	-	110.0	9.7	-	-	-	-	62.2	15.0	-	-	-	80.0	-	30.0	-	26.3	10.0	-

Food category[1]			A. Lassonde	Agropur	Campbell	Canada Bread	Canada Dry Mott's	Coca-Cola	Danone	General Mills	George Weston	Kellogg	Kraft Heinz	Loblaw	Maple Leaf Foods	Mondelez	Nestlé	Ocean Spray	Parmalat	PepsiCo	Saputo	Sobeys	Sun-Rype	Unilever
	Sodium (mg)	Med per 100 g/mL	6.0	-	10.0	-	30.0	5.9	-	-	-	-	8.3	4.0	-	-	-	8.0	-	0.0	-	14.0	10.0	-
		IQR per 100 g/mL	4.0	-	13.6	-	34.7	6.0	-	-	-	-	4.3	8.3	-	-	-	7.0	-	4.0	-	10.0	3.5	-
		Med per RA	15.0	-	25.0	-	75.0	14.7	-	-	-	-	20.8	10.0	-	-	-	20.0	-	0.0	-	35.0	25.0	-
		IQR per RA	10.0	-	33.9	-	86.7	15.0	-	-	-	-	10.8	20.8	-	-	-	17.5	-	10.0	-	25.0	8.8	-
	Saturated fat (g)	Med per 100 g/mL	0.0	-	0.0	-	0.0	0.0	-	-	-	-	0.0	0.0	-	-	-	0.0	-	0.0	-	0.0	0.0	-
		IQR per 100 g/mL	0.0	-	0.0	-	0.0	0.0	-	-	-	-	0.0	0.0	-	-	-	0.0	-	0.0	-	0.0	0.0	-
		Med per RA	0.0	-	0.0	-	0.0	0.0	-	-	-	-	0.0	0.0	-	-	-	0.0	-	0.0	-	0.0	0.0	-
		IQR per RA	0.0	-	0.0	-	0.0	0.0	-	-	-	-	0.0	0.0	-	-	-	0.0	-	0.0	-	0.0	0.0	-
	Total sugars (g)	Med per 100 g/mL	10.0	-	10.4	-	8.0	10.8	-	-	-	-	9.4	9.6	-	-	-	10.8	-	9.2	-	10.2	11.6	-
		IQR per 100 g/mL	1.6	-	2.0	-	7.7	2.0	-	-	-	-	6.6	2.6	-	-	-	7.6	-	2.1	-	2.8	2.3	-
		Med per RA	25.0	-	26.0	-	20.1	27.0	-	-	-	-	23.6	24.0	-	-	-	27.0	-	23.0	-	25.5	29.0	-
		IQR per RA	4.0	-	5.0	-	19.3	5.0	-	-	-	-	16.5	6.6	-	-	-	19.0	-	5.3	-	7.0	5.8	-
Other fruit products, e.g., apple sauce, dried fruit (J6-J10, J12)		n	0	0	0	0	17	0	0	0	0	0	0	45	0	0	0	5	0	0	0	28	0	0
	Calories (kcal)	Med per 100 g/mL	-	-	-	-	45.0	-	-	-	-	-	-	275.0	-	-	-	325.0	-	-	-	300.0	-	-
		IQR per 100 g/mL	-	-	-	-	9.0	-	-	-	-	-	-	275.0	-	-	-	0.0	-	-	-	245.1	-	-
		Med per RA	-	-	-	-	49.5	-	-	-	-	-	-	100.0	-	-	-	130.0	-	-	-	115.0	-	-
		IQR per RA	-	-	-	-	9.9	-	-	-	-	-	-	67.5	-	-	-	0.0	-	-	-	52.1	-	-
	Sodium (mg)	Med per 100 g/mL	-	-	-	-	0.0	-	-	-	-	-	-	0.0	-	-	-	0.0	-	-	-	0.0	-	-
		IQR per 100 g/mL	-	-	-	-	0.0	-	-	-	-	-	-	20.0	-	-	-	0.0	-	-	-	12.7	-	-
		Med per RA	-	-	-	-	0.0	-	-	-	-	-	-	0.0	-	-	-	0.0	-	-	-	0.0	-	-
		IQR per RA	-	-	-	-	0.0	-	-	-	-	-	-	10.0	-	-	-	0.0	-	-	-	9.8	-	-
	Saturated fat (g)	Med per 100 g/mL	-	-	-	-	0.0	-	-	-	-	-	-	0.0	-	-	-	0.0	-	-	-	0.0	-	-
		IQR per 100 g/mL	-	-	-	-	0.0	-	-	-	-	-	-	0.0	-	-	-	0.0	-	-	-	0.0	-	-
		Med per RA	-	-	-	-	0.0	-	-	-	-	-	-	0.0	-	-	-	0.0	-	-	-	0.0	-	-
		IQR per RA	-	-	-	-	0.0	-	-	-	-	-	-	0.0	-	-	-	0.0	-	-	-	0.0	-	-
	Total sugars (g)	Med per 100 g/mL	-	-	-	-	9.9	-	-	-	-	-	-	46.7	-	-	-	72.5	-	-	-	33.8	-	-
		IQR per 100 g/mL	-	-	-	-	0.9	-	-	-	-	-	-	51.5	-	-	-	0.0	-	-	-	49.9	-	-
		Med per RA	-	-	-	-	10.9	-	-	-	-	-	-	17.0	-	-	-	29.0	-	-	-	15.8	-	-
		IQR per RA	-	-	-	-	1.0	-	-	-	-	-	-	12.5	-	-	-	0.0	-	-	-	16.4	-	-
**Legumes (K)**		**n**	**0**	**0**	**0**	**0**	**0**	**0**	**0**	**0**	**0**	**0**	**0**	**59**	**2**	**0**	**0**	**0**	**0**	**0**	**0**	**18**	**0**	**0**
	**Calories (kcal)**	**Med per 100 g/mL**	-	-	-	-	-	-	-	-	-	-	-	**120.0**	**175.4**	-	-	-	-	-	-	**84.0**	-	-
		**IQR per 100 g/mL**	-	-	-	-	-	-	-	-	-	-	-	**250.0**	**8.8**	-	-	-	-	-	-	**46.0**	-	-
		**Med per RA**	-	-	-	-	-	-	-	-	-	-	-	**105.0**	**149.1**	-	-	-	-	-	-	**102.5**	-	-
		**IQR per RA**	-	-	-	-	-	-	-	-	-	-	-	**26.3**	**7.5**	-	-	-	-	-	-	**25.4**	-	-
	**Sodium (mg)**	**Med per 100 g/mL**	-	-	-	-	-	-	-	-	-	-	-	**8.0**	**433.6**	-	-	-	-	-	-	**74.0**	-	-
		**IQR per 100 g/mL**	-	-	-	-	-	-	-	-	-	-	-	**67.6**	**421.7**	-	-	-	-	-	-	**93.5**	-	-
		**Med per RA**	-	-	-	-	-	-	-	-	-	-	-	**3.5**	**368.5**	-	-	-	-	-	-	**92.5**	-	-
		**IQR per RA**	-	-	-	-	-	-	-	-	-	-	-	**88.3**	**358.4**	-	-	-	-	-	-	**116.9**	-	-
	**Saturated fat (g)**	**Med per 100 g/mL**	-	-	-	-	-	-	-	-	-	-	-	**0.2**	**1.3**	-	-	-	-	-	-	**0.1**	-	-
		**IQR per 100 g/mL**	-	-	-	-	-	-	-	-	-	-	-	**0.2**	**0.1**	-	-	-	-	-	-	**0.1**	-	-
		**Med per RA**	-	-	-	-	-	-	-	-	-	-	-	**0.1**	**1.1**	-	-	-	-	-	-	**0.2**	-	-
		**IQR per RA**	-	-	-	-	-	-	-	-	-	-	-	**0.1**	**0.1**	-	-	-	-	-	-	**0.1**	-	-
	**Total sugars (g)**	**Med per 100 g/mL**	-	-	-	-	-	-	-	-	-	-	-	**1.0**	**1.3**	-	-	-	-	-	-	**0.4**	-	-
		**IQR per 100 g/mL**	-	-	-	-	-	-	-	-	-	-	-	**1.6**	**1.3**	-	-	-	-	-	-	**0.0**	-	-
		**Med per RA**	-	-	-	-	-	-	-	-	-	-	-	**0.5**	**1.1**	-	-	-	-	-	-	**0.5**	-	-
		**IQR per RA**	-	-	-	-	-	-	-	-	-	-	-	**0.3**	**1.1**	-	-	-	-	-	-	**0.0**	-	-
**Meat, poultry, their products and substitutes (L)**		**n**	**0**	**0**	**0**	**0**	**0**	**0**	**0**	**0**	**0**	**0**	**0**	**253**	**126**	**0**	**0**	**0**	**0**	**0**	**0**	**105**	**0**	**0**
	**Calories (kcal)**	**Med per 100 g/mL**	-	-	-	-	-	-	-	-	-	-	-	**200.0**	**214.3**	-	-	-	-	-	-	**210.0**	-	-
		**IQR per 100 g/mL**	-	-	-	-	-	-	-	-	-	-	-	**110.0**	**170.2**	-	-	-	-	-	-	**115.6**	-	-
		**Med per RA**	-	-	-	-	-	-	-	-	-	-	-	**165.0**	**127.0**	-	-	-	-	-	-	**165.0**	-	-
		**IQR per RA**	-	-	-	-	-	-	-	-	-	-	-	**111.0**	**129.9**	-	-	-	-	-	-	**113.2**	-	-
	**Sodium (mg)**	**Med per 100 g/mL**	-	-	-	-	-	-	-	-	-	-	-	**471.0**	**829.3**	-	-	-	-	-	-	**529.4**	-	-
		**IQR per 100 g/mL**	-	-	-	-	-	-	-	-	-	-	-	**410.0**	**296.4**	-	-	-	-	-	-	**413.6**	-	-
		**Med per RA**	-	-	-	-	-	-	-	-	-	-	-	**412.5**	**463.4**	-	-	-	-	-	-	**418.0**	-	-
		**IQR per RA**	-	-	-	-	-	-	-	-	-	-	-	**195.0**	**132.5**	-	-	-	-	-	-	**150.0**	-	-
	**Saturated fat (g)**	**Med per 100 g/mL**	-	-	-	-	-	-	-	-	-	-	-	**3.5**	**3.0**	-	-	-	-	-	-	**3.6**	-	-
		**IQR per 100 g/mL**	-	-	-	-	-	-	-	-	-	-	-	**5.5**	**7.8**	-	-	-	-	-	-	**6.5**	-	-
		**Med per RA**	-	-	-	-	-	-	-	-	-	-	-	**2.9**	**2.4**	-	-	-	-	-	-	**3.0**	-	-
		**IQR per RA**	-	-	-	-	-	-	-	-	-	-	-	**3.8**	**4.6**	-	-	-	-	-	-	**3.6**	-	-
	**Total sugars (g)**	**Med per 100 g/mL**	-	-	-	-	-	-	-	-	-	-	-	**0.0**	**0.0**	-	-	-	-	-	-	**0.0**	-	-
		**IQR per 100 g/mL**	-	-	-	-	-	-	-	-	-	-	-	**2.0**	**2.0**	-	-	-	-	-	-	**1.9**	-	-
		**Med per RA**	-	-	-	-	-	-	-	-	-	-	-	**0.0**	**0.0**	-	-	-	-	-	-	**0.0**	-	-
		**IQR per RA**	-	-	-	-	-	-	-	-	-	-	-	**1.5**	**1.2**	-	-	-	-	-	-	**1.7**	-	-
**Miscellaneous products (M)**		**n**	**0**	**0**	**0**	**0**	**2**	**0**	**0**	**44**	**1**	**1**	**11**	**81**	**1**	**7**	**5**	**0**	**0**	**17**	**0**	**44**	**0**	**1**
	**Calories (kcal)**	**Med per 100 g/mL**	-	-	-	-	**100.0**	-	-	**316.3**	**357.1**	**370.4**	**333.3**	**333.3**	**437.5**	**267.6**	**294.1**	-	-	**245.9**	-	**294.9**	-	**403.2**
		**IQR per 100 g/mL**	-	-	-	-	**0.0**	-	-	**91.8**	**0.0**	**0.0**	**79.1**	**202.4**	**0.0**	**144.6**	**8.4**	-	-	**67.3**	-	**184.3**	-	**0.0**
		**Med per RA**	-	-	-	-	**1.0**	-	-	**86.8**	**107.1**	**111.1**	**100.0**	**69.2**	**30.6**	**80.3**	**2.9**	-	-	**73.8**	-	**70.9**	-	**4.0**
		**IQR per RA**	-	-	-	-	**0.0**	-	-	**98.9**	**0.0**	**0.0**	**97.2**	**90.0**	**0.0**	**52.4**	**0.1**	-	-	**20.2**	-	**96.1**	-	**0.0**
	**Sodium (mg)**	**Med per 100 g/mL**	-	-	-	-	**27500.0**	-	-	**366.7**	**785.7**	**629.6**	**2466.7**	**344.6**	**2125.0**	**338.0**	**8470.6**	-	-	**396.8**	-	**353.7**	-	**4838.7**

Food category[1]			A. Lassonde	Agropur	Campbell	Canada Bread	Canada Dry Mott's	Coca-Cola	Danone	General Mills	George Weston	Kellogg	Kraft Heinz	Loblaw	Maple Leaf Foods	Mondelez	Nestlé	Ocean Spray	Parmalat	PepsiCo	Saputo	Sobeys	Sun-Rype	Unilever
		IQR per 100 g/mL	-	-	-	-	500.0	-	-	4369.3	0.0	0.0	2348.5	877.2	0.0	293.6	1934.6	-	-	115.5	-	2919.8	-	0.0
		Med per RA	-	-	-	-	275.0	-	-	92.9	235.7	188.9	576.9	88.9	148.8	101.4	84.7	-	-	119.0	-	96.9	-	48.4
		IQR per RA	-	-	-	-	5.0	-	-	38.2	0.0	0.0	785.0	86.8	0.0	88.1	19.3	-	-	34.6	-	73.6	-	0.0
	Saturated fat (g)	Med per 100 g/mL	-	-	-	-	0.0	-	-	2.2	1.4	0.0	0.0	2.4	12.5	0.0	0.0	-	-	1.1	-	2.5	-	3.2
		IQR per 100 g/mL	-	-	-	-	0.0	-	-	3.4	0.0	0.0	0.0	7.5	0.0	0.8	0.0	-	-	0.9	-	4.4	-	0.0
		Med per RA	-	-	-	-	0.0	-	-	0.7	0.4	0.0	0.0	0.4	0.9	0.0	0.0	-	-	0.3	-	0.7	-	0.0
		IQR per RA	-	-	-	-	0.0	-	-	1.0	0.0	0.0	0.0	1.3	0.0	0.2	0.0	-	-	0.3	-	1.3	-	0.0
	Total sugars (g)	Med per 100 g/mL	-	-	-	-	0.0	-	-	20.0	0.0	11.1	0.0	7.8	0.0	18.3	14.3	-	-	23.0	-	13.4	-	32.3
		IQR per 100 g/mL	-	-	-	-	0.0	-	-	31.2	0.0	0.0	16.5	23.8	0.0	23.5	10.5	-	-	8.8	-	23.8	-	0.0
		Med per RA	-	-	-	-	0.0	-	-	6.0	0.0	3.3	0.0	1.4	0.0	5.5	0.1	-	-	6.9	-	1.9	-	0.3
		IQR per RA	-	-	-	-	0.0	-	-	10.6	0.0	0.0	1.6	6.9	0.0	7.1	0.1	-	-	2.7	-	6.3	-	0.0
Baking/cooking ingredients (M1, M3, M5, M8, M11, M12)		n	0	0	0	0	0	0	0	30	1	1	11	62	1	7	0	0	0	17	0	34	0	0
	Calories (kcal)	Med per 100 g/mL	-	-	-	-	-	-	-	307.9	357.1	370.4	333.3	357.8	437.5	267.6	-	-	-	245.9	-	294.9	-	-
		IQR per 100 g/mL	-	-	-	-	-	-	-	98.9	0.0	0.0	79.1	152.4	0.0	144.6	-	-	-	67.3	-	167.9	-	-
		Med per RA	-	-	-	-	-	-	-	92.4	107.1	111.1	100.0	88.5	30.6	80.3	-	-	-	73.8	-	86.8	-	-
		IQR per RA	-	-	-	-	-	-	-	29.7	0.0	0.0	97.2	54.1	0.0	52.4	-	-	-	20.2	-	41.1	-	-
	Sodium (mg)	Med per 100 g/mL	-	-	-	-	-	-	-	347.4	785.7	629.6	2466.7	329.2	2125.0	338.0	-	-	-	396.8	-	330.1	-	-
		IQR per 100 g/mL	-	-	-	-	-	-	-	64.8	0.0	0.0	2348.5	215.7	0.0	293.6	-	-	-	115.5	-	309.1	-	-
		Med per RA	-	-	-	-	-	-	-	104.2	235.7	188.9	576.9	93.5	148.8	101.4	-	-	-	119.0	-	97.7	-	-
		IQR per RA	-	-	-	-	-	-	-	19.4	0.0	0.0	785.0	70.3	0.0	88.1	-	-	-	34.6	-	75.3	-	-
	Saturated fat (g)	Med per 100 g/mL	-	-	-	-	-	-	-	2.6	1.4	0.0	0.0	3.7	12.5	0.0	-	-	-	1.1	-	3.1	-	-
		IQR per 100 g/mL	-	-	-	-	-	-	-	2.5	0.0	0.0	0.0	7.4	0.0	0.8	-	-	-	0.9	-	4.2	-	-
		Med per RA	-	-	-	-	-	-	-	0.8	0.4	0.0	0.0	0.8	0.9	0.0	-	-	-	0.3	-	0.9	-	-
		IQR per RA	-	-	-	-	-	-	-	0.8	0.0	0.0	0.0	2.2	0.0	0.2	-	-	-	0.3	-	1.2	-	-
	Total sugars (g)	Med per 100 g/mL	-	-	-	-	-	-	-	32.8	0.0	11.1	0.0	19.5	0.0	18.3	-	-	-	23.0	-	17.8	-	-
		IQR per 100 g/mL	-	-	-	-	-	-	-	17.5	0.0	0.0	16.5	23.7	0.0	23.5	-	-	-	8.8	-	22.9	-	-
		Med per RA	-	-	-	-	-	-	-	9.8	0.0	3.3	0.0	5.0	0.0	5.5	-	-	-	6.9	-	5.0	-	-
		IQR per RA	-	-	-	-	-	-	-	5.3	0.0	0.0	1.6	6.9	0.0	7.1	-	-	-	2.7	-	5.7	-	-
Seasoning salts and mixes (M9, M10)		n	0	0	0	0	2	0	0	14	0	0	0	19	0	0	5	0	0	0	0	10	0	1
	Calories (kcal)	Med per 100 g/mL	-	-	-	-	100.0	-	-	333.3	-	-	-	250.0	-	-	294.1	-	-	-	-	142.9	-	403.2
		IQR per 100 g/mL	-	-	-	-	0.0	-	-	74.4	-	-	-	322.9	-	-	8.4	-	-	-	-	344.8	-	0.0
		Med per RA	-	-	-	-	1.0	-	-	3.3	-	-	-	2.0	-	-	2.9	-	-	-	-	1.4	-	4.0
		IQR per RA	-	-	-	-	0.0	-	-	0.7	-	-	-	2.9	-	-	0.1	-	-	-	-	3.4	-	0.0
	Sodium (mg)	Med per 100 g/mL	-	-	-	-	27500.0	-	-	5833.3	-	-	-	5000.0	-	-	8470.6	-	-	-	-	5983.5	-	4838.7
		IQR per 100 g/mL	-	-	-	-	500.0	-	-	2854.2	-	-	-	11155.0	-	-	1934.6	-	-	-	-	7031.3	-	0.0
		Med per RA	-	-	-	-	275.0	-	-	58.3	-	-	-	50.0	-	-	84.7	-	-	-	-	59.8	-	48.4
		IQR per RA	-	-	-	-	5.0	-	-	28.5	-	-	-	111.6	-	-	19.3	-	-	-	-	70.3	-	0.0
	Saturated fat (g)	Med per 100 g/mL	-	-	-	-	0.0	-	-	0.0	-	-	-	0.0	-	-	0.0	-	-	-	-	0.0	-	3.2
		IQR per 100 g/mL	-	-	-	-	0.0	-	-	0.0	-	-	-	0.0	-	-	0.0	-	-	-	-	0.0	-	0.0
		Med per RA	-	-	-	-	0.0	-	-	0.0	-	-	-	0.0	-	-	0.0	-	-	-	-	0.0	-	0.0
		IQR per RA	-	-	-	-	0.0	-	-	0.0	-	-	-	0.0	-	-	0.0	-	-	-	-	0.0	-	0.0
	Total sugars (g)	Med per 100 g/mL	-	-	-	-	0.0	-	-	0.0	-	-	-	0.0	-	-	14.3	-	-	-	-	0.0	-	32.3
		IQR per 100 g/mL	-	-	-	-	0.0	-	-	12.5	-	-	-	0.0	-	-	10.5	-	-	-	-	14.3	-	0.0
		Med per RA	-	-	-	-	0.0	-	-	0.0	-	-	-	0.0	-	-	0.1	-	-	-	-	0.0	-	0.3
		IQR per RA	-	-	-	-	0.0	-	-	0.1	-	-	-	0.0	-	-	0.1	-	-	-	-	0.1	-	0.0
**Combination dishes (N)**		**n**	**0**	**0**	**6**	**2**	**0**	**0**	**0**	**45**	**0**	**0**	**47**	**243**	**20**	**0**	**84**	**0**	**0**	**0**	**0**	**150**	**0**	**22**
	**Calories (kcal)**	**Med per 100 g/mL**	-	-	**102.2**	**246.8**	-	-	-	**185.5**	-	-	**126.7**	**162.5**	**299.3**	-	**203.8**	-	-	-	-	**183.9**	-	**110.1**
		**IQR per 100 g/mL**	-	-	**11.2**	**0.0**	-	-	-	**132.2**	-	-	**54.0**	**104.7**	**60.3**	-	**111.9**	-	-	-	-	**104.6**	-	**12.2**
		**Med per RA**	-	-	**306.5**	**493.5**	-	-	-	**462.3**	-	-	**336.6**	**360.4**	**584.2**	-	**410.0**	-	-	-	-	**362.4**	-	**330.3**
		**IQR per RA**	-	-	**33.5**	**0.0**	-	-	-	**160.0**	-	-	**206.7**	**150.2**	**129.3**	-	**118.8**	-	-	-	-	**155.2**	-	**36.6**
	**Sodium (mg)**	**Med per 100 g/mL**	-	-	**262.0**	**993.5**	-	-	-	**402.9**	-	-	**283.7**	**316.7**	**507.5**	-	**428.6**	-	-	-	-	**351.9**	-	**239.3**
		**IQR per 100 g/mL**	-	-	**32.6**	**19.5**	-	-	-	**478.4**	-	-	**50.5**	**165.6**	**230.8**	-	**271.5**	-	-	-	-	**183.0**	-	**25.8**
		**Med per RA**	-	-	**786.0**	**1987.0**	-	-	-	**924.1**	-	-	**848.8**	**738.5**	**1014.9**	-	**893.9**	-	-	-	-	**764.6**	-	**717.9**
		**IQR per RA**	-	-	**97.7**	**39.0**	-	-	-	**693.0**	-	-	**175.5**	**297.5**	**461.6**	-	**355.0**	-	-	-	-	**419.7**	-	**77.3**
	**Saturated fat (g)**	**Med per 100 g/mL**	-	-	**1.4**	**1.9**	-	-	-	**1.8**	-	-	**0.4**	**2.0**	**6.8**	-	**2.5**	-	-	-	-	**1.6**	-	**0.7**
		**IQR per 100 g/mL**	-	-	**0.9**	**0.0**	-	-	-	**1.8**	-	-	**0.7**	**2.9**	**3.6**	-	**2.3**	-	-	-	-	**2.4**	-	**0.6**
		**Med per RA**	-	-	**4.2**	**3.9**	-	-	-	**4.9**	-	-	**1.2**	**4.2**	**13.5**	-	**5.4**	-	-	-	-	**3.8**	-	**2.2**
		**IQR per RA**	-	-	**2.8**	**0.0**	-	-	-	**2.3**	-	-	**1.7**	**5.8**	**7.6**	-	**4.0**	-	-	-	-	**4.8**	-	**1.8**
	**Total sugars (g)**	**Med per 100 g/mL**	-	-	**2.8**	**1.9**	-	-	-	**2.8**	-	-	**4.5**	**2.5**	**5.5**	-	**2.4**	-	-	-	-	**2.7**	-	**3.0**
		**IQR per 100 g/mL**	-	-	**0.8**	**0.6**	-	-	-	**1.4**	-	-	**3.0**	**2.3**	**7.0**	-	**1.7**	-	-	-	-	**2.5**	-	**1.2**
		**Med per RA**	-	-	**8.4**	**3.9**	-	-	-	**7.1**	-	-	**13.4**	**5.4**	**10.9**	-	**5.4**	-	-	-	-	**5.7**	-	**8.9**
		**IQR per RA**	-	-	**2.5**	**1.3**	-	-	-	**5.1**	-	-	**9.1**	**5.4**	**14.1**	-	**3.5**	-	-	-	-	**4.9**	-	**3.5**
**Nuts and seeds (O)**		**n**	**0**	**0**	**0**	**0**	**0**	**0**	**0**	**0**	**0**	**0**	**11**	**44**	**0**	**0**	**0**	**0**	**0**	**0**	**0**	**28**	**0**	**0**
	**Calories (kcal)**	**Med per 100 g/mL**	-	-	-	-	-	-	-	-	-	-	**600.0**	**633.3**	-	-	-	-	-	-	-	**666.7**	-	-
		**IQR per 100 g/mL**	-	-	-	-	-	-	-	-	-	-	**66.7**	**75.0**	-	-	-	-	-	-	-	**60.0**	-	-
		**Med per RA**	-	-	-	-	-	-	-	-	-	-	**90.0**	**186.0**	-	-	-	-	-	-	-	**198.0**	-	-
		**IQR per RA**	-	-	-	-	-	-	-	-	-	-	**10.0**	**78.4**	-	-	-	-	-	-	-	**116.0**	-	-
	**Sodium (mg)**	**Med per 100 g/mL**	-	-	-	-	-	-	-	-	-	-	**400.0**	**0.0**	-	-	-	-	-	-	-	**0.0**	-	-
		**IQR per 100 g/mL**	-	-	-	-	-	-	-	-	-	-	**411.5**	**16.7**	-	-	-	-	-	-	-	**14.3**	-	-

Food category[1]			A. Lassonde	Agropur	Campbell	Canada Bread	Canada Dry Mott's	Coca-Cola	Danone	General Mills	George Weston	Kellogg	Kraft Heinz	Loblaw	Maple Leaf Foods	Mondelez	Nestlé	Ocean Spray	Parmalat	PepsiCo	Saputo	Sobeys	Sun-Rype	Unilever
		Med per RA	-	-	-	-	-	-	-	-	-	-	60.0	0.0	-	-	-	-	-	-	-	0.0	-	-
		IQR per RA	-	-	-	-	-	-	-	-	-	-	61.7	5.0	-	-	-	-	-	-	-	3.1	-	-
	Saturated fat (g)	Med per 100 g/mL	-	-	-	-	-	-	-	-	-	-	6.7	6.0	-	-	-	-	-	-	-	7.0	-	-
		IQR per 100 g/mL	-	-	-	-	-	-	-	-	-	-	2.2	3.0	-	-	-	-	-	-	-	3.0	-	-
		Med per RA	-	-	-	-	-	-	-	-	-	-	1.0	1.5	-	-	-	-	-	-	-	1.5	-	-
		IQR per RA	-	-	-	-	-	-	-	-	-	-	0.3	0.9	-	-	-	-	-	-	-	1.0	-	-
	Total sugars (g)	Med per 100 g/mL	-	-	-	-	-	-	-	-	-	-	6.7	4.0	-	-	-	-	-	-	-	4.0	-	-
		IQR per 100 g/mL	-	-	-	-	-	-	-	-	-	-	0.5	3.9	-	-	-	-	-	-	-	4.7	-	-
		Med per RA	-	-	-	-	-	-	-	-	-	-	1.0	1.0	-	-	-	-	-	-	-	1.0	-	-
		IQR per RA	-	-	-	-	-	-	-	-	-	-	0.1	0.4	-	-	-	-	-	-	-	0.6	-	-
Nuts, seeds and flours (O1, O4)		n	0	0	0	0	0	0	0	0	0	0	0	32	0	0	0	0	0	0	0	20	0	0
	Calories (kcal)	Med per 100 g/mL	-	-	-	-	-	-	-	-	-	-	-	633.3	-	-	-	-	-	-	-	720.0	-	-
		IQR per 100 g/mL	-	-	-	-	-	-	-	-	-	-	-	89.3	-	-	-	-	-	-	-	70.0	-	-
		Med per RA	-	-	-	-	-	-	-	-	-	-	-	190.0	-	-	-	-	-	-	-	216.0	-	-
		IQR per RA	-	-	-	-	-	-	-	-	-	-	-	26.8	-	-	-	-	-	-	-	21.0	-	-
	Sodium (mg)	Med per 100 g/mL	-	-	-	-	-	-	-	-	-	-	-	0.0	-	-	-	-	-	-	-	0.0	-	-
		IQR per 100 g/mL	-	-	-	-	-	-	-	-	-	-	-	16.7	-	-	-	-	-	-	-	2.0	-	-
		Med per RA	-	-	-	-	-	-	-	-	-	-	-	0.0	-	-	-	-	-	-	-	0.0	-	-
		IQR per RA	-	-	-	-	-	-	-	-	-	-	-	5.0	-	-	-	-	-	-	-	0.6	-	-
	Saturated fat (g)	Med per 100 g/mL	-	-	-	-	-	-	-	-	-	-	-	5.6	-	-	-	-	-	-	-	7.0	-	-
		IQR per 100 g/mL	-	-	-	-	-	-	-	-	-	-	-	3.0	-	-	-	-	-	-	-	3.3	-	-
		Med per RA	-	-	-	-	-	-	-	-	-	-	-	1.7	-	-	-	-	-	-	-	2.1	-	-
		IQR per RA	-	-	-	-	-	-	-	-	-	-	-	0.8	-	-	-	-	-	-	-	1.0	-	-
	Total sugars (g)	Med per 100 g/mL	-	-	-	-	-	-	-	-	-	-	-	3.3	-	-	-	-	-	-	-	2.0	-	-
		IQR per 100 g/mL	-	-	-	-	-	-	-	-	-	-	-	1.5	-	-	-	-	-	-	-	2.0	-	-
		Med per RA	-	-	-	-	-	-	-	-	-	-	-	1.0	-	-	-	-	-	-	-	0.6	-	-
		IQR per RA	-	-	-	-	-	-	-	-	-	-	-	0.5	-	-	-	-	-	-	-	0.6	-	-
Nut butters and pastes (O2, O3)		n	0	0	0	0	0	0	0	0	0	0	11	12	0	0	0	0	0	0	0	8	0	0
	Calories (kcal)	Med per 100 g/mL	-	-	-	-	-	-	-	-	-	-	600.0	600.0	-	-	-	-	-	-	-	666.7	-	-
		IQR per 100 g/mL	-	-	-	-	-	-	-	-	-	-	66.7	27.4	-	-	-	-	-	-	-	0.0	-	-
		Med per RA	-	-	-	-	-	-	-	-	-	-	90.0	90.0	-	-	-	-	-	-	-	100.0	-	-
		IQR per RA	-	-	-	-	-	-	-	-	-	-	10.0	8.6	-	-	-	-	-	-	-	0.0	-	-
	Sodium (mg)	Med per 100 g/mL	-	-	-	-	-	-	-	-	-	-	400.0	3.8	-	-	-	-	-	-	-	33.3	-	-
		IQR per 100 g/mL	-	-	-	-	-	-	-	-	-	-	411.5	83.3	-	-	-	-	-	-	-	275.0	-	-
		Med per RA	-	-	-	-	-	-	-	-	-	-	60.0	1.1	-	-	-	-	-	-	-	5.0	-	-
		IQR per RA	-	-	-	-	-	-	-	-	-	-	61.7	12.5	-	-	-	-	-	-	-	41.3	-	-
	Saturated fat (g)	Med per 100 g/mL	-	-	-	-	-	-	-	-	-	-	6.7	6.7	-	-	-	-	-	-	-	6.7	-	-
		IQR per 100 g/mL	-	-	-	-	-	-	-	-	-	-	2.2	2.6	-	-	-	-	-	-	-	3.3	-	-
		Med per RA	-	-	-	-	-	-	-	-	-	-	1.0	1.0	-	-	-	-	-	-	-	1.0	-	-
		IQR per RA	-	-	-	-	-	-	-	-	-	-	0.3	0.3	-	-	-	-	-	-	-	0.5	-	-
	Total sugars (g)	Med per 100 g/mL	-	-	-	-	-	-	-	-	-	-	6.7	6.7	-	-	-	-	-	-	-	6.7	-	-
		IQR per 100 g/mL	-	-	-	-	-	-	-	-	-	-	0.5	0.5	-	-	-	-	-	-	-	6.7	-	-
		Med per RA	-	-	-	-	-	-	-	-	-	-	1.0	1.0	-	-	-	-	-	-	-	1.0	-	-
		IQR per RA	-	-	-	-	-	-	-	-	-	-	0.1	0.1	-	-	-	-	-	-	-	1.0	-	-
**Potatoes, sweet potatoes and yams (P)**		**n**	0	0	0	0	0	0	0	7	0	0	0	23	0	0	0	0	0	0	0	23	0	0
	**Calories (kcal)**	**Med per 100 g/mL**	-	-	-	-	-	-	-	97.0	-	-	-	100.6	-	-	-	-	-	-	-	101.3	-	-
		**IQR per 100 g/mL**	-	-	-	-	-	-	-	11.4	-	-	-	62.4	-	-	-	-	-	-	-	67.7	-	-
		**Med per RA**	-	-	-	-	-	-	-	135.8	-	-	-	128.0	-	-	-	-	-	-	-	110.0	-	-
		**IQR per RA**	-	-	-	-	-	-	-	16.0	-	-	-	45.5	-	-	-	-	-	-	-	71.6	-	-
	**Sodium (mg)**	**Med per 100 g/mL**	-	-	-	-	-	-	-	315.6	-	-	-	200.0	-	-	-	-	-	-	-	88.3	-	-
		**IQR per 100 g/mL**	-	-	-	-	-	-	-	59.1	-	-	-	145.4	-	-	-	-	-	-	-	291.9	-	-
		**Med per RA**	-	-	-	-	-	-	-	441.9	-	-	-	297.5	-	-	-	-	-	-	-	147.5	-	-
		**IQR per RA**	-	-	-	-	-	-	-	82.7	-	-	-	259.5	-	-	-	-	-	-	-	345.9	-	-
	**Saturated fat (g)**	**Med per 100 g/mL**	-	-	-	-	-	-	-	0.6	-	-	-	0.4	-	-	-	-	-	-	-	0.2	-	-
		**IQR per 100 g/mL**	-	-	-	-	-	-	-	0.3	-	-	-	0.8	-	-	-	-	-	-	-	0.8	-	-
		**Med per RA**	-	-	-	-	-	-	-	0.9	-	-	-	0.4	-	-	-	-	-	-	-	0.2	-	-
		**IQR per RA**	-	-	-	-	-	-	-	0.4	-	-	-	1.2	-	-	-	-	-	-	-	1.1	-	-
	**Total sugars (g)**	**Med per 100 g/mL**	-	-	-	-	-	-	-	1.3	-	-	-	1.2	-	-	-	-	-	-	-	0.7	-	-
		**IQR per 100 g/mL**	-	-	-	-	-	-	-	0.4	-	-	-	1.8	-	-	-	-	-	-	-	1.4	-	-
		**Med per RA**	-	-	-	-	-	-	-	1.8	-	-	-	1.7	-	-	-	-	-	-	-	0.7	-	-
		**IQR per RA**	-	-	-	-	-	-	-	0.5	-	-	-	2.6	-	-	-	-	-	-	-	1.6	-	-
**Salads (Q)**		**n**	0	0	0	0	0	0	0	0	0	0	0	32	0	0	0	0	0	0	0	29	0	0
	**Calories (kcal)**	**Med per 100 g/mL**	-	-	-	-	-	-	-	-	-	-	-	128.0	-	-	-	-	-	-	-	90.0	-	-
		**IQR per 100 g/mL**	-	-	-	-	-	-	-	-	-	-	-	52.6	-	-	-	-	-	-	-	80.0	-	-
		**Med per RA**	-	-	-	-	-	-	-	-	-	-	-	130.1	-	-	-	-	-	-	-	90.0	-	-
		**IQR per RA**	-	-	-	-	-	-	-	-	-	-	-	86.1	-	-	-	-	-	-	-	80.0	-	-
	**Sodium (mg)**	**Med per 100 g/mL**	-	-	-	-	-	-	-	-	-	-	-	255.4	-	-	-	-	-	-	-	128.0	-	-
		**IQR per 100 g/mL**	-	-	-	-	-	-	-	-	-	-	-	197.3	-	-	-	-	-	-	-	234.0	-	-
		**Med per RA**	-	-	-	-	-	-	-	-	-	-	-	289.1	-	-	-	-	-	-	-	128.0	-	-

Food category[1]			A. Lassonde	Agropur	Campbell	Canada Bread	Canada Dry Mott's	Coca-Cola	Danone	General Mills	George Weston	Kellogg	Kraft Heinz	Loblaw	Maple Leaf Foods	Mondelez	Nestlé	Ocean Spray	Parmalat	PepsiCo	Saputo	Sobeys	Sun-Rype	Unilever
		IQR per RA	-	-	-	-	-	-	-	-	-	-	-	252.2	-	-	-	-	-	-	-	254.0	-	-
	Saturated fat (g)	Med per 100 g/mL	-	-	-	-	-	-	-	-	-	-	-	2.0	-	-	-	-	-	-	-	1.5	-	-
		IQR per 100 g/mL	-	-	-	-	-	-	-	-	-	-	-	1.9	-	-	-	-	-	-	-	1.3	-	-
		Med per RA	-	-	-	-	-	-	-	-	-	-	-	2.1	-	-	-	-	-	-	-	1.5	-	-
		IQR per RA	-	-	-	-	-	-	-	-	-	-	-	2.0	-	-	-	-	-	-	-	1.4	-	-
	Total sugars (g)	Med per 100 g/mL	-	-	-	-	-	-	-	-	-	-	-	2.5	-	-	-	-	-	-	-	2.0	-	-
		IQR per 100 g/mL	-	-	-	-	-	-	-	-	-	-	-	5.5	-	-	-	-	-	-	-	3.0	-	-
		Med per RA	-	-	-	-	-	-	-	-	-	-	-	3.2	-	-	-	-	-	-	-	2.0	-	-
		IQR per RA	-	-	-	-	-	-	-	-	-	-	-	6.9	-	-	-	-	-	-	-	3.0	-	-
Sauces, gravies, dressings and condiments (R)		n	0	0	21	0	0	0	0	7	0	0	80	258	1	0	3	2	0	19	4	83	0	19
	Calories (kcal)	Med per 100 g/mL	-	-	41.7	-	-	-	-	40.0	-	-	100.0	133.3	300.0	-	300.0	200.0	-	181.8	161.3	120.0	-	100.0
		IQR per 100 g/mL	-	-	38.7	-	-	-	-	8.3	-	-	80.7	136.0	0.0	-	133.3	0.0	-	152.2	0.0	144.0	-	145.8
		Med per RA	-	-	25.0	-	-	-	-	24.0	-	-	50.0	45.0	15.0	-	45.0	120.0	-	54.5	48.4	60.0	-	20.0
		IQR per RA	-	-	40.0	-	-	-	-	5.0	-	-	40.0	60.0	0.0	-	20.0	0.0	-	35.6	0.0	45.0	-	50.0
	Sodium (mg)	Med per 100 g/mL	-	-	375.0	-	-	-	-	720.0	-	-	708.3	450.0	1700.0	-	1733.3	16.7	-	500.0	548.4	516.7	-	483.3
		IQR per 100 g/mL	-	-	166.7	-	-	-	-	100.0	-	-	650.0	582.5	0.0	-	4100.0	0.0	-	287.2	16.1	491.3	-	466.7
		Med per RA	-	-	280.0	-	-	-	-	372.0	-	-	320.0	200.0	85.0	-	260.0	10.0	-	171.4	164.5	297.6	-	225.0
		IQR per RA	-	-	150.0	-	-	-	-	126.0	-	-	193.1	280.0	0.0	-	615.0	0.0	-	146.7	4.8	269.9	-	222.5
	Saturated fat (g)	Med per 100 g/mL	-	-	0.0	-	-	-	-	0.0	-	-	0.1	0.0	0.0	-	0.0	0.0	-	1.6	8.9	0.0	-	0.0
		IQR per 100 g/mL	-	-	0.8	-	-	-	-	0.0	-	-	1.3	1.7	0.0	-	0.0	0.0	-	3.6	1.6	0.8	-	1.0
		Med per RA	-	-	0.0	-	-	-	-	0.0	-	-	0.1	0.0	0.0	-	0.0	0.0	-	0.5	2.7	0.0	-	0.0
		IQR per RA	-	-	0.5	-	-	-	-	0.0	-	-	1.4	1.0	0.0	-	0.0	0.0	-	1.1	0.5	0.5	-	0.6
	Total sugars (g)	Med per 100 g/mL	-	-	1.7	-	-	-	-	4.0	-	-	6.5	5.0	40.0	-	40.0	40.0	-	0.0	6.5	5.0	-	1.7
		IQR per 100 g/mL	-	-	6.7	-	-	-	-	1.3	-	-	19.3	17.6	0.0	-	33.3	0.0	-	3.3	0.8	23.4	-	6.7
		Med per RA	-	-	1.0	-	-	-	-	2.4	-	-	4.5	3.0	2.0	-	6.0	24.0	-	0.0	1.9	3.6	-	1.0
		IQR per RA	-	-	4.0	-	-	-	-	0.0	-	-	5.0	5.0	0.0	-	5.0	0.0	-	2.0	0.2	7.0	-	2.0
Snacks (S)		n	0	0	0	3	0	0	0	7	0	17	0	145	8	6	1	1	0	109	0	79	0	0
	Calories (kcal)	Med per 100 g/mL	-	-	-	531.9	-	-	-	488.4	-	500.0	-	520.0	300.0	437.4	571.4	466.7	-	520.0	-	520.0	-	-
		IQR per 100 g/mL	-	-	-	21.3	-	-	-	75.1	-	35.7	-	117.8	147.1	5.2	0.0	0.0	-	40.0	-	100.0	-	-
		Med per RA	-	-	-	266.0	-	-	-	244.2	-	250.0	-	260.0	60.0	218.7	285.7	233.3	-	260.0	-	260.0	-	-
		IQR per RA	-	-	-	10.6	-	-	-	37.5	-	17.9	-	58.9	29.4	2.6	0.0	0.0	-	20.0	-	50.0	-	-
	Sodium (mg)	Med per 100 g/mL	-	-	-	574.5	-	-	-	595.2	-	642.9	-	460.0	1150.0	1217.4	392.9	166.7	-	660.0	-	480.0	-	-
		IQR per 100 g/mL	-	-	-	117.0	-	-	-	577.0	-	142.9	-	444.4	924.4	150.0	0.0	0.0	-	360.0	-	405.0	-	-
		Med per RA	-	-	-	287.2	-	-	-	297.6	-	321.4	-	220.0	230.0	608.7	196.4	83.3	-	330.0	-	240.0	-	-
		IQR per RA	-	-	-	58.5	-	-	-	288.5	-	71.4	-	193.7	184.9	75.0	0.0	0.0	-	180.0	-	202.5	-	-
	Saturated fat (g)	Med per 100 g/mL	-	-	-	8.5	-	-	-	4.8	-	8.9	-	4.4	9.5	2.0	7.1	6.7	-	3.0	-	4.0	-	-
		IQR per 100 g/mL	-	-	-	0.0	-	-	-	12.0	-	1.8	-	5.0	4.8	1.1	0.0	0.0	-	1.0	-	5.0	-	-
		Med per RA	-	-	-	4.3	-	-	-	2.4	-	4.5	-	2.0	1.9	1.0	3.6	3.3	-	1.5	-	2.0	-	-
		IQR per RA	-	-	-	0.0	-	-	-	6.0	-	0.9	-	2.4	1.0	0.5	0.0	0.0	-	0.5	-	2.5	-	-
	Total sugars (g)	Med per 100 g/mL	-	-	-	2.1	-	-	-	4.7	-	3.6	-	4.0	1.0	4.3	3.6	33.3	-	2.0	-	4.0	-	-
		IQR per 100 g/mL	-	-	-	0.0	-	-	-	4.7	-	0.0	-	6.0	2.4	4.5	0.0	0.0	-	2.0	-	4.0	-	-
		Med per RA	-	-	-	1.1	-	-	-	2.3	-	1.8	-	2.0	0.2	2.2	1.8	16.7	-	1.0	-	2.0	-	-
		IQR per RA	-	-	-	0.0	-	-	-	2.3	-	0.0	-	3.0	0.5	2.3	0.0	0.0	-	1.0	-	2.0	-	-
Soups (T)		n	0	0	135	0	0	0	0	0	0	0	0	66	0	0	0	0	0	0	0	41	0	51
	Calories (kcal)	Med per 100 g/mL	-	-	48.0	-	-	-	-	-	-	-	-	44.0	-	-	-	-	-	-	-	44.0	-	8.0
		IQR per 100 g/mL	-	-	30.0	-	-	-	-	-	-	-	-	50.0	-	-	-	-	-	-	-	70.0	-	24.6
		Med per RA	-	-	120.0	-	-	-	-	-	-	-	-	110.0	-	-	-	-	-	-	-	110.0	-	20.0
		IQR per RA	-	-	75.0	-	-	-	-	-	-	-	-	125.0	-	-	-	-	-	-	-	175.0	-	61.4
	Sodium (mg)	Med per 100 g/mL	-	-	272.0	-	-	-	-	-	-	-	-	270.2	-	-	-	-	-	-	-	244.0	-	293.3
		IQR per 100 g/mL	-	-	82.0	-	-	-	-	-	-	-	-	90.5	-	-	-	-	-	-	-	56.0	-	120.3
		Med per RA	-	-	680.0	-	-	-	-	-	-	-	-	675.5	-	-	-	-	-	-	-	610.0	-	733.3
		IQR per RA	-	-	205.0	-	-	-	-	-	-	-	-	226.3	-	-	-	-	-	-	-	140.0	-	300.6
	Saturated fat (g)	Med per 100 g/mL	-	-	0.4	-	-	-	-	-	-	-	-	0.2	-	-	-	-	-	-	-	0.2	-	0.0
		IQR per 100 g/mL	-	-	0.8	-	-	-	-	-	-	-	-	0.9	-	-	-	-	-	-	-	1.2	-	0.2
		Med per RA	-	-	1.0	-	-	-	-	-	-	-	-	0.5	-	-	-	-	-	-	-	0.4	-	0.0
		IQR per RA	-	-	1.9	-	-	-	-	-	-	-	-	2.3	-	-	-	-	-	-	-	3.0	-	0.5
	Total sugars (g)	Med per 100 g/mL	-	-	1.2	-	-	-	-	-	-	-	-	0.6	-	-	-	-	-	-	-	0.4	-	0.6
		IQR per 100 g/mL	-	-	1.6	-	-	-	-	-	-	-	-	1.2	-	-	-	-	-	-	-	1.6	-	0.8
		Med per RA	-	-	3.0	-	-	-	-	-	-	-	-	1.5	-	-	-	-	-	-	-	1.0	-	1.4
		IQR per RA	-	-	4.0	-	-	-	-	-	-	-	-	3.1	-	-	-	-	-	-	-	4.0	-	2.0
Sugars and sweets (U)		n	0	0	0	0	4	0	0	17	0	0	8	132	0	49	32	0	0	3	0	55	15	0
	Calories (kcal)	Med per 100 g/mL	-	-	-	-	300.0	-	-	347.8	-	-	439.5	348.1	-	523.8	516.1	-	-	355.6	-	350.0	324.3	-
		IQR per 100 g/mL	-	-	-	-	95.8	-	-	33.1	-	-	100.0	166.7	-	57.6	64.6	-	-	111.1	-	66.7	32.8	-
		Med per RA	-	-	-	-	50.0	-	-	139.1	-	-	75.0	103.6	-	200.0	206.5	-	-	213.3	-	96.4	64.9	-
		IQR per RA	-	-	-	-	8.8	-	-	13.3	-	-	18.0	130.0	-	38.0	25.8	-	-	66.7	-	80.0	6.6	-
	Sodium (mg)	Med per 100 g/mL	-	-	-	-	50.0	-	-	195.7	-	-	7.1	33.3	-	76.9	87.9	-	-	33.3	-	8.3	35.7	-
		IQR per 100 g/mL	-	-	-	-	48.3	-	-	85.9	-	-	78.6	75.0	-	67.2	45.5	-	-	16.7	-	45.8	62.3	-
		Med per RA	-	-	-	-	10.0	-	-	78.3	-	-	1.1	5.0	-	30.8	35.1	-	-	20.0	-	5.0	7.1	-
		IQR per RA	-	-	-	-	3.3	-	-	34.4	-	-	15.5	25.2	-	26.9	17.4	-	-	10.0	-	15.4	12.5	-

Food category[1]			A. Lassonde	Agropur	Campbell	Canada Bread	Canada Dry Mott's	Coca-Cola	Danone	General Mills	George Weston	Kellogg	Kraft Heinz	Loblaw	Maple Leaf Foods	Mondelez	Nestlé	Ocean Spray	Parmalat	PepsiCo	Saputo	Sobeys	Sun-Rype	Unilever
	**Saturated fat (g)**	**Med per 100 g/mL**	-	-	-	-	0.0	-	-	2.2	-	-	12.0	0.0	-	16.3	14.6	-	-	0.0	-	0.0	0.0	-
		**IQR per 100 g/mL**	-	-	-	-	0.0	-	-	2.4	-	-	21.4	10.1	-	8.4	6.7	-	-	0.0	-	0.0	0.0	-
		**Med per RA**	-	-	-	-	0.0	-	-	0.9	-	-	2.6	0.0	-	6.3	5.8	-	-	0.0	-	0.0	0.0	-
		**IQR per RA**	-	-	-	-	0.0	-	-	1.0	-	-	3.2	2.0	-	4.2	2.7	-	-	0.0	-	0.0	0.0	-
	**Total sugars (g)**	**Med per 100 g/mL**	-	-	-	-	76.7	-	-	43.5	-	-	57.0	56.9	-	50.0	53.9	-	-	53.3	-	60.0	78.4	-
		**IQR per 100 g/mL**	-	-	-	-	28.3	-	-	8.5	-	-	30.0	19.3	-	8.9	12.0	-	-	11.1	-	31.7	0.2	-
		**Med per RA**	-	-	-	-	13.0	-	-	17.4	-	-	11.1	15.3	-	20.0	21.4	-	-	32.0	-	16.2	15.7	-
		**IQR per RA**	-	-	-	-	2.5	-	-	3.4	-	-	7.6	11.5	-	3.6	3.9	-	-	6.7	-	10.8	0.0	-
Confectionary (U1, U3, U4, U10, U11)		n	0	0	0	0	0	0	0	17	0	0	6	56	0	49	28	0	0	0	0	30	15	0
	Calories (kcal)	Med per 100 g/mL	-	-	-	-	-	-	-	347.8	-	-	482.1	484.4	-	523.8	516.1	-	-	-	-	350.7	324.3	-
		IQR per 100 g/mL	-	-	-	-	-	-	-	33.1	-	-	73.0	195.8	-	57.6	36.1	-	-	-	-	206.3	32.8	-
		Med per RA	-	-	-	-	-	-	-	139.1	-	-	77.7	140.0	-	200.0	206.5	-	-	-	-	133.3	64.9	-
		IQR per RA	-	-	-	-	-	-	-	13.3	-	-	20.1	103.6	-	38.0	14.4	-	-	-	-	51.2	6.6	-
	Sodium (mg)	Med per 100 g/mL	-	-	-	-	-	-	-	195.7	-	-	42.9	66.7	-	76.9	86.4	-	-	-	-	31.3	35.7	-
		IQR per 100 g/mL	-	-	-	-	-	-	-	85.9	-	-	89.3	46.2	-	67.2	34.1	-	-	-	-	63.5	62.3	-
		Med per RA	-	-	-	-	-	-	-	78.3	-	-	6.4	22.5	-	30.8	34.6	-	-	-	-	12.5	7.1	-
		IQR per RA	-	-	-	-	-	-	-	34.4	-	-	24.6	23.3	-	26.9	13.6	-	-	-	-	21.4	12.5	-
	Saturated fat (g)	Med per 100 g/mL	-	-	-	-	-	-	-	2.2	-	-	19.6	11.9	-	16.3	15.3	-	-	-	-	0.0	0.0	-
		IQR per 100 g/mL	-	-	-	-	-	-	-	2.4	-	-	12.4	16.9	-	8.4	5.1	-	-	-	-	16.3	0.0	-
		Med per RA	-	-	-	-	-	-	-	0.9	-	-	2.9	2.5	-	6.3	6.1	-	-	-	-	0.0	0.0	-
		IQR per RA	-	-	-	-	-	-	-	1.0	-	-	0.7	6.6	-	4.2	2.0	-	-	-	-	2.6	0.0	-
	Total sugars (g)	Med per 100 g/mL	-	-	-	-	-	-	-	43.5	-	-	49.9	51.3	-	50.0	52.1	-	-	-	-	51.3	78.4	-
		IQR per 100 g/mL	-	-	-	-	-	-	-	8.5	-	-	28.5	12.7	-	8.9	10.3	-	-	-	-	13.8	0.2	-
		Med per RA	-	-	-	-	-	-	-	17.4	-	-	8.0	19.0	-	20.0	20.8	-	-	-	-	18.6	15.7	-
		IQR per RA	-	-	-	-	-	-	-	3.4	-	-	9.7	5.3	-	3.6	4.1	-	-	-	-	5.3	0.0	-
Sugars and syrups (U8, U9, U12, U14, U15)		n	0	0	0	0	4	0	0	0	0	0	2	76	0	0	4	0	0	3	0	25	0	0
	Calories (kcal)	Med per 100 g/mL	-	-	-	-	300.0	-	-	-	-	-	400.0	333.3	-	-	333.3	-	-	355.6	-	333.3	-	-
		IQR per 100 g/mL	-	-	-	-	95.8	-	-	-	-	-	0.0	100.0	-	-	16.7	-	-	111.1	-	41.7	-	-
		Med per RA	-	-	-	-	50.0	-	-	-	-	-	60.0	60.0	-	-	100.0	-	-	213.3	-	50.0	-	-
		IQR per RA	-	-	-	-	8.8	-	-	-	-	-	0.0	72.0	-	-	5.0	-	-	66.7	-	16.7	-	-
	Sodium (mg)	Med per 100 g/mL	-	-	-	-	50.0	-	-	-	-	-	0.0	0.0	-	-	166.7	-	-	33.3	-	0.0	-	-
		IQR per 100 g/mL	-	-	-	-	48.3	-	-	-	-	-	0.0	33.3	-	-	41.7	-	-	16.7	-	25.0	-	-
		Med per RA	-	-	-	-	10.0	-	-	-	-	-	0.0	0.0	-	-	50.0	-	-	20.0	-	0.0	-	-
		IQR per RA	-	-	-	-	3.3	-	-	-	-	-	0.0	5.0	-	-	12.5	-	-	10.0	-	5.0	-	-
	Saturated fat (g)	Med per 100 g/mL	-	-	-	-	0.0	-	-	-	-	-	0.0	0.0	-	-	0.0	-	-	0.0	-	0.0	-	-
		IQR per 100 g/mL	-	-	-	-	0.0	-	-	-	-	-	0.0	0.0	-	-	0.0	-	-	0.0	-	0.0	-	-
		Med per RA	-	-	-	-	0.0	-	-	-	-	-	0.0	0.0	-	-	0.0	-	-	0.0	-	0.0	-	-
		IQR per RA	-	-	-	-	0.0	-	-	-	-	-	0.0	0.0	-	-	0.0	-	-	0.0	-	0.0	-	-
	Total sugars (g)	Med per 100 g/mL	-	-	-	-	76.7	-	-	-	-	-	86.7	65.0	-	-	73.3	-	-	53.3	-	78.3	-	-
		IQR per 100 g/mL	-	-	-	-	28.3	-	-	-	-	-	0.0	26.7	-	-	5.0	-	-	11.1	-	27.6	-	-
		Med per RA	-	-	-	-	13.0	-	-	-	-	-	13.0	11.5	-	-	22.0	-	-	32.0	-	12.0	-	-
		IQR per RA	-	-	-	-	2.5	-	-	-	-	-	0.0	8.3	-	-	1.5	-	-	6.7	-	8.2	-	-
**Vegetables (V)**		**n**	1	0	6	0	8	0	0	9	0	0	3	200	0	0	0	0	0	0	0	106	0	4
	**Calories (kcal)**	**Med per 100 g/mL**	24.0	-	26.0	-	24.0	-	-	20.0	-	-	33.3	35.3	-	-	-	-	-	-	-	29.7	-	33.3
		**IQR per 100 g/mL**	0.0	-	16.9	-	0.0	-	-	18.9	-	-	40.0	46.6	-	-	-	-	-	-	-	62.4	-	12.5
		**Med per RA**	60.0	-	55.0	-	60.0	-	-	21.6	-	-	20.0	29.4	-	-	-	-	-	-	-	25.3	-	10.0
		**IQR per RA**	0.0	-	17.5	-	0.0	-	-	10.0	-	-	17.5	23.6	-	-	-	-	-	-	-	19.8	-	3.8
	**Sodium (mg)**	**Med per 100 g/mL**	168.0	-	72.2	-	248.0	-	-	144.0	-	-	192.0	104.0	-	-	-	-	-	-	-	124.0	-	733.3
		**IQR per 100 g/mL**	0.0	-	96.6	-	61.0	-	-	32.0	-	-	320.8	367.6	-	-	-	-	-	-	-	373.4	-	108.3
		**Med per RA**	420.0	-	152.5	-	620.0	-	-	170.0	-	-	100.0	102.9	-	-	-	-	-	-	-	127.5	-	220.0
		**IQR per RA**	0.0	-	245.0	-	152.5	-	-	87.8	-	-	232.5	195.2	-	-	-	-	-	-	-	228.8	-	32.5
	**Saturated fat (g)**	**Med per 100 g/mL**	0.0	-	0.0	-	0.0	-	-	0.0	-	-	0.0	0.0	-	-	-	-	-	-	-	0.0	-	0.0
		**IQR per 100 g/mL**	0.0	-	0.0	-	0.0	-	-	0.0	-	-	0.0	0.0	-	-	-	-	-	-	-	0.0	-	0.0
		**Med per RA**	0.0	-	0.0	-	0.0	-	-	0.0	-	-	0.0	0.0	-	-	-	-	-	-	-	0.0	-	0.0
		**IQR per RA**	0.0	-	0.0	-	0.0	-	-	0.0	-	-	0.0	0.0	-	-	-	-	-	-	-	0.0	-	0.0
	**Total sugars (g)**	**Med per 100 g/mL**	2.4	-	4.4	-	4.2	-	-	2.0	-	-	5.0	2.1	-	-	-	-	-	-	-	2.4	-	6.7
		**IQR per 100 g/mL**	0.0	-	2.4	-	0.5	-	-	1.6	-	-	8.8	3.5	-	-	-	-	-	-	-	3.2	-	1.7
		**Med per RA**	6.0	-	8.5	-	10.5	-	-	1.7	-	-	3.0	2.0	-	-	-	-	-	-	-	2.0	-	2.0
		**IQR per RA**	0.0	-	4.8	-	1.3	-	-	1.4	-	-	1.5	3.0	-	-	-	-	-	-	-	3.0	-	0.5
Vegetables without sauce (V1, V3-V5, V8)		n	0	0	1	0	0	0	0	7	0	0	0	145	0	0	0	0	0	0	0	77	0	0
	Calories (kcal)	Med per 100 g/mL	-	-	41.2	-	-	-	-	20.0	-	-	-	35.3	-	-	-	-	-	-	-	29.4	-	-
		IQR per 100 g/mL	-	-	0.0	-	-	-	-	13.4	-	-	-	46.6	-	-	-	-	-	-	-	32.5	-	-
		Med per RA	-	-	35.0	-	-	-	-	25.0	-	-	-	30.0	-	-	-	-	-	-	-	25.0	-	-
		IQR per RA	-	-	0.0	-	-	-	-	14.9	-	-	-	20.0	-	-	-	-	-	-	-	12.9	-	-
	Sodium (mg)	Med per 100 g/mL	-	-	76.5	-	-	-	-	136.0	-	-	-	70.6	-	-	-	-	-	-	-	76.5	-	-
		IQR per 100 g/mL	-	-	0.0	-	-	-	-	102.0	-	-	-	168.0	-	-	-	-	-	-	-	212.2	-	-
		Med per RA	-	-	65.0	-	-	-	-	170.0	-	-	-	55.6	-	-	-	-	-	-	-	65.0	-	-
		IQR per RA	-	-	0.0	-	-	-	-	105.4	-	-	-	135.0	-	-	-	-	-	-	-	210.0	-	-
	Saturated fat	Med per 100 g/mL	-	-	0.0	-	-	-	-	0.0	-	-	-	0.0	-	-	-	-	-	-	-	0.0	-	-

Food category[1]			A. Lassonde	Agropur	Campbell	Canada Bread	Canada Dry Mott's	Coca-Cola	Danone	General Mills	George Weston	Kellogg	Kraft Heinz	Loblaw	Maple Leaf Foods	Mondelez	Nestlé	Ocean Spray	Parmalat	PepsiCo	Saputo	Sobeys	Sun-Rype	Unilever
	(g)	IQR per 100 g/mL	-	-	0.0	-	-	-	-	0.0	-	-	-	0.0	-	-	-	-	-	-	-	0.0	-	-
		Med per RA	-	-	0.0	-	-	-	-	0.0	-	-	-	0.0	-	-	-	-	-	-	-	0.0	-	-
		IQR per RA	-	-	0.0	-	-	-	-	0.0	-	-	-	0.0	-	-	-	-	-	-	-	0.0	-	-
	Total sugars	Med per 100 g/mL	-	-	5.9	-	-	-	-	2.0	-	-	-	1.6	-	-	-	-	-	-	-	2.0	-	-
	(g)	IQR per 100 g/mL	-	-	0.0	-	-	-	-	1.4	-	-	-	3.5	-	-	-	-	-	-	-	2.4	-	-
		Med per RA	-	-	5.0	-	-	-	-	1.7	-	-	-	2.0	-	-	-	-	-	-	-	2.0	-	-
		IQR per RA	-	-	0.0	-	-	-	-	1.4	-	-	-	3.0	-	-	-	-	-	-	-	2.0	-	-
Vegetables with		n	0	0	0	0	0	0	0	2	0	0	2	45	0	0	0	0	0	0	0	21	0	4
sauce, pastes (V2,	Calories	Med per 100 g/mL	-	-	-	-	-	-	-	36.0	-	-	66.7	40.0	-	-	-	-	-	-	-	83.3	-	33.3
V9-V12)	(kcal)	IQR per 100 g/mL	-	-	-	-	-	-	-	0.0	-	-	33.3	66.5	-	-	-	-	-	-	-	108.3	-	12.5
		Med per RA	-	-	-	-	-	-	-	21.6	-	-	17.5	20.0	-	-	-	-	-	-	-	20.0	-	10.0
		IQR per RA	-	-	-	-	-	-	-	0.0	-	-	2.5	28.4	-	-	-	-	-	-	-	23.0	-	3.8
	Sodium (mg)	Med per 100 g/mL	-	-	-	-	-	-	-	144.0	-	-	345.8	571.4	-	-	-	-	-	-	-	500.0	-	733.3
		IQR per 100 g/mL	-	-	-	-	-	-	-	0.0	-	-	320.8	533.3	-	-	-	-	-	-	-	400.0	-	108.3
		Med per RA	-	-	-	-	-	-	-	86.4	-	-	57.5	193.3	-	-	-	-	-	-	-	150.0	-	220.0
		IQR per RA	-	-	-	-	-	-	-	0.0	-	-	42.5	143.0	-	-	-	-	-	-	-	145.0	-	32.5
	Saturated fat	Med per 100 g/mL	-	-	-	-	-	-	-	0.0	-	-	0.0	0.0	-	-	-	-	-	-	-	0.0	-	0.0
	(g)	IQR per 100 g/mL	-	-	-	-	-	-	-	0.0	-	-	0.0	0.5	-	-	-	-	-	-	-	0.0	-	0.0
		Med per RA	-	-	-	-	-	-	-	0.0	-	-	0.0	0.0	-	-	-	-	-	-	-	0.0	-	0.0
		IQR per RA	-	-	-	-	-	-	-	0.0	-	-	0.0	0.2	-	-	-	-	-	-	-	0.0	-	0.0
	Total sugars	Med per 100 g/mL	-	-	-	-	-	-	-	4.0	-	-	12.5	1.5	-	-	-	-	-	-	-	10.0	-	6.7
	(g)	IQR per 100 g/mL	-	-	-	-	-	-	-	0.0	-	-	7.5	5.0	-	-	-	-	-	-	-	20.0	-	1.7
		Med per RA	-	-	-	-	-	-	-	2.4	-	-	3.0	0.7	-	-	-	-	-	-	-	3.0	-	2.0
		IQR per RA	-	-	-	-	-	-	-	0.0	-	-	0.0	3.0	-	-	-	-	-	-	-	6.0	-	0.5
Vegetable juice		n	1	0	5	0	8	0	0	0	0	0	1	10	0	0	0	0	0	0	0	8	0	0
and vegetable	Calories	Med per 100 g/mL	24.0	-	24.0	-	24.0	-	-	-	-	-	20.0	24.0	-	-	-	-	-	-	-	20.0	-	-
drink (V7)	(kcal)	IQR per 100 g/mL	0.0	-	8.0	-	0.0	-	-	-	-	-	0.0	0.0	-	-	-	-	-	-	-	8.0	-	-
		Med per RA	60.0	-	60.0	-	60.0	-	-	-	-	-	50.0	60.0	-	-	-	-	-	-	-	50.0	-	-
		IQR per RA	0.0	-	20.0	-	0.0	-	-	-	-	-	0.0	0.0	-	-	-	-	-	-	-	20.0	-	-
	Sodium (mg)	Med per 100 g/mL	168.0	-	68.0	-	248.0	-	-	-	-	-	192.0	174.0	-	-	-	-	-	-	-	204.0	-	-
		IQR per 100 g/mL	0.0	-	126.0	-	61.0	-	-	-	-	-	0.0	114.5	-	-	-	-	-	-	-	136.0	-	-
		Med per RA	420.0	-	170.0	-	620.0	-	-	-	-	-	480.0	435.0	-	-	-	-	-	-	-	510.0	-	-
		IQR per RA	0.0	-	315.0	-	152.5	-	-	-	-	-	0.0	286.3	-	-	-	-	-	-	-	340.0	-	-
	Saturated fat	Med per 100 g/mL	0.0	-	0.0	-	0.0	-	-	-	-	-	0.0	0.0	-	-	-	-	-	-	-	0.0	-	-
	(g)	IQR per 100 g/mL	0.0	-	0.0	-	0.0	-	-	-	-	-	0.0	0.0	-	-	-	-	-	-	-	0.0	-	-
		Med per RA	0.0	-	0.0	-	0.0	-	-	-	-	-	0.0	0.0	-	-	-	-	-	-	-	0.0	-	-
		IQR per RA	0.0	-	0.0	-	0.0	-	-	-	-	-	0.0	0.0	-	-	-	-	-	-	-	0.0	-	-
	Total sugars	Med per 100 g/mL	2.4	-	3.6	-	4.2	-	-	-	-	-	2.4	3.2	-	-	-	-	-	-	-	3.2	-	-
	(g)	IQR per 100 g/mL	0.0	-	2.0	-	0.5	-	-	-	-	-	0.0	1.4	-	-	-	-	-	-	-	1.4	-	-
		Med per RA	6.0	-	9.0	-	10.5	-	-	-	-	-	6.0	8.0	-	-	-	-	-	-	-	8.0	-	-
		IQR per RA	0.0	-	5.0	-	1.3	-	-	-	-	-	0.0	3.5	-	-	-	-	-	-	-	3.5	-	-

[1]Food categories are based on those defined in Health Canada's Table of Reference Amounts for Foods (TRA), listed in brackets. Values at the TRA major food category level are shown in bold. TRA minor food categories that were combined are indicated in brackets. If none of the sampled products fell into that food category, it was omitted from the table.

[2]The number of products offered by the company in that food category (that were included in the FLIP 2017 database). Dashes indicate that the company did not offer products within that food category.

[3]Med: Median

[4]IQR: Interquartile range

[5]Reference amount as defined in Health Canada's Table of Reference Amounts for Food.

Supplementary Table 4. The number and percentage of products offered by each company that exceeded Health Canada's proposed "high in" front-of-package labelling thresholds for saturated fat, sodium and/or total sugars, presented by food category.

Food category[1]			A. Lassonde	Agropur	Campbell	Canada Bread	Canada Dry Mott's	Coca-Cola	Danone	General Mills	George Weston	Kellogg	Kraft Heinz	Loblaw	Maple Leaf Foods	Mondelēz	Nestlé	Ocean Spray	Parmalat	PepsiCo	Saputo	Sobeys	Sun-Rype	Unilever
A. Bakery products	Total no. of products[2]		0	1	24	90	0	0	4	97	164	72	0	456	0	164	1	0	0	50	0	333	0	0
	Saturated fat	n[3]	-	0	8	21	-	-	2	46	11	16	0	173	-	43	1	-	-	9	0	171	-	-
		%[4]	-	0.0	29.6	23.3	-	-	50.0	47.4	6.7	22,2	0.0	37.9	-	26.2	100.0	-	-	18.0	0.0	51.4	-	-
	Sodium	n	-	0	13	10	-	-	0	20	77	21	0	89	-	24	0	-	-	11	0	111	-	-
		%	-	0.0	48.1	11.1	-	-	0.0	20.6	47.0	29.2	0.0	19.5	-	14.6	0.0	-	-	22.0	0.0	33.3	-	-
	Total sugars	n	-	0	0	17	-	-	1	36	12	24	0	166	-	57	0	-	-	8	0	155	-	-
		%	-	0.0	0.0	18.9	-	-	25.0	37.1	7.3	33.3	0.0	36.4	-	34.8	0.0	-	-	16.0	0.0	46.5	-	-
B. Beverages	Total no. of products		5	0	3	0	25	73	1	0	0	0	47	104	0	0	53	3	0	72	0	56	3	24
	Saturated fat	n	0	-	0	-	0	1	0	-	-	-	0	4	-	0	4	0	-	5	-	0	0	0
		%	0.0	-	0.0	-	0.0	1.4	0.0	-	-	-	0.0	3.8	-	0.0	7.5	0.0	-	6.9	-	0.0	0.0	0.0
	Sodium	n	0	-	0	-	0	9	0	-	-	-	0	0	-	0	0	0	-	0	-	0	0	0
		%	0.0	-	0.0	-	0.0	12.3	0.0	-	-	-	0.0	0.0	-	0.0	0.0	0.0	-	0.0	-	0.0	0.0	0.0
	Total sugars	n	2	-	3	-	17	43	0	-	-	-	6	49	-	0	20	0	-	47	-	25	2	7
		%	40.0	-	100.0	-	68.0	58.9	0.0	-	-	-	12.8	47.1	-	0.0	37.7	0.0	-	65.3	-	44.6	66.7	29.2
C. Cereals and other grain products	Total no. of products		0	0	0	0	0	0	0	33	4	45	4	230	0	1	0	0	0	46	0	78	0	13
	Saturated fat	n	-	-	-	-	-	-	-	0	1	1	0	12	-	0	-	-	-	4	-	7	-	0
		%	-	-	-	-	-	-	-	0.0	25.0	2.2	0.0	5.2	-	0.0	-	-	-	8.7	-	9.0	-	0.0
	Sodium	n	-	-	-	-	-	-	-	2	1	1	3	26	-	0	-	-	-	4	-	12	-	12
		%	-	-	-	-	-	-	-	6.1	25.0	2.2	75.0	11.3	-	0.0	-	-	-	8.7	-	15.4	-	92.3
	Total sugars	n	-	-	-	-	-	-	-	15	1	10	0	8	-	0	-	-	-	6	-	8	-	0
		%	-	-	-	-	-	-	-	45.5	25.0	22.2	0.0	3.5	-	0.0	-	-	-	13.0	-	10.3	-	0.0
D. Dairy products and substitutes	Total no. of products		0	119	6	0	0	0	110	110	0	3	113	226	0	0	19	0	107	0	89	87	0	0
	Saturated fat	n	-	45	1	-	-	-	26	34	-	0	104	162	-	-	6	-	72	-	58	77	-	-
		%	-	37.8	16.7	-	-	-	23.6	30.9	-	0.0	92.0	71.7	-	-	31.6	-	67.3	-	65.2	88.5	-	-
	Sodium	n	-	19	1	-	-	-	0	0	-	0	71	75	-	-	0	-	28	-	25	31	-	-
		%	-	16.0	16.7	-	-	-	0.0	0.0	-	0.0	62.8	33.2	-	-	0.0	-	26.2	-	28.1	35.6	-	-
	Total sugars	n	-	42	4	-	-	-	73	76	-	3	4	34	-	-	17	-	39	-	23	5	-	-
		%	-	35.3	66.7	-	-	-	66.4	69.1	-	100.0	3.5	15.0	-	-	89.5	-	36.4	-	25.8	7.0	-	-
E. Desserts	Total no. of products		0	0	0	0	0	0	17	1	0	0	41	102	0	0	115	0	0	0	0	66	0	62
	Saturated fat	n	-	-	-	-	-	-	7	1	-	-	3	50	-	-	88	-	-	-	-	32	-	51
		%	-	-	-	-	-	-	41.2	100.0	-	-	7.3	49.0	-	-	76.5	-	-	-	-	48.5	-	82.3
	Sodium	n	-	-	-	-	-	-	0	0	-	-	7	1	-	-	0	-	-	-	-	1	-	0
		%	-	-	-	-	-	-	0.0	0.0	-	-	17.1	1.0	-	-	0.0	-	-	-	-	1.5	-	0.0
	Total sugars	n	-	-	-	-	-	-	12	1	-	-	26	88	-	-	96	-	-	-	-	54	-	56
		%	-	-	-	-	-	-	70.6	100.0	-	-	63.4	86.3	-	-	83.5	-	-	-	-	81.8	-	90.3
F. Dessert toppings and fillings	Total no. of products		0	0	0	0	0	0	0	5	0	0	1	14	0	0	0	0	0	0	0	6	0	0
	Saturated fat	n	-	-	-	-	-	-	-	5	-	-	0	1	-	-	-	-	-	-	-	0	-	-
		%	-	-	-	-	-	-	-	100.0	-	-	0.0	7.1	-	-	-	-	-	-	-	0.0	-	-
	Sodium	n	-	-	-	-	-	-	-	0	-	-	0	0	-	-	-	-	-	-	-	0	-	-
		%	-	-	-	-	-	-	-	0.0	-	-	0.0	0.0	-	-	-	-	-	-	-	0.0	-	-
	Total sugars	n	-	-	-	-	-	-	-	5	-	-	1	13	-	-	-	-	-	-	-	6	-	-
		%	-	-	-	-	-	-	-	100.0	-	-	100.0	92.9	-	-	-	-	-	-	-	100.0	-	-
G. Eggs and egg substitutes	Total no. of products		0	0	0	0	0	0	0	0	0	0	0	13	0	0	0	0	0	0	0	3	0	0
	Saturated fat	n	-	-	-	-	-	-	-	-	-	-	-	0	-	-	-	-	-	-	-	0	-	-
		%	-	-	-	-	-	-	-	-	-	-	-	0.0	-	-	-	-	-	-	-	0.0	-	-
	Sodium	n	-	-	-	-	-	-	-	-	-	-	-	0	-	-	-	-	-	-	-	0	-	-
		%	-	-	-	-	-	-	-	-	-	-	-	0.0	-	-	-	-	-	-	-	0.0	-	-
	Total sugars	n	-	-	-	-	-	-	-	-	-	-	-	0	-	-	-	-	-	-	-	0	-	-
		%	-	-	-	-	-	-	-	-	-	-	-	0.0	-	-	-	-	-	-	-	0.0	-	-
H. Fats and oils	Total no. of products		0	3	7	1	0	0	0	0	0	0	85	130	0	0	0	0	13	0	2	69	0	21
	Saturated fat	n	-	3	0	1	-	-	-	-	-	-	1	15	-	-	-	-	10	-	2	13	-	0
		%	-	100.0	0.0	100.0	-	-	-	-	-	-	1.2	11.5	-	-	-	-	76.9	-	100.0	18.8	-	0.0
	Sodium	n	-	0	2	0	-	-	-	-	-	-	63	38	-	-	-	-	0	-	0	23	-	4
		%	-	0.0	28.6	0.0	-	-	-	-	-	-	74.1	29.2	-	-	-	-	0.0	-	0.0	33.3	-	19.0
	Total sugars	n	-	0	0	0	-	-	-	-	-	-	1	1	-	-	-	-	0	-	0	0	-	0
		%	-	0.0	0.0	0.0	-	-	-	-	-	-	1.2	0.8	-	-	-	-	0.0	-	0.0	0.0	-	0.0
I. Marine and fresh water animals	Total no. of products		0	0	0	0	0	0	0	0	0	0	0	87	0	0	0	0	0	0	0	48	0	0
	Saturated fat	n	-	-	-	-	-	-	-	-	-	-	-	14	-	-	-	-	-	-	-	5	-	-
		%	-	-	-	-	-	-	-	-	-	-	-	16.1	-	-	-	-	-	-	-	10.4	-	-
	Sodium	n	-	-	-	-	-	-	-	-	-	-	-	46	-	-	-	-	-	-	-	22	-	-
		%	-	-	-	-	-	-	-	-	-	-	-	52.9	-	-	-	-	-	-	-	45.8	-	-

Food category[1]			A. Lassonde	Agropur	Campbell	Canada Bread	Canada Dry Mott's	Coca-Cola	Danone	General Mills	George Weston	Kellogg	Kraft Heinz	Loblaw	Maple Leaf Foods	Mondelēz	Nestlé	Ocean Spray	Parmalat	PepsiCo	Saputo	Sobeys	Sun-Rype	Unilever
	Total sugars	n	-	-	-	-	-	-	-	-	-	-	-	1	-	-	-	-	-	-	-	0	-	-
		%	-	-	-	-	-	-	-	-	-	-	-	1.1	-	-	-	-	-	-	-	0.0	-	-
J. Fruit and fruit juices	Total no. of products		71	0	11	0	25	65	0	0	0	0	9	201	0	0	0	32	0	24	0	126	18	0
	Saturated fat	n	0	-	0	-	0	0	-	-	-	-	0	1	-	-	-	0	-	0	-	0	0	-
		%	0.0	-	0.0	-	0.0	0.0	-	-	-	-	0.0	0.5	-	-	-	0.0	-	0.0	-	0.0	0.0	-
	Sodium	n	0	-	0	-	0	0	-	-	-	-	0	0	-	-	-	0	-	0	-	0	0	-
		%	0.0	-	0.0	-	0.0	0.0	-	-	-	-	0.0	0.0	-	-	-	0.0	-	0.0	-	0.0	0.0	-
	Total sugars	n	58	-	11	-	11	65	-	-	-	-	7	142	-	-	-	23	-	20	-	84	14	-
		%	81.7	-	100.0	-	44.0	100.0	-	-	-	-	77.8	70.6	-	-	-	71.9	-	83.3	-	66.7	77.8	-
K. Legumes	Total no. of products		0	0	0	0	0	0	0	0	0	0	0	59	2	0	0	0	0	0	0	18	0	0
	Saturated fat	n	-	-	-	-	-	-	-	-	-	-	-	0	0	-	-	-	-	-	-	0	-	-
		%	-	-	-	-	-	-	-	-	-	-	-	0.0	0.0	-	-	-	-	-	-	0.0	-	-
	Sodium	n	-	-	-	-	-	-	-	-	-	-	-	3	1	-	-	-	-	-	-	1	-	-
		%	-	-	-	-	-	-	-	-	-	-	-	5.1	50.0	-	-	-	-	-	-	5.6	-	-
	Total sugars	n	-	-	-	-	-	-	-	-	-	-	-	0	0	-	-	-	-	-	-	0	-	-
		%	-	-	-	-	-	-	-	-	-	-	-	0.0	0.0	-	-	-	-	-	-	0.0	-	-
L. Meat, poultry, their products and substitutes	Total no. of products		0	0	0	0	0	0	0	0	0	0	0	253	126	0	0	0	0	0	0	105	0	0
	Saturated fat	n	-	-	-	-	-	-	-	-	-	-	-	143	60	-	-	-	-	-	-	61	-	-
		%	-	-	-	-	-	-	-	-	-	-	-	56.5	47.6	-	-	-	-	-	-	58.1	-	-
	Sodium	n	-	-	-	-	-	-	-	-	-	-	-	200	114	-	-	-	-	-	-	83	-	-
		%	-	-	-	-	-	-	-	-	-	-	-	79.1	90.5	-	-	-	-	-	-	79.0	-	-
	Total sugars	n	-	-	-	-	-	-	-	-	-	-	-	7	12	-	-	-	-	-	-	6	-	-
		%	-	-	-	-	-	-	-	-	-	-	-	2.8	9.5	-	-	-	-	-	-	5.7	-	-
M. Miscellaneous category	Total no. of products		0	0	0	0	2	0	0	44	1	1	11	81	1	7	5	0	0	17	0	44	0	1
	Saturated fat	n	-	-	-	-	0	-	-	5	0	0	1	19	1	0	0	-	-	1	-	8	-	0
		%	-	-	-	-	0.0	-	-	11.4	0.0	0.0	9.1	23.5	100.0	0.0	0.0	-	-	5.9	-	18.2	-	0.0
	Sodium	n	-	-	-	-	2	-	-	17	1	0	6	17	1	1	5	-	-	4	-	14	-	1
		%	-	-	-	-	100.0	-	-	38.6	100.0	0.0	54.5	21.0	100.0	14.3	100.0	-	-	23.5	-	31.8	-	100.0
	Total sugars	n	-	-	-	-	0	-	-	27	0	0	2	23	0	1	0	-	-	4	-	14	-	0
		%	-	-	-	-	0.0	-	-	61.4	0.0	0.0	18.2	28.4	0.0	14.3	0.0	-	-	23.5	-	31.8	-	0.0
N. Combination dishes	Total no. of products		0	0	6	2	0	0	0	45	0	0	47	243	20	0	84	0	0	0	0	150	0	22
	Saturated fat	n	-	-	0	0	-	-	-	11	-	-	1	102	17	-	41	-	-	-	-	59	-	0
		%	-	-	0.0	0.0	-	-	-	24.4	-	-	2.1	42.0	85.0	-	48.8	-	-	-	-	39.3	-	0.0
	Sodium	n	-	-	6	2	-	-	-	43	-	-	44	171	19	-	70	-	-	-	-	115	-	16
		%	-	-	100.0	100.0	-	-	-	95.6	-	-	93.6	70.4	95.0	-	83.3	-	-	-	-	76.7	-	72.7
	Total sugars	n	-	-	0	0	-	-	-	0	-	-	1	4	1	-	0	-	-	-	-	6	-	0
		%	-	-	0.0	0.0	-	-	-	0.0	-	-	2.1	1.6	5.0	-	0.0	-	-	-	-	4.0	-	0.0
O. Nuts and seeds	Total no. of products		0	0	0	0	0	0	0	0	0	0	11	44	0	0	0	0	0	0	0	28	0	0
	Saturated fat	n	-	-	-	-	-	-	-	-	-	-	0	12	-	-	-	-	-	-	-	12	-	-
		%	-	-	-	-	-	-	-	-	-	-	0.0	27.3	-	-	-	-	-	-	-	42.9	-	-
	Sodium	n	-	-	-	-	-	-	-	-	-	-	0	0	-	-	-	-	-	-	-	0	-	-
		%	-	-	-	-	-	-	-	-	-	-	0.0	0.0	-	-	-	-	-	-	-	0.0	-	-
	Total sugars	n	-	-	-	-	-	-	-	-	-	-	0	0	-	-	-	-	-	-	-	0	-	-
		%	-	-	-	-	-	-	-	-	-	-	0.0	0.0	-	-	-	-	-	-	-	0.0	-	-
P. Potatoes, sweet potatoes and yams	Total no. of products		0	0	0	0	0	0	0	7	0	0	0	23	0	0	0	0	0	0	0	23	0	0
	Saturated fat	n	-	-	-	-	-	-	-	0	-	-	-	2	-	-	-	-	-	-	-	4	-	-
		%	-	-	-	-	-	-	-	0.0	-	-	-	8.7	-	-	-	-	-	-	-	17.4	-	-
	Sodium	n	-	-	-	-	-	-	-	6	-	-	-	8	-	-	-	-	-	-	-	6	-	-
		%	-	-	-	-	-	-	-	85.7	-	-	-	34.8	-	-	-	-	-	-	-	26.1	-	-
	Total sugars	n	-	-	-	-	-	-	-	0	-	-	-	0	-	-	-	-	-	-	-	0	-	-
		%	-	-	-	-	-	-	-	0.0	-	-	-	0.0	-	-	-	-	-	-	-	0.0	-	-
Q. Salads	Total no. of products		0	0	0	0	0	0	0	0	0	0	0	32	0	0	0	0	0	0	0	29	0	0
	Saturated fat	n	-	-	-	-	-	-	-	-	-	-	-	18	-	-	-	-	-	-	-	0	-	-
		%	-	-	-	-	-	-	-	-	-	-	-	56.3	-	-	-	-	-	-	-	0.0	-	-
	Sodium	n	-	-	-	-	-	-	-	-	-	-	-	18	-	-	-	-	-	-	-	5	-	-
		%	-	-	-	-	-	-	-	-	-	-	-	56.3	-	-	-	-	-	-	-	17.2	-	-
	Total sugars	n	-	-	-	-	-	-	-	-	-	-	-	6	-	-	-	-	-	-	-	1	-	-
		%	-	-	-	-	-	-	-	-	-	-	-	18.8	-	-	-	-	-	-	-	3.4	-	-
R. Sauces, dips, gravies and condiments	Total no. of products		0	0	21	0	0	0	0	7	0	0	80	258	1	0	3	2	0	19	4	83	0	19
	Saturated fat	n	-	-	0	-	-	-	-	0	-	-	2	30	0	-	0	0	-	0	0	6	-	2
		%	-	-	0.0	-	-	-	-	0.0	-	-	2.5	11.6	0.0	-	0.0	0.0	-	0.0	0.0	7.2	-	10.5
	Sodium	n	-	-	8	-	-	-	-	6	-	-	65	102	0	-	3	0	-	5	0	39	-	4
		%	-	-	38.1	-	-	-	-	85.7	-	-	81.3	39.5	0.0	-	100.0	0.0	-	26.3	0.0	47.0	-	21.1

Food category[1]			A. Lassonde	Agropur	Campbell	Canada Bread	Canada Dry Mott's	Coca-Cola	Danone	General Mills	George Weston	Kellogg	Kraft Heinz	Loblaw	Maple Leaf Foods	Mondelēz	Nestlé	Ocean Spray	Parmalat	PepsiCo	Saputo	Sobeys	Sun-Rype	Unilever
	Total sugars	n	-	-	1.0	-	-	-	-	0.0	-	-	12.0	41.0	0.0	-	2.0	2.0	-	0.0	0.0	20.0	-	1.0
		%	-	-	4.8	-	-	-	-	0.0	-	-	15.0	15.9	0.0	-	66.7	100.0	-	0.0	0.0	24.1	-	5.3
S. Snacks	Total no. of products		0	0	0	3	0	0	0	7	0	17	0	145	8	6	1	1	0	109	0	79	0	0
	Saturated fat	n	-	-	-	3	-	-	-	3	-	15	-	56	6	0	1	1	-	14	-	29	-	-
		%	-	-	-	100.0	-	-	-	42.9	-	88.2	-	38.6	75.0	0.0	100.0	100.0	-	12.8	-	36.7	-	-
	Sodium	n	-	-	-	1	-	-	-	3	-	4	-	38	8	6	0	0	-	43	-	17	-	-
		%	-	-	-	33.3	-	-	-	42.9	-	23.5	-	26.2	100.0	100.0	0.0	0.0	-	39.4	-	21.5	-	-
	Total sugars	n	-	-	-	0	-	-	-	0	-	0	-	13	0	0	0	1	-	9	-	5	-	-
		%	-	-	-	0.0	-	-	-	0.0	-	0.0	-	9.0	0.0	0.0	0.0	100.0	-	8.3	-	6.3	-	-
T. Soups	Total no. of products		0	0	135	0	0	0	0	0	0	0	0	66	0	0	0	0	0	0	0	41	0	51
	Saturated fat	n	-	-	23	-	-	-	-	-	-	-	-	15	-	-	-	-	-	-	-	13	-	0
		%	-	-	17.0	-	-	-	-	-	-	-	-	22.7	-	-	-	-	-	-	-	31.7	-	0.0
	Sodium	n	-	-	130	-	-	-	-	-	-	-	-	64	-	-	-	-	-	-	-	39	-	51
		%	-	-	96.3	-	-	-	-	-	-	-	-	97.0	-	-	-	-	-	-	-	95.1	-	100.0
	Total sugars	n	-	-	7	-	-	-	-	-	-	-	-	0	-	-	-	-	-	-	-	1	-	0
		%	-	-	5.2	-	-	-	-	-	-	-	-	0.0	-	-	-	-	-	-	-	2.4	-	0.0
U. Sugars and sweets	Total no. of products		0	0	0	0	4	0	0	17	0	0	8	132	0	49	32	0	0	3	0	55	15	0
	Saturated fat	n	-	-	-	-	0	-	-	0	-	-	5	33	-	40	27	-	-	0	-	10	0	-
		%	-	-	-	-	0.0	-	-	0.0	-	-	62.5	25.0	-	81.6	84.4	-	-	0.0	-	18.2	0.0	-
	Sodium	n	-	-	-	-	0	-	-	0	-	-	0	0	-	0	0	-	-	0	-	0	0	-
		%	-	-	-	-	0.0	-	-	0.0	-	-	0.0	0.0	-	0.0	0.0	-	-	0.0	-	0.0	0.0	-
	Total sugars	n	-	-	-	-	2	-	-	15	-	-	6	96	-	47	28	-	-	0	-	43	15	-
		%	-	-	-	-	50.0	-	-	88.2	-	-	75.0	72.7	-	95.9	87.5	-	-	0.0	-	78.2	100.0	-
V. Vegetables	Total no. of products		1	0	6	0	8	0	0	9	0	0	3	200	0	0	0	0	0	0	0	106	0	4
	Saturated fat	n	0	-	0	-	0	-	-	0	-	-	0	4	-	-	-	-	-	-	-	1	-	0
		%	0.0	-	0.0	-	0.0	-	-	0.0	-	-	0.0	2.0	-	-	-	-	-	-	-	0.9	-	0.0
	Sodium	n	1	-	2	-	7	-	-	0	-	-	1	45	-	-	-	-	-	-	-	24	-	3
		%	100.0	-	33.3	-	87.5	-	-	0.0	-	-	33.3	22.5	-	-	-	-	-	-	-	22.6	-	75.0
	Total sugars	n	0	-	1	-	0	-	-	0	-	-	0	2	-	-	-	-	-	-	-	2	-	0
		%	0.0	-	16.7	-	0.0	-	-	0.0	-	-	0.0	1.0	-	-	-	-	-	-	-	1.9	-	0.0

[1]Food categories are defined in Health Canada's Table of Reference Amounts for Foods. If none of the sampled products fell into that food category, it was omitted from the table.

[2]The number of products offered by the company in that food category (that were included in the FLIP 2017 database). Dashes indicate that the company did not offer products within that food category.

[3]The number of products offered by the company that exceeded Health Canada's 15% Daily Value threshold for saturated fat, sodium or total sugars (≥30% of the Daily Value for prepackaged meals and main dishes).

[4]The percentage of products offered by the company that exceeded Health Canada's 15% Daily Value threshold for saturated fat, sodium or total sugars (≥30% of the Daily Value for prepackaged meals and main dishes).
